# A Mechanistic Neural Field Theory of How Anesthesia Suppresses Consciousness: Synaptic Drive Dynamics, Bifurcations, Attractors, and Partial State Equipartitioning

**DOI:** 10.1186/s13408-015-0032-7

**Published:** 2015-10-05

**Authors:** Saing Paul Hou, Wassim M. Haddad, Nader Meskin, James M. Bailey

**Affiliations:** A*STAR, Singapore Institute of Manufacturing Technology, Singapore, 638075 Singapore; School of Aerospace Engineering, Georgia Institute of Technology, Atlanta, GA 30332 USA; Electrical Engineering Department, Qatar University, Doha, Qatar; Department of Anesthesiology, Northeast Georgia Medical Center, Gainesville, GA 30503 USA

**Keywords:** Dynamical systems, Hopf bifurcation, Biological networks, Spiking neuron models, Synaptic drives, Mean field models, Syncrhonization, Equipartition, Consciousness, General anesthesia

## Abstract

With the advances in biochemistry, molecular biology, and neurochemistry there has been impressive progress in understanding the molecular properties of anesthetic agents. However, there has been little focus on how the molecular properties of anesthetic agents lead to the observed macroscopic property that defines the anesthetic state, that is, lack of responsiveness to noxious stimuli. In this paper, we use dynamical system theory to develop a mechanistic mean field model for neural activity to study the abrupt transition from consciousness to unconsciousness as the concentration of the anesthetic agent increases. The proposed synaptic drive firing-rate model predicts the conscious–unconscious transition as the applied anesthetic concentration increases, where excitatory neural activity is characterized by a Poincaré–Andronov–Hopf bifurcation with the awake state transitioning to a stable limit cycle and then subsequently to an asymptotically stable unconscious equilibrium state. Furthermore, we address the more general question of synchronization and partial state equipartitioning of neural activity without mean field assumptions. This is done by focusing on a postulated subset of inhibitory neurons that are not themselves connected to other inhibitory neurons. Finally, several numerical experiments are presented to illustrate the different aspects of the proposed theory.

## Introduction

The in vitro effects of anesthetic agents have been intensely investigated, and the actions of anesthetic agents on single neurons have been described [1, 2]. There is compelling evidence that at least some modern anesthetics alter postsynaptic potentials. A key goal of anesthetic and neuroscience research is to understand how this effect on the single neuron translates into an abrupt transition from consciousness to unconsciousness as the concentration of the agent increases [3–7].

A rigorous analysis of the effect of anesthetic agents ideally would be within the context of the Hodgkins–Huxley nonlinear differential equations describing the electrical characteristics of neurons [8, 9]. However, the complexity of this model limits its tractability for systems of multiple neurons. Since the seminal publication of Wilson and Cowan [10] it has been common to instead utilize *firing-rate models*. However, even with the considerable mathematical simplifications afforded by the assumptions of firing-rate models, the immense dimensionality of the brain requires further assumptions and approximations. Typically, firing-rate models are simplified further by either mean field assumptions, which postulate that the brain is organized into a limited number of populations of identical neurons, or by assuming that the strength of connections between neurons have a specific (typically normal) distribution. In some instances the firing-rate model is further simplified by postulating specific network architectures [11].

While firing-rate models have been extensively investigated in the neuroscience literature, there has been limited applications of these models to the effects of anesthetic agents. One of the earlier investigations came from Steyn-Ross et al. [12], who extended a generalized mean field firing-rate model that postulates specific short range and long range connections between cortical neurons. This model was originally developed by Liley et al. [13] to analyze the effects of anesthetic agents on the mean electrical potential of the cell body of neurons. The model proposed in [12] predicts that the transition from consciousness to unconsciousness induced by anesthetic agents can be characterized as a first-order phase transition to a state of decreased neural activity. However, the significance of decreased neural activity for the transition from consciousness to unconsciousness is unclear.

Anesthetic agents typically cause paradoxical increases in the activity of the brain, sometimes to the point of seizure activity, before leading to decreased activity. A primary experimental modality for investigating the induction of anesthesia is the electroencephalogram (EEG). This is manifested in the EEG where anesthetic agents typically cause an increase in the amplitudes of certain frequency bands at the point of transition to unconsciousness with subsequent burst suppression and eventually a flat line EEG as the anesthetic concentration increases. There has been significant interest in understanding the effects of anesthetic agents on the EEG [2–4, 14–17] and the theoretical models can predict this paradoxical increase in neural activity observed experimentally.

In contrast to the phase transition model proposed in [12], another general theory of the loss of consciousness is that it reflects loss of information flow between different parts of the brain [18]. John and Prichep [19] have proposed a model of specific sequential neuroanatomic targets for anesthetic agents, originating in the reticular activating system and progressing through the mesolimbic system, with closure of thalamic gates and blockade of thalamocortical reverberations and uncoupling of parietal-frontal interactions with loss of consciousness. It is plausible that a decrease of neural activity in specific segments or neuronal populations of the brain could lead to the loss of information flow and there is experimental evidence to support this hypothesis [20].

In this paper, we use dynamical system theory to develop a mechanistic mean field model for neural activity. Our focus here is to understand general neural activity without attempting to predict the anatomic specificity of the John and Prichep theory [19]. Specifically, using the notion of synaptic drive—a measure of neuronal population activity that captures the influence of a given neuron population on the behavior of the neuronal network—and topological system stability, we show that this simplified model predicts decreasing mean excitatory neural activity with increasing anesthetic potency at the level of the single neuron. We seek conditions that lead to decreasing excitatory neural activity despite the reservations noted above around the role of decreasing activity for the induction of anesthesia for two reasons. First, as noted above, it is plausible that decreasing excitatory activity in selected subpopulations of the brain could lead to the interruption of information flow that may characterize unconsciousness. Second, anesthetic agents do eventually lead to decreased activity as the concentration of the anesthetic agent increases. While we would expect this of any phenomenological model for addressing how anesthesia suppresses consciousness, the novelty of the model is the additional prediction of a conscious–unconscious transition as the applied anesthetic concentration increases, where excitatory neural activity is characterized by a stable limit cycle. Then, as the anesthetic concentration increases further, the proposed dynamical system model undergoes a supercritical reverse Hopf bifurcation and transitions from a limit cycle behavior to an asymptotically stable equilibrium corresponding to an anesthetized state, where the excitatory neuron activity is very low.

Next, we extend our mean field theory to incorporate a more complex model for the postsynaptic potential. Specifically, by using an Erlang-type time multiplied exponential decay model for the postsynaptic potential rather than a simple exponential decay [11, 13], we can account for the delay in peak amplitude of the postsynaptic potential that occurs after a neuron discharges ([11], Fig. 8.5). In this case, we see biphasic responses in the mean neural activity that seems to parallel the rise and decay of the postsynaptic potential. The mean field synaptic drive model we present is based on the assumption that, within the populations of excitatory and inhibitory neurons, second-order terms reflecting the variation of synaptic connection strengths and the variation of synaptic drives can be ignored [5].

Finally, we investigate the synaptic drive model without mean field assumptions by postulating the existence of a subpopulation of inhibitory neurons that themselves do not receive inputs from other inhibitory neurons. In this case, we provide sufficient conditions for global asymptotic and exponential partial synaptic drive equipartitioning for our excitatory and inhibitory cortical neuronal network. Specifically, we show that as the inhibitory time constants increase (one of the demonstrated in vitro effects of some anesthetic agents [1, 2]), the excitatory neurons that are coupled to inhibitory neurons all approach a zero synaptic drive state, whereas the inhibitory neurons that themselves are not coupled to inhibitory neurons converge to a constant synaptic drive state.

The notation used in this paper is fairly standard. Specifically, for $x\in\mathbb{R}^{n}$, we write $x\geq\geq0$ (respectively, $x\gg 0$) to indicate that every component $x_{i}$ of *x* is nonnegative (respectively, positive). In this case, we say that *x* is *nonnegative* or *positive*, respectively. Likewise, $A\in\mathbb {R}^{n\times m}$ is *nonnegative* or *positive* if every entry of *A* is nonnegative or positive, respectively, which is written as $A\geq\geq0$ or $A\gg 0$, respectively. For $A\in\mathbb{R}^{n\times n}$, we write $A\geq0$ (respectively, $A>0$) to indicate that *A* is nonnegative (respectively, positive) definite. Furthermore, we write $\overline{\mathbb{R}}^{n}_{+}$ and $\mathbb{R}^{n}_{+}$ to denote the nonnegative and positive orthants of $\mathbb{R}^{n}$, that is, if $x\in \mathbb{R}^{n}$, then $x\in\overline{\mathbb{R}}^{n}_{+}$ and $x\in\mathbb {R}^{n}_{+}$ are equivalent, respectively, to $x\geq\geq0$ and $x\gg 0$. In addition, we write $0_{n\times m}$ to denote the $n\times m$ zero matrix, $I_{n}$ to denote the $n\times n$ identity matrix, and $\mathbf {e}_{n}\in\mathbb{R}^{n}$ to denote the ones vector of order *n*, that is, $\mathbf{e}_{n}=[1,1,\ldots,1]^{\mathrm{T}}$; if the order of $\mathbf{e}_{n}$ is clear from the context we simply write **e** for $\mathbf {e}_{n}$. Finally, we write $(\cdot)^{\mathrm{T}}$ to denote the transpose, $\operatorname {tr}(\cdot)$ to denote the trace, $\operatorname {det}(\cdot)$ to denote the determinant, and $(\cdot)'$ to denote the Fréchet derivative.

## A Model for Excitatory and Inhibitory Neural Populations

Biological neural network models predict a voltage in the receiving or postsynaptic neuron given by [7, 9]

1 $$\begin{aligned} v_{i}^{\mathrm{X}}(t)&=\sum_{j=1}^{n_{\mathrm{E}}}A_{ij}^{\mathrm{XE}} \sum_{k}\alpha _{j}^{\mathrm{E}}(t-t_{k})+ \sum_{j'=1}^{n_{\mathrm{I}}}A_{ij'}^{\mathrm{XI}} \sum_{k'}\alpha _{j'}^{\mathrm{I}}(t-t_{k'}), \end{aligned}$$

where $v_{i}^{\mathrm{X}}(\cdot)$, $i=1,\ldots,n_{\mathrm{X}}$, $\mathrm{X}\in\{\mathrm{E}, \mathrm{I}\}$, is the excitatory ($\mathrm{X}=\mathrm{E}$) and inhibitory ($\mathrm{X}=\mathrm{I}$) voltage in the *i*th receiving neuron, $A_{ij}^{\mathrm{XY}}$, X, $\mathrm{Y}\in\{\mathrm{E},\mathrm{I}\}$, are constants representing the coupling strengths (in volts) of the *j*th neuron on the *i*th neuron, $k,k'=1,\ldots$ , enumerate the action potential or firings of the excitatory and inhibitory transmitting (presynaptic) neurons at firing times $t_{k}$ and $t_{k'}$, respectively, and $\alpha_{j}^{\mathrm{E}}(\cdot)$ and $\alpha_{j'}^{\mathrm{I}}(\cdot)$ are dimensionless functions describing the evolution of the excitatory and inhibitory postsynaptic potentials, respectively. Using a (possibly discontinuous) function $f_{i}(\cdot)$ to represent the firing rate (in Hz) of the *i*th neuron that determines the instantaneous number of spikes that a neuron fires over an infinitesimal time interval $[t,t+\mathrm{d}t]$, and assuming that the firing rate is a function of the voltage $v_{i}^{\mathrm{E}}(\cdot)$ (resp., $v_{i}^{\mathrm{I}}(\cdot)$) across the membrane of the *i*th neuron given by $f_{i}(v_{i}^{\mathrm{E}})$ (resp., $f_{i}(v_{i}^{\mathrm{I}})$), it follows from (1) that

2 $$\begin{aligned} v_{i}^{\mathrm{E}}(t)={}&\sum _{j=1,j\neq i}^{n_{\mathrm{E}}}A_{ij}^{\mathrm{EE}}\int _{-\infty}^{t}\alpha_{j}^{\mathrm{E}}(t- \tau)f_{j}\bigl(v_{j}^{\mathrm{E}}(\tau)\bigr)\, \mathrm{d}\tau \\ &{}+\sum_{j'=1}^{n_{\mathrm{I}}}A_{ij'}^{\mathrm{EI}} \int_{-\infty}^{t}\alpha _{j'}^{\mathrm{I}}(t- \tau)f_{j'}\bigl(v_{j'}^{\mathrm{I}}(\tau)\bigr)\, \mathrm{d}\tau+v_{\mathrm{th}i}^{\mathrm{E}}(t), \\ &{} \qquad\qquad\qquad\qquad\qquad\qquad\qquad\qquad\qquad i=1,\ldots,n_{\mathrm{E}}, \end{aligned}$$

3 $$\begin{aligned} v_{i}^{\mathrm{I}}(t)={}&\sum _{j=1}^{n_{\mathrm{E}}}A_{ij}^{\mathrm{IE}}\int _{-\infty }^{t}\alpha_{j}^{\mathrm{E}}(t- \tau)f_{j}\bigl(v_{j}^{\mathrm{E}}(\tau)\bigr)\, \mathrm{d}\tau \\ &{}+\sum_{j'=1,j'\neq i}^{n_{\mathrm{I}}}A_{ij'}^{\mathrm{II}} \int_{-\infty }^{t}\alpha_{j'}^{\mathrm{I}}(t- \tau)f_{j'}\bigl(v_{j'}^{\mathrm{I}}(\tau)\bigr)\, \mathrm{d}\tau +v_{\mathrm{th}i}^{\mathrm{I}}(t), \\ &{}\qquad\qquad\qquad\qquad\qquad\qquad\qquad\qquad\qquad i=1,\ldots,n_{\mathrm{I}}, \end{aligned}$$

where $f_{j}(v_{j}^{\mathrm{X}}(t))\,\mathrm{d}t$, $\mathrm{X}\in\{ \mathrm{E},\mathrm{I}\}$, is the probability of a spike occurring in the time interval $[t,t+\mathrm{d}t]$, and $A_{ij}^{\mathrm{XY}}$, $\mathrm{X},\mathrm{Y}\in\{\mathrm{E},\mathrm{I}\}$, are *neural connectivity weights*, with units of volts, representing the coupling strength of the *j*th neuron on the *i*th neuron such that $A_{ij}^{\mathrm{XE}}>0$ and $A_{ij}^{\mathrm{XI}}<0$, $\mathrm{X}\in\{\mathrm{E},\mathrm{I}\}$, if the *j*th neuron is connected (i.e., contributes a postsynaptic potential) to the *i*th neuron, and $A_{ij}^{\mathrm{XY}}=0$, otherwise. Alternatively, $f_{i}(\cdot)$ can represent an ensemble average of firing rates of neurons in a single population *i* over $[t,t+\mathrm{d}t]$. In this case, the firing rate of the population is exactly the same as that of an individual neuron and (2) and (3) represent excitatory and inhibitory voltages for the *i*th population. Furthermore, $v_{\mathrm{th}i}^{\mathrm{E}}(\cdot)$ and $v_{\mathrm{th}i}^{\mathrm{I}}(\cdot)$ are continuous input threshold voltages characterizing nerve impulses from sensory (pain) receptors, thermo (temperature sensing) receptors, or proprioceptive (motion sensing) receptors. Alternatively, $v_{\mathrm{th}i}^{\mathrm{E}}(\cdot)$ and $v_{\mathrm{th}i}^{\mathrm{I}}(\cdot)$ can be thought of as inputs from the reticular activating system within the brainstem responsible for regulating arousal and sleep–wake transitions. Note that $A_{ii}^{\mathrm{EE}}\triangleq A_{ii}^{\mathrm{II}}\triangleq0$ by definition.

Next, defining the *synaptic drive*—a dimensionless quantity—of each (excitatory or inhibitory) neuron as the convolution of the presynaptic firing rate with the postsynaptic potential given by [7, 9]

4 $$ S_{i}^{(\mathrm{E},\mathrm{I})}(t)\triangleq\int_{-\infty}^{t} \alpha_{i}^{(\mathrm{E},\mathrm{I})}(t-\tau)f_{i} \bigl(v_{i}^{(\mathrm{E},\mathrm{I})}(\tau)\bigr)\,\mathrm{d}\tau, $$

and assuming an exponential decay of the synaptic voltages of the form [9]

5 $$ \alpha_{i}^{(\mathrm{E},\mathrm{I})}(t)=B_{i}^{(\mathrm{E},\mathrm{I})} e^{-\frac{t}{\lambda_{i}^{(\mathrm{E},\mathrm{I})}}}, $$

where the dimensionless gain $B_{i}^{(\mathrm{E},\mathrm{I})}$ is equal to $B_{i}^{\mathrm{E}}$ if the *i*th neuron is excitatory and $B_{i}^{\mathrm{I}}$ if the *i*th neuron is inhibitory, and similarly for $S_{i}^{(\mathrm{E},\mathrm{I})}$, $v_{i}^{\mathrm{E},\mathrm{I}}$, $\alpha_{i}^{(\mathrm{E},\mathrm{I})}$, and $\lambda_{i}^{(\mathrm{E},\mathrm{I})}$, it follows from (4) and (5) that

6 $$\begin{aligned} \frac{\mathrm{d}S_{i}^{\mathrm{E}}(t)}{\mathrm{d}t}={}&{-}\frac{1}{\lambda_{i}^{\mathrm{E}}}S_{i}^{\mathrm{E}}(t)+B_{i}^{\mathrm{E}}f_{i} \Biggl(\sum_{j=1,j\neq i}^{n_{\mathrm{E}}}A_{ij}^{\mathrm{EE}}S_{j}^{\mathrm{E}}(t)+ \sum_{j'=1}^{n_{\mathrm{I}}}A_{ij'}^{\mathrm{EI}}S_{j'}^{\mathrm{I}}(t)+v_{\mathrm{th}i}^{\mathrm{E}}(t) \Biggr), \\ &{} \qquad\qquad\qquad\qquad\qquad\qquad\qquad\qquad\qquad i=1,\ldots ,n_{\mathrm{E}}, \end{aligned}$$

7 $$\begin{aligned} \frac{\mathrm{d}S_{i}^{\mathrm{I}}(t)}{\mathrm{d}t}={}&{-}\frac{1}{\lambda_{i}^{\mathrm{I}}}S_{i}^{\mathrm{I}}(t)+B_{i}^{\mathrm{I}}f_{i} \Biggl(\sum_{j=1}^{n_{\mathrm{E}}}A_{ij}^{\mathrm{IE}}S_{j}^{\mathrm{E}}(t)+ \sum_{j'=1,j'\neq i}^{n_{\mathrm{I}}}A_{ij'}^{\mathrm{II}}S_{j'}^{\mathrm{I}}(t)+v_{\mathrm{th}i}^{\mathrm{I}}(t) \Biggr), \\ & {}\qquad\qquad\qquad\qquad\qquad\qquad\qquad\qquad\qquad i=1,\ldots ,n_{\mathrm{I}}. \end{aligned}$$

A more general Erlang-type time multiplied exponential decay model for the postsynaptic potential is considered in Sect. 5.

Note that the synaptic drive accounts for pre- and postsynaptic potentials in a neural network to give a measure of neural activity in the network. In particular, it follows from (4) that the synaptic drive quantifies the present activity (via the firing rate) along with all previous activity within a neural population appropriately scaled (via a temporal decay) by when a particular firing event occurred. Hence, the synaptic drive provides a measure of neuronal population activity that captures the influence of a given neuron population on the behavior of the network from the infinite past to the current time instant.

The above analysis reveals that a form for capturing the neuroelectric behavior of biological excitatory and inhibitory neuronal networks can be written as

8 $$\begin{aligned} &\frac{\mathrm{d}S_{i}(t)}{\mathrm{d}t}=-\frac{1}{\tau_{i}}S_{i}(t)+B_{i}f_{i} \Biggl(\sum_{j=1}^{n}A_{ij}S_{j}(t)+v_{\mathrm{th}i}(t) \Biggr), \\ &\qquad\qquad\qquad\qquad\qquad S_{i}(0)=S_{i0},\quad t\geq0,\quad i=1,\ldots,n, \end{aligned}$$

where $S_{i}(t)\in\mathcal{D}\subseteq\mathbb{R}$, $t\geq0$, is the *i*th synaptic drive, $v_{\mathrm{th}i}(t)\in\mathbb{R}$, $t\geq0$, denotes the input threshold voltage to the *i*th neuron, $A_{ij}$ is a constant representing the coupling strength of the *j*th neuron on the *i*th neuron, $\tau_{i}=\lambda_{i}$ is a synaptic time scale, $B_{i}$ is a constant gain for the firing rate of the *i*th neuron, and $f_{i}(\cdot)$ is a nonlinear activation function describing the relationship between the synaptic drive and the firing rate of the *i*th neuron.

Even though the computation of the neural connectivity matrix and the graph topology of human brain networks are intractable, the phenomenological firing-rate model (6) and (7) involving the averaged behavior of the spiking rates of groups of neurons can be used to predict network system changes due to changes in a subset of the *neuronal connectivity matrix*$A^{\mathrm{XY}}$ containing the entries $A^{\mathrm{XY}}_{ij}$ in order to understand how the large neuron population changes qualitatively with the induction of anesthesia. The relevance of the model to realistic systems can be appraised by its prediction of salient aspects of anesthesia. In particular, predicting the abrupt transition from consciousness to unconsciousness resembling a phase transition as well as the biphasic response during induction of anesthesia leading to a paradoxical phase prior to the loss of consciousness [21].

In such population models, the activity of a neuron (population), that is, the rate at which the neuron (population) generates an action potential (i.e., “fires”) is modeled as a function of the voltage (across the membrane). In this paper, we will assume continuous half-wave rectification activation functions as well as smooth sigmoidal functions. Specifically, for a typical neuron [22]

9 $$ f_{i}(x)=[x]_{+}, $$

where $i\in\{1,\ldots,n\}$ and $[x]_{+}=x$ if $x\geq0$, and $[x]_{+}=0$, otherwise. The activation function (9) reflects the fact that as the voltage increases across the membrane of the *i*th neuron, the firing rate increases as well. Often, the membrane potential firing-rate curve exhibits a linear characteristic for a given range of the voltages. At higher voltages, however, a saturation phenomenon appears, indicating that the full effect of the firing rate has been reached. To capture this effect, $f_{i}(\cdot)$ can be modeled as a smooth (i.e., infinitely differentiable) sigmoidal function

10 $$ f_{i}(x)=\frac{f_{\max}e^{\gamma x}}{1+e^{\gamma x}}, $$

where $i\in\{1,\dots,n\}$, $\gamma\gg0$, and $f_{\mathrm{max}}=\lim_{x \to \infty}f_{i}(x)$ denotes the maximum firing rate.

## A Two-Class Mean Excitatory and Mean Inhibitory Synaptic Drive Model

To avoid the complexity of the large-scale neural network model (6) and (7), in this section we consider a mean field model. Specifically, the excitatory and inhibitory synaptic drive model given by (6) and (7) can be reduced to a two-class mean excitatory and mean inhibitory model. In particular, with continuously differentiable $f_{i}(\cdot)=f(\cdot)$, $B_{i}^{\mathrm{E}}=B_{i}^{\mathrm{I}}=1$, $\lambda_{i}^{\mathrm{E}}=\lambda^{\mathrm{E}}$, and $\lambda_{i}^{\mathrm{I}}=\lambda ^{\mathrm{I}}$, (6) and (7) collapse to (see [7] for details)

11 $$\begin{aligned} \frac{\mathrm{d}\overline{S}^{\mathrm{E}}(t)}{\mathrm{d}t}&=\overline {f}_{1}\bigl( \overline{S}^{\mathrm{E}}(t),\overline{S}^{\mathrm{I}}(t)\bigr),\quad \overline {S}^{\mathrm{E}}(0)=\overline{S}^{\mathrm{E}}_{0},\quad t \geq0, \end{aligned}$$

12 $$\begin{aligned} \frac{\mathrm{d}\overline{S}^{\mathrm{I}}(t)}{\mathrm{d}t}&=\overline {f}_{2}\bigl( \overline{S}^{\mathrm{E}}(t), \overline{S}^{\mathrm{I}}(t)\bigr),\quad \overline {S}^{\mathrm{I}}(0)=\overline{S}^{\mathrm{I}}_{0}, \end{aligned}$$

where

13 $$\begin{aligned} \overline{f}_{1}\bigl(\overline{S}^{\mathrm{E}}, \overline{S}^{\mathrm{I}}\bigr)&=f\bigl(a\overline {S}^{\mathrm{E}}-b \overline{S}^{\mathrm{I}}+v_{\mathrm{th}}^{\mathrm{E}}\bigr)- \frac{1}{\lambda ^{\mathrm{E}}}\overline{S}^{\mathrm{E}}, \end{aligned}$$

14 $$\begin{aligned} \overline{f}_{2}\bigl(\overline{S}^{\mathrm{E}}, \overline{S}^{\mathrm{I}}\bigr)&=f\bigl(c\overline {S}^{\mathrm{E}} -d \overline{S}^{\mathrm{I}}+v_{\mathrm{th}}^{\mathrm{I}}\bigr)- \frac{1}{\lambda^{\mathrm{I}}}\overline{S}^{\mathrm{I}}, \end{aligned}$$

$a\triangleq n_{\mathrm{E}}\overline{A}^{\mathrm{EE}}$, $b\triangleq-n_{\mathrm{I}}\overline{A}^{\mathrm{EI}}$, $c\triangleq n_{\mathrm{E}}\overline{A}^{\mathrm{IE}}$, $d\triangleq-n_{\mathrm{I}}\overline{A}^{\mathrm{II}}$, $\overline{A}^{\mathrm{EE}}=A_{ij}^{\mathrm{EE}}-\Delta_{ij}^{\mathrm{EE}}$, $\overline{A}^{\mathrm{EI}}=A_{ij}^{\mathrm{EI}}-\Delta_{ij}^{\mathrm{EI}}$, $\overline{A}^{\mathrm{IE}}=A_{ij}^{\mathrm{IE}}-\Delta_{ij}^{\mathrm{IE}}$, $\overline{A}^{\mathrm{II}}=A_{ij}^{\mathrm{II}}-\Delta_{ij}^{\mathrm{II}}$, $\overline{A}^{\mathrm{XY}}\triangleq\frac{1}{n_{\mathrm{X}}n_{\mathrm{Y}}}\sum_{i=1}^{n_{\mathrm{X}}}\sum_{j=1}^{n_{\mathrm{Y}}}A_{ij}^{\mathrm{XY}}$, X, $\mathrm{Y}\in\{\mathrm{E}, \mathrm{I}\}$, denote the mean, $\Delta_{ij}^{\mathrm{XY}}$, X, $\mathrm{Y}\in\{\mathrm{E}, \mathrm{I}\}$, are deviations from the mean, and $\overline{S}^{\mathrm{E}}(t)\triangleq\frac {1}{n_{\mathrm{E}}}\sum_{j=1}^{n_{\mathrm{E}}}S_{j}^{\mathrm{E}}(t)$ and $\overline {S}^{\mathrm{I}}(t)\triangleq\frac{1}{n_{\mathrm{I}}}\sum_{j=1}^{n_{\mathrm{I}}}S_{j}^{\mathrm{I}}(t)$ denote the mean excitatory synaptic drive and mean inhibitory synaptic drive in dimensionless units, respectively. Note that the constants *a*, *b*, *c*, and *d* are nonnegative. Equations (11) and (12) represent the spatial average (mean) dynamics of the system given by (6) and (7), and they are predicated on a mean field assumption that reduces the complex (approximately $10^{11}\times10^{11}$) neuronal connectivity matrix to a $2\times2$ excitatory–inhibitory system. This is a drastic assumption, but one which has been commonly used in theoretical neuroscience going back to the pioneering work of Wilson and Cowan [10].

To study the dynamic behavior of the mean field model (11) and (12), we assume the postsynaptic activation function is given by (10). The next set of propositions and theorem present several results on the dynamic behavior of (11) and (12). Here, we use the language of topological dynamics (i.e., flows, equilibria, periodic orbits, limit sets) to study the dynamic behavior of (11) and (12). Recall that a set $\mathcal {D}\subseteq\mathbb{R}^{2}$ is *positively invariant* with respect to (11) and (12) if $\mathcal{D}$ contains the orbits of all its points. Moreover, recall the standard *Lyapunov* and *asymptotic stability* definitions for an equilibrium point of (11) and (12) given in [23], and recall that if all solutions of (11) and (12) are bounded, then it follows from the Peano–Cauchy theorem ([23], p. 76), that the maximal solution to (11) and (12) exists on the semi-infinite interval $[0,\infty)$, and hence, (11) and (12) are *forward complete*.

The following propositions are needed for the main result of this section.

### Proposition 1

[7]

*Consider the two*-*class mean excitatory and mean inhibitory synaptic drive network given by* (11) *and* (12). *If*$\overline{S}^{\mathrm{E}}_{0}\geq0$*and*$\overline{S}^{\mathrm{I}}_{0}\geq0$, *then*$\overline{S}^{\mathrm{E}}(t)\geq0$*and*$\overline{S}^{\mathrm{I}}(t)\geq0$*for all*$t\geq0$.

### Proposition 2

*Consider the two*-*class mean excitatory and mean inhibitory synaptic drive network given by* (11) *and* (12), *and let*$\mathcal{M}\triangleq\{(\overline {S}^{\mathrm{E}}, \overline{S}^{\mathrm{I}})\in\mathbb{R}^{2}: 0\leq\overline {S}^{\mathrm{E}}\leq f_{\max}\lambda^{\mathrm{E}}\textit{ and }0\leq\overline {S}^{\mathrm{I}}\leq f_{\max}\lambda^{\mathrm{I}}\}$. *Then*$\mathcal{M}$*is positively invariant with respect to* (11) *and* (12).

### Proof

Let $[1\ 0]^{\mathrm{T}}$ be a normal vector to the line $\overline{S}^{\mathrm{E}}=0$ directed toward the region $\mathcal{M}$ and note that, since $f(x)\geq0$, $x\in\mathbb{R}$,

$$ \begin{bmatrix} 1 & 0 \end{bmatrix} \begin{bmatrix} \overline{f}_{1}\\ \overline{f}_{2} \end{bmatrix} =\overline{f}_{1}=f\bigl(a \overline{S}^{\mathrm{E}}-b\overline{S}^{\mathrm{I}}+v_{\mathrm{th}}^{\mathrm{E}} \bigr)-\frac{1}{\lambda^{\mathrm{E}}}\overline{S}^{\mathrm{E}}\geq0,\quad \overline{S}^{\mathrm{E}}=0. $$

Hence, the vector field $\overline{f}\triangleq[\overline{f}_{1}\ \overline{f}_{2}]^{\mathrm{T}}$ along the line $\overline{S}^{\mathrm{E}}=0$ is directed toward the region $\mathcal{M}$. Next, let $[-1\ 0]^{\mathrm{T}}$ be a normal vector to the line $\overline{S}^{\mathrm{E}}=f_{\mathrm{max}}\lambda ^{\mathrm{E}}$ directed toward the region $\mathcal{M}$ and note that, since $f(x)\leq f_{\max}$, $x\in\mathbb{R}$,

$$\begin{aligned} \begin{bmatrix} -1 & 0 \end{bmatrix} \begin{bmatrix} \overline{f}_{1}\\ \overline{f}_{2} \end{bmatrix} =-\overline{f}_{1}=-f\bigl(a \overline{S}^{\mathrm{E}}-b\overline{S}^{\mathrm{I}}+v_{\mathrm{th}}^{\mathrm{E}} \bigr)+\frac{1}{\lambda^{\mathrm{E}}}\overline{S}^{\mathrm{E}}\geq0,\quad \overline{S}^{\mathrm{E}}=f_{\max}\lambda^{\mathrm{E}}. \end{aligned}$$

Hence, the vector field *f̅* along the line $\overline {S}^{\mathrm{E}}=f_{\max}\lambda^{\mathrm{E}}$ is directed inward toward the region $\mathcal{M}$.

Alternatively, let $[0\ 1]^{\mathrm{T}}$ be a normal vector along the line $\overline{S}^{\mathrm{I}}=0$ directed toward the region $\mathcal{M}$ and note that, since $f(x)\geq0$, $x\in\mathbb{R}$,

$$ \begin{bmatrix} 0 & 1 \end{bmatrix} \begin{bmatrix} \overline{f}_{1}\\ \overline{f}_{2} \end{bmatrix} =\overline{f}_{2}=f\bigl(c \overline{S}^{\mathrm{E}}-d\overline{S}^{\mathrm{I}}+v_{\mathrm{th}}^{\mathrm{I}} \bigr)-\frac{1}{\lambda^{\mathrm{I}}}\overline{S}^{\mathrm{I}} \geq0,\quad \overline{S}^{\mathrm{I}}=0. $$

Hence, the vector field *f̅* along the line $\overline {S}^{\mathrm{I}}=0$ is directed inward toward the region $\mathcal{M}$. Finally, let $[0\ {-}1]^{\mathrm{T}}$ be a normal vector along the line $\overline{S}^{\mathrm{I}}=f_{\max}\lambda^{\mathrm{I}}$ directed toward the region $\mathcal{M}$ and note that, since $f(x)\leq f_{\max}$, $x\in \mathbb{R}$,

$$ \begin{bmatrix} 0 & {-}1 \end{bmatrix} \begin{bmatrix} \overline{f}_{1}\\ \overline{f}_{2} \end{bmatrix} =-\overline{f}_{2}=-f\bigl(c \overline{S}^{\mathrm{E}}-d\overline{S}^{\mathrm{I}}+v_{\mathrm{th}}^{\mathrm{I}} \bigr)+\frac{1}{\lambda^{\mathrm{I}}}\overline{S}^{\mathrm{I}} \geq0,\quad \overline{S}^{\mathrm{I}}=f_{\max}\lambda^{\mathrm{I}}. $$

Hence, the vector field *f̅* along the line $\overline {S}^{\mathrm{I}}=f_{\max}\lambda^{\mathrm{I}}$ is directed inward toward the region $\mathcal{M}$. Thus, along the boundary $\partial\mathcal{M}$ of $\mathcal{M}$ the vector field *f̅* is directed inward toward the region $\mathcal{M}$, and hence, $\mathcal{M}$ is positively invariant with respect to (11) and (12). □

**Fig. 1:**
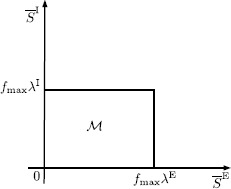
Visualization of the region $\mathcal{M}$

A visualization of the region $\mathcal{M}$ is shown in Fig. 1.

Note that the equilibrium points $(\overline{S}^{\mathrm{E}}_{\mathrm{e}},\overline{S}^{\mathrm{I}}_{\mathrm{e}})$ of (11) and (12) are characterized by the solution to

15 $$\begin{aligned} f\bigl(a\overline{S}^{\mathrm{E}}-b\overline{S}^{\mathrm{I}}+v_{\mathrm{th}}^{\mathrm{E}} \bigr)&=\frac{1}{\lambda^{\mathrm{E}}}\overline{S}^{\mathrm{E}}, \end{aligned}$$

16 $$\begin{aligned} f\bigl(c\overline{S}^{\mathrm{E}}-d\overline{S}^{\mathrm{I}}+v_{\mathrm{th}}^{\mathrm{I}} \bigr)&=\frac{1}{\lambda^{\mathrm{I}}}\overline{S}^{\mathrm{I}}. \end{aligned}$$

Since $f(\cdot)$ is increasing, $f(\cdot)$ is invertible. Hence, it follows from (15) and (16) that

17 $$\begin{aligned} \overline{S}^{\mathrm{I}}&=\frac{1}{b} \biggl(a \overline{S}^{\mathrm{E}}+v_{\mathrm{th}}^{\mathrm{E}}-f^{-1} \biggl(\frac{\overline{S}^{\mathrm{E}}}{\lambda^{\mathrm{E}}} \biggr) \biggr)\triangleq g\bigl(\overline{S}^{\mathrm{E}} \bigr), \end{aligned}$$

18 $$\begin{aligned} \overline{S}^{\mathrm{E}}&=\frac{1}{c} \biggl(d \overline{S}^{\mathrm{I}}-v_{\mathrm{th}}^{\mathrm{I}}+f^{-1} \biggl(\frac{\overline{S}^{\mathrm{I}}}{\lambda^{\mathrm{I}}} \biggr) \biggr)\triangleq h\bigl(\overline{S}^{\mathrm{I}} \bigr), \end{aligned}$$

where $f^{-1}(x)=\frac{1}{\gamma}\log_{e}{\frac{x}{f_{\max}-x}}$. Now, it follows from (17) and (18) that

19 $$\begin{aligned} \frac{\mathrm{d}g(\overline{S}^{\mathrm{E}})}{\mathrm{d}\overline{S}^{\mathrm{E}}}={}&\frac{1}{b} \biggl(a- \frac{f_{\max}\lambda^{\mathrm{E}}}{\gamma\overline {S}^{\mathrm{E}}(f_{\max}\lambda^{\mathrm{E}}-\overline{S}^{\mathrm{E}})} \biggr) \\ ={}&\frac{a}{b \overline{S}^{\mathrm{E}}(f_{\max}\lambda^{\mathrm{E}}-\overline {S}^{\mathrm{E}})} \biggl(- \bigl(\overline{S}^{\mathrm{E}} \bigr)^{2}+f_{\max}\lambda ^{\mathrm{E}} \overline{S}^{\mathrm{E}}-\frac{f_{\max}\lambda^{\mathrm{E}}}{a\gamma } \biggr) \\ ={}&\frac{a}{b \overline{S}^{\mathrm{E}}(f_{\max}\lambda^{\mathrm{E}}-\overline {S}^{\mathrm{E}})} \biggl(- \biggl(\overline{S}^{\mathrm{E}}- \frac{f_{\max}\lambda ^{\mathrm{E}}}{2} \biggr)^{2}+ \biggl(\frac{f_{\max}\lambda^{\mathrm{E}}}{2} \biggr)^{2} \\ &{}-\frac{f_{\max}\lambda^{\mathrm{E}}}{a\gamma} \biggr), \end{aligned}$$

20 $$\begin{aligned} \frac{\mathrm{d}h(\overline{S}^{\mathrm{I}})}{\mathrm{d}\overline{S}^{\mathrm{I}}}={}&\frac{1}{c} \biggl(d+ \frac{f_{\max}\lambda^{\mathrm{I}}}{\gamma\overline {S}^{\mathrm{I}}(f_{\max}\lambda^{\mathrm{I}}-\overline{S}^{\mathrm{I}})} \biggr)>0. \end{aligned}$$

Next, since *h* is increasing, $h^{-1}$ exists and is an increasing function. Furthermore, it follows from (18) that $\overline {S}^{\mathrm{I}}=h^{-1}(\overline{S}^{\mathrm{E}})$. In addition, note that $f'(x)=\gamma f_{\max}\frac{e^{\gamma x}}{1+e^{\gamma x}}\frac {1}{1+e^{\gamma x}}=\gamma f(x) (1-\frac{f(x)}{f_{\max}} )$. Now, using (15) and (16) it follows from (11) and (12) that the Jacobian matrix of the system vector field evaluated at the equilibrium point $(\overline{S}^{\mathrm{E}}_{\mathrm{e}},\overline{S}^{\mathrm{I}}_{\mathrm{e}})$ is given by

21 $$\begin{aligned} J\bigl(\overline{S}^{\mathrm{E}}_{\mathrm{e}},\overline{S}^{\mathrm{I}}_{\mathrm{e}} \bigr) &\triangleq \begin{bmatrix} J_{11}(\overline{S}^{\mathrm{E}}_{\mathrm{e}},\overline{S}^{\mathrm{I}}_{\mathrm{e}}) & J_{12}(\overline{S}^{\mathrm{E}}_{\mathrm{e}},\overline{S}^{\mathrm{I}}_{\mathrm{e}}) \\ J_{21}(\overline{S}^{\mathrm{E}}_{\mathrm{e}},\overline{S}^{\mathrm{I}}_{\mathrm{e}}) & J_{22}(\overline{S}^{\mathrm{E}}_{\mathrm{e}},\overline{S}^{\mathrm{I}}_{\mathrm{e}}) \end{bmatrix} \\ &= \begin{bmatrix} -\frac{1}{\lambda^{\mathrm{E}}}+a\gamma\frac{\overline{S}^{\mathrm{E}}_{\mathrm{e}}}{\lambda^{\mathrm{E}}} (1-\frac{\overline{S}^{\mathrm{E}}_{\mathrm{e}}}{f_{\max }\lambda^{\mathrm{E}}} ) &-b\gamma\frac{\overline{S}^{\mathrm{E}}_{\mathrm{e}}}{\lambda^{\mathrm{E}}} (1-\frac{\overline{S}^{\mathrm{E}}_{\mathrm{e}}}{f_{\max }\lambda^{\mathrm{E}}} )\\ c\gamma\frac{\overline{S}^{\mathrm{I}}_{\mathrm{e}}}{\lambda^{\mathrm{I}}} (1-\frac{\overline{S}^{\mathrm{I}}_{\mathrm{e}}}{f_{\max}\lambda^{\mathrm{I}}} ) & -\frac{1}{\lambda^{\mathrm{I}}}-d\gamma\frac{\overline{S}^{\mathrm{I}}_{\mathrm{e}}}{\lambda^{\mathrm{I}}} (1-\frac{\overline{S}^{\mathrm{I}}_{\mathrm{e}}}{f_{\max }\lambda^{\mathrm{I}}} ) \end{bmatrix} . \end{aligned}$$

Finally, note that if $a>\frac{4}{\gamma f_{\max}\lambda^{\mathrm{E}}}$, then it follows from (19) that $g(\overline{S}^{\mathrm{E}})$ has a maximum and a minimum value at $\overline{S}^{\mathrm{E}}_{\mathrm{max}}$ and $\overline{S}^{\mathrm{E}}_{\mathrm{min}}$ given by, respectively,

22 $$\begin{aligned} \overline{S}^{\mathrm{E}}_{\max}=\frac{f_{\max}\lambda^{\mathrm{E}}+\sqrt{(f_{\max }\lambda^{\mathrm{E}})^{2}-\frac{4f_{\max}\lambda^{\mathrm{E}}}{a\gamma}}}{2}, \end{aligned}$$

23 $$\begin{aligned} \overline{S}^{\mathrm{E}}_{\min}=\frac{f_{\max}\lambda^{\mathrm{E}}-\sqrt{(f_{\max }\lambda^{\mathrm{E}})^{2}-\frac{4f_{\max}\lambda^{\mathrm{E}}}{a\gamma}}}{2}. \end{aligned}$$

### Proposition 3

*Consider the two*-*class mean excitatory and mean inhibitory synaptic drive network given by* (11) *and* (12). *If*$a>\frac{4}{\gamma f_{\max}\lambda^{\mathrm{E}}}$, $\frac{4}{c\gamma f_{\max}\lambda^{\mathrm{I}}}<\frac{b\gamma f_{\max }\lambda^{\mathrm{E}}}{a\gamma f_{\max}\lambda^{\mathrm{E}}-4}-\frac{d}{c}$, *and*

$$\begin{aligned} h^{-1}\bigl(\overline{S}^{\mathrm{E}}_{\max} \bigr)-h^{-1}\bigl(\overline{S}^{\mathrm{E}}_{\min } \bigr)>{}&\frac{1}{b} \biggl[a\sqrt{\bigl(f_{\max} \lambda^{\mathrm{E}}\bigr)^{2}-\frac{4 f_{\max }\lambda^{\mathrm{E}}}{a\gamma}} \\ &{}-\frac{2}{\gamma}\log_{e}\frac{a\gamma (f_{\max}\lambda^{\mathrm{E}}+\sqrt{(f_{\max}\lambda^{\mathrm{E}})^{2}-\frac{4 f_{\max}\lambda^{\mathrm{E}}}{a\gamma}} )^{2}}{4 f_{\max}\lambda^{\mathrm{E}}} \biggr], \end{aligned} $$

*then there exist input voltages*$v_{\mathrm{th}}^{\mathrm{I}}$*and*$v_{\mathrm{th}}^{\mathrm{E}}$*such that* (11) *and* (12) *have exactly one equilibrium point*$(\overline {S}^{\mathrm{E}}_{\mathrm{e}},\overline{S}^{\mathrm{I}}_{\mathrm{e}})$. *Moreover*, $\operatorname {det}J(\overline{S}^{\mathrm{E}}_{\mathrm{e}},\overline{S}^{\mathrm{I}}_{\mathrm{e}})>0$.

### Proof

**Fig. 2:**
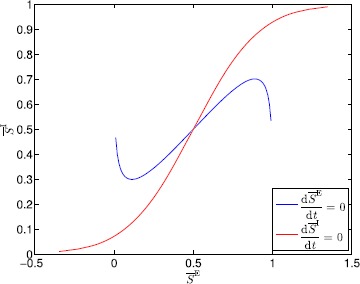
Plots of $\frac{\mathrm{d}\overline{S}^{\mathrm{E}}}{\mathrm{d}t}=0$ and $\frac{\mathrm{d}\overline{S}^{\mathrm{I}}}{\mathrm{d}t}=0$ for $a=10$, $b=9$, $c=6$, $d=1$, $v_{\mathrm{th}}^{\mathrm{E}}=-0.5$, $v_{\mathrm{th}}^{\mathrm{I}}=-2.5$, $\lambda^{\mathrm{E}}=1$, $\lambda^{\mathrm{I}}=1$, $f_{\max}=1$, and $\gamma=1$

If $a>\frac{4}{\gamma f_{\max}\lambda^{\mathrm{E}}}$, $g(\overline{S}^{\mathrm{E}}_{\max})-g(\overline{S}^{\mathrm{E}}_{\min})< h^{-1}(\overline{S}^{\mathrm{E}}_{\max})-h^{-1}(\overline{S}^{\mathrm{E}}_{\min})$, and the maximum value of the gradient of $h^{-1}(\cdot)$ is greater than the maximum value of the gradient of $g(\cdot)$, then $\frac{\mathrm{d}\overline{S}^{\mathrm{E}}}{\mathrm{d}t}=0$ and $\frac{\mathrm{d}\overline{S}^{\mathrm{I}}}{\mathrm{d}t}=0$ can be shifted so that there exists exactly one intersection $(\overline {S}^{\mathrm{E}}_{\mathrm{e}},\overline{S}^{\mathrm{I}}_{\mathrm{e}})$ of $g(\cdot)$ and $h^{-1}(\cdot)$, and the gradient of $g(\cdot)$ at $(\overline{S}^{\mathrm{E}}_{\mathrm{e}},\overline{S}^{\mathrm{I}}_{\mathrm{e}})$ is less than the gradient of $h^{-1}(\cdot)$ at $(\overline {S}^{\mathrm{E}}_{\mathrm{e}},\overline{S}^{\mathrm{I}}_{\mathrm{e}})$ as shown in Fig. 2.

Note that the maximum values of the gradients of $g(\cdot)$ and $h^{-1}(\cdot)$ occur at the inflection points of $g(\cdot)$ and $h(\cdot)$, respectively, and from (19) and (20) the inflection points of $g(\cdot)$ and $h(\cdot)$ correspond to $\overline {S}^{\mathrm{E}}=\frac{f_{\max}\lambda^{\mathrm{E}}}{2}$ and $\overline{S}^{\mathrm{I}}=\frac{f_{\max}\lambda^{\mathrm{I}}}{2}$, respectively. Thus, the maximum values of the gradients of $g(\cdot)$ and $h^{-1}(\cdot)$ correspond to $\frac{1}{b} (a-\frac{4}{\gamma f_{\max}\lambda^{\mathrm{E}}} )$ and $\frac{1}{\frac{1}{c} (d+{4}/(\gamma f_{\max}\lambda^{\mathrm{I}}) )}$, respectively. Now, the condition that the maximum values of the gradient of $h^{-1}(\cdot)$ is greater than the maximum value of the gradient of $g(\cdot)$ implies that $\frac{1}{\frac{1}{c} (d+{4}/(\gamma f_{\max}\lambda^{\mathrm{I}}) )}>\frac{1}{b} (a-\frac{4}{\gamma f_{\max} \lambda^{\mathrm{E}}} )$, and hence, $\frac {4}{c\gamma f_{\max}\lambda^{\mathrm{I}}}<\frac{b\gamma f_{\max}\lambda^{\mathrm{E}}}{a\gamma\lambda^{\mathrm{E}}-4}-\frac{d}{c}$.

Next, the condition $g(\overline{S}^{\mathrm{E}}_{\max})-g(\overline{S}^{\mathrm{E}}_{\min})< h^{-1}(\overline{S}^{\mathrm{E}}_{\max})-h^{-1}(\overline{S}^{\mathrm{E}}_{\min})$ implies that

24 $$\begin{aligned} &h^{-1}\bigl(\overline{S}^{\mathrm{E}}_{\max} \bigr)-h^{-1}\bigl(\overline{S}^{\mathrm{E}}_{\min } \bigr) \\ &\quad >g\bigl(\overline{S}^{\mathrm{E}}_{\max}\bigr)-g\bigl( \overline{S}^{\mathrm{E}}_{\min }\bigr) \\ &\quad =\frac{1}{b} \biggl[a\bigl(\overline{S}^{\mathrm{E}}_{\max}- \overline{S}^{\mathrm{E}}_{\min}\bigr)-\frac{1}{\gamma} \log_{e}\frac{\overline{S}^{\mathrm{E}}_{\max }(f_{\max}\lambda^{\mathrm{E}}-\overline{S}^{\mathrm{E}}_{\min})}{\overline {S}^{\mathrm{E}}_{\min}(f_{\max}\lambda^{\mathrm{E}}-\overline{S}^{\mathrm{E}}_{\max })} \biggr] \\ &\quad =\frac{1}{b} \biggl[a\sqrt{\bigl(f_{\max}\lambda^{\mathrm{E}} \bigr)^{2}-\frac{4f_{\max }\lambda^{\mathrm{E}}}{a\gamma}}-\frac{2}{\gamma}\log_{e} \frac{\overline {S}^{\mathrm{E}}_{\max}}{\overline{S}^{\mathrm{E}}_{\min}} \biggr] \\ &\quad =\frac{1}{b} \biggl[a\sqrt{\bigl(f_{\max}\lambda^{\mathrm{E}} \bigr)^{2}-\frac{4f_{\max }\lambda^{\mathrm{E}}}{a\gamma}} \\ &\qquad {}-\frac{2}{\gamma}\log_{e}\frac{a\gamma (f_{\max}\lambda^{\mathrm{E}}+\sqrt{(f_{\max}\lambda^{\mathrm{E}})^{2}-\frac{4f_{\max}\lambda^{\mathrm{E}}}{a\gamma}} )^{2}}{4f_{\max}\lambda^{\mathrm{E}}} \biggr]. \end{aligned}$$

Hence, there exist input voltages $v_{\mathrm{th}}^{\mathrm{I}}$ and $v_{\mathrm{th}}^{\mathrm{E}}$ such that (11) and (12) have exactly one equilibrium point $(\overline {S}^{\mathrm{E}}_{\mathrm{e}},\overline{S}^{\mathrm{I}}_{\mathrm{e}})$.

Finally, note that since the gradient of $g(\cdot)$ at $(\overline {S}^{\mathrm{E}}_{\mathrm{e}},\overline{S}^{\mathrm{I}}_{\mathrm{e}})$ is less than the gradient of $h^{-1}(\cdot)$ at $(\overline{S}^{\mathrm{E}}_{\mathrm{e}},\overline {S}^{\mathrm{I}}_{\mathrm{e}})$, it follows from (19), (20), and (21) that $-\frac{J_{11}(\overline{S}^{\mathrm{E}}_{\mathrm{e}})}{J_{12}(\overline{S}^{\mathrm{E}}_{\mathrm{e}})}<-\frac{J_{21}(\overline {S}^{\mathrm{I}}_{\mathrm{e}})}{J_{22}(\overline{S}^{\mathrm{I}}_{\mathrm{e}})}$, and hence, $\operatorname {det}J(\overline{S}^{\mathrm{E}}_{\mathrm{e}},\overline{S}^{\mathrm{I}}_{\mathrm{e}})>0$. □

### Theorem 1

*Consider the two*-*class mean excitatory and mean inhibitory synaptic drive network given by* (11) *and* (12), *and assume that the hypothesis in Proposition *3*is satisfied*. *If*$\operatorname {tr}J(\overline {S}^{\mathrm{E}}_{\mathrm{e}},\overline{S}^{\mathrm{I}}_{\mathrm{e}})<0$, *then there exist input voltages*$v_{\mathrm{th}}^{\mathrm{I}}$*and*$v_{\mathrm{th}}^{\mathrm{E}}$*such that* (11) *and* (12) *have exactly one equilibrium point and this equilibrium point is asymptotically stable*. *Alternatively*, *if*$\operatorname {tr}J(\overline{S}^{\mathrm{E}}_{\mathrm{e}},\overline{S}^{\mathrm{I}}_{\mathrm{e}})>0$, *then there exist input voltages*$v_{\mathrm{th}}^{\mathrm{I}}$*and*$v_{\mathrm{th}}^{\mathrm{E}}$*such that* (11) *and* (12) *possess a limit cycle*. *Moreover*, *the limit cycle is stable*.

### Proof

It follows from Proposition 3 that (11) and (12) have exactly one equilibrium point $(\overline{S}^{\mathrm{E}}_{\mathrm{e}},\overline {S}^{\mathrm{I}}_{\mathrm{e}})$ and $\operatorname {det}J(\overline{S}^{\mathrm{E}}_{\mathrm{e}},\overline{S}^{\mathrm{I}}_{\mathrm{e}})>0$. Hence, if $\operatorname {tr}J(\overline {S}^{\mathrm{E}}_{\mathrm{e}},\overline{S}^{\mathrm{I}}_{\mathrm{e}})<0$, then $(\overline {S}^{\mathrm{E}}_{\mathrm{e}},\overline{S}^{\mathrm{I}}_{\mathrm{e}})$ is an asymptotically stable equilibrium point.

Alternatively, if $\operatorname {tr}J(\overline{S}^{\mathrm{E}}_{\mathrm{e}},\overline {S}^{\mathrm{I}}_{\mathrm{e}})>0$, then $(\overline{S}^{\mathrm{E}}_{\mathrm{e}},\overline {S}^{\mathrm{I}}_{\mathrm{e}})$ is an unstable equilibrium point. In this case, define $\mathcal{R}\triangleq\mathcal{M}\setminus{(\overline{S}^{\mathrm{E}}_{\mathrm{e}},\overline{S}^{\mathrm{I}}_{\mathrm{e}})}$ and note that since $\mathcal {M}$ is positively invariant with respect to (11) and (12), and $(\overline{S}^{\mathrm{E}}_{\mathrm{e}},\overline{S}^{\mathrm{I}}_{\mathrm{e}})$ is an unstable equilibrium point, it follows that $\mathcal{R}$ is positively invariant with respect to (11) and (12). Hence, it follows from the Poincaré–Bendixson theorem ([23], p. 109) that $\mathcal {R}$ contains a periodic orbit. Furthermore, since $\mathcal{R}$ is positively invariant and the equilibrium point $(\overline{S}^{\mathrm{E}}_{\mathrm{e}},\overline{S}^{\mathrm{I}}_{\mathrm{e}})$ is unstable, the limit cycle is stable. □

## Large Neural Populations, Synchronization, and Partial State Equipartitioning

One of the most important questions in neuroscience is how do neurons, or collections of neurons, communicate. There is extensive experimental verification that collections of neurons may function as oscillators and the synchronization of oscillators may play a key role in the transmission of information within the central nervous system [24–26]. This may be particularly relevant to understanding the mechanism of action for general anesthesia.

It has been known for a long time that general anesthesia has profound effects on the spectrum of oscillations in the electroencephalograph (EEG) [15, 27, 28]. More recently, the authors in [19] have suggested that thalamocortical circuits function as neural pacemakers and that alterations in the thalamic oscillations are associated with the induction of general anesthesia. Furthermore, it is well known that anesthetic drugs frequently induce epileptiform activity as part of the progression to the state of unconsciousness [29]. Multiple lines of evidence indicate that anesthetic agents impact neural oscillators. In addition, epileptiform activity implies synchronization of oscillators. This leads to the possibility that synchronization of these oscillators is involved in the transition to the anesthetic state.

Sufficient conditions for synchronization and full state equipartition for an excitatory and inhibitory cortical neural network are given in [7]. It has been observed, however, that when patients lose consciousness there are differences in neural activity in different anatomic regions of the cerebral cortex. This can be captured by biological neural network models that exhibit *partial synchronization*, wherein part of the system’s state (neural activity) is synchronized and the other parts fire at normal levels.

Before proceeding, it is important to distinguish between the notions of synchronization and state equipartitioning or *consensus*. System synchronization refers to the fact that the dynamical system states achieve *temporal coincidence* over a finite or infinite time horizon, whereas state equipartitioning refers to the fact that the dynamical system states *converge* to a common value over a finite or infinite time horizon. Hence, both notions involve state agreement in some sense. However, equipartitioning involves convergence of the state values to a constant state, whereas synchronization involves agreement over time instants. Thus, equipartitioning implies synchronization; however, the converse is not necessarily true. It is only true in so far as consensus is interpreted to hold over time instants.

In order to address partial synchronization, we make a high-level assumption regarding neuronal connectivity by postulating the existence of a subset of inhibitory neurons that themselves do not receive inhibitory input. It is important to note that the existence of such a subset has not been demonstrated experimentally. However, it is not biologically implausible and, as we shall demonstrate, this assumption leads to sufficient conditions for partial synchronization.

To address the problem of partial synchronization, let $S^{\mathrm{E}}(t)\triangleq[S^{\mathrm{E}}_{1}(t),\ldots, S^{\mathrm{E}}_{n_{\mathrm{E}}}(t)] \in \mathbb{R}^{n_{\mathrm{E}}}$, $S^{\mathrm{I}}(t)\triangleq[S^{\mathrm{I}}_{1}(t),\ldots,S^{\mathrm{I}}_{n_{\mathrm{I}}}(t)]\in\mathbb{R}^{n_{\mathrm{I}}}$, $\tau_{i}^{\mathrm{E}}=\lambda_{i}^{\mathrm{E}}$, $i=1,\ldots,n_{\mathrm{E}}$, $\tau_{i}^{\mathrm{I}}=\lambda _{i}^{\mathrm{I}}$, $i=1,\ldots,n_{\mathrm{I}}$, and assume $B_{i}^{\mathrm{E}}=B_{i}^{\mathrm{I}}=1$ so that the vector–matrix form of (6) and (7) can be written as

25 $$\begin{aligned} \dot{S}^{\mathrm{E}}(t)={}&{-}L^{\mathrm{E}}S^{\mathrm{E}}(t)+f^{\mathrm{E}} \bigl(A^{\mathrm{EE}}S^{\mathrm{E}}(t)+A^{\mathrm{EI}}S^{\mathrm{I}}(t)+ \tilde{v}_{\mathrm{th}}^{\mathrm{E}}(t)\bigr), \\ &{}\qquad\qquad\qquad\qquad\qquad\qquad\qquad\qquad\qquad S^{\mathrm{E}}(0)=S^{\mathrm{E}}_{0}, \quad t\geq0, \end{aligned}$$

26 $$\begin{aligned} \dot{S}^{\mathrm{I}}(t)={}&{-}L^{\mathrm{I}}S^{\mathrm{I}}(t)+f^{\mathrm{I}} \bigl(A^{\mathrm{IE}}S^{\mathrm{E}}(t)+A^{\mathrm{II}}S^{\mathrm{I}}(t)+ \tilde{v}_{\mathrm{th}}^{\mathrm{I}}(t)\bigr), \\ &{}\qquad\qquad\qquad\qquad\qquad\qquad\qquad\qquad\qquad\qquad\qquad S^{\mathrm{I}}(0)=S^{\mathrm{I}}_{0}, \end{aligned}$$

where $L^{\mathrm{E}}\triangleq \operatorname {diag}[\frac{1}{\tau_{1}^{\mathrm{E}}},\ldots,\frac{1}{\tau_{n_{\mathrm{E}}}^{\mathrm{E}}} ]\in\mathbb {R}^{{n_{\mathrm{E}}}\times{n_{\mathrm{E}}}}$ and $L^{\mathrm{I}}\triangleq \operatorname {diag}[\frac{1}{\tau_{1}^{\mathrm{I}}},\ldots,\frac{1}{\tau_{n_{\mathrm{I}}}^{\mathrm{I}}} ]\in\mathbb{R}^{{n_{\mathrm{I}}}\times{n_{\mathrm{I}}}}$ are time constant matrices, $A^{\mathrm{EE}}\triangleq[A^{\mathrm{EE}}_{ij}]\in\mathbb {R}^{n_{\mathrm{E}}\times n_{\mathrm{E}}}$, $A^{\mathrm{EI}}\triangleq[A^{\mathrm{EI}}_{ij}]\in\mathbb{R}^{n_{\mathrm{E}}\times n_{\mathrm{I}}}$, $A^{\mathrm{IE}}\triangleq[A^{\mathrm{IE}}_{ij}]\in\mathbb{R}^{n_{\mathrm{I}}\times n_{\mathrm{E}}}$, $A^{\mathrm{II}}\triangleq[A^{\mathrm{II}}_{ij}]\in\mathbb{R}^{n_{\mathrm{I}}\times n_{\mathrm{I}}}$ are matrices representing the strengths of the synaptic interconnections, $\tilde{v}_{\mathrm{th}}^{\mathrm{E}}\triangleq [v_{\mathrm{th}1}^{\mathrm{E}},\ldots,v_{\mathrm{th}n_{\mathrm{E}}}^{\mathrm{E}}]^{\mathrm{T}}$, $\tilde{v}_{\mathrm{th}}^{\mathrm{I}}\triangleq[v_{\mathrm{th}1}^{\mathrm{I}},\ldots ,v_{\mathrm{th}n_{\mathrm{I}}}^{\mathrm{I}}]^{\mathrm{T}}$, and

27 $$\begin{aligned} f^{\mathrm{E}}\bigl(A^{\mathrm{EE}}S^{\mathrm{E}}+A^{\mathrm{EI}}S^{\mathrm{I}}+ \tilde{v}_{\mathrm{th}}^{\mathrm{E}}\bigr)\triangleq {}&\Biggl[f_{1} \Biggl(\sum_{j=1}^{n_{\mathrm{E}}}A_{1j}^{\mathrm{EE}}S_{j}^{\mathrm{E}}+ \sum_{j'=1}^{n_{\mathrm{I}}}A_{1j'}^{\mathrm{EI}}S_{j'}^{\mathrm{I}}+v_{\mathrm{th}1}^{\mathrm{E}} \Biggr),\ldots, \\ & {}f_{n_{\mathrm{E}}} \Biggl(\sum_{j=1}^{n_{\mathrm{E}}}A_{{n_{\mathrm{E}}}j}^{\mathrm{EE}}S_{j}^{\mathrm{E}}+ \sum_{j'=1}^{n_{\mathrm{I}}}A_{{n_{\mathrm{E}}}j'}^{\mathrm{EI}}S_{j'}^{\mathrm{I}}+v_{\mathrm{th}{n_{\mathrm{E}}}}^{\mathrm{E}} \Biggr) \Biggr]^{\mathrm{T}}, \end{aligned}$$

28 $$\begin{aligned} f^{\mathrm{I}}\bigl(A^{\mathrm{IE}}S^{\mathrm{E}}+A^{\mathrm{II}}S^{\mathrm{I}}+ \tilde{v}_{\mathrm{th}}^{\mathrm{I}}\bigr)\triangleq {}&\Biggl[f_{1} \Biggl(\sum_{j=1}^{n_{\mathrm{E}}}A_{1j}^{\mathrm{IE}}S_{j}^{\mathrm{E}}+ \sum_{j'=1}^{n_{\mathrm{I}}}A_{1j'}^{\mathrm{II}}S_{j'}^{\mathrm{I}}+v_{\mathrm{th}1}^{\mathrm{I}} \Biggr),\ldots, \\ &{} f_{n_{\mathrm{I}}} \Biggl(\sum_{j=1}^{n_{\mathrm{E}}}A_{{n_{\mathrm{I}}}j}^{\mathrm{IE}}S_{j}^{\mathrm{E}}+ \sum_{j'=1}^{n_{\mathrm{I}}}A_{{n_{\mathrm{I}}}j'}^{\mathrm{II}}S_{j'}^{\mathrm{I}}+v_{\mathrm{th}{n_{\mathrm{I}}}}^{\mathrm{I}} \Biggr) \Biggr]^{\mathrm{T}}, \end{aligned}$$

denote the vector activation functions describing the relationship between the synaptic drives and the firing rates of neurons with $f_{i}(x)$, $x\in\mathbb{R}$, $i=1,\ldots,n_{\mathrm{E}}$, and $f_{k}(x)$, $x\in \mathbb{R}$, $k=1,\ldots,n_{\mathrm{I}}$, defined as in (9).

Next, define $S^{\mathrm{I}}(t)\triangleq[(\tilde{S}_{1}^{\mathrm{I}}(t))^{\mathrm{T}},(\tilde{S}_{2}^{\mathrm{I}}(t))^{\mathrm{T}}]^{\mathrm{T}}$, where $\tilde{S}_{1}^{\mathrm{I}}(t)\triangleq[S_{1}^{\mathrm{I}}(t),\ldots,S^{\mathrm{I}}_{q}(t)]^{\mathrm{T}}$ denotes the vector of inhibitory synaptic drives that are coupled to both excitatory and inhibitory neurons and $\tilde{S}_{2}^{\mathrm{I}}(t)\triangleq[S_{q+1}^{\mathrm{I}}(t),\ldots,S^{\mathrm{I}}_{n_{\mathrm{I}}}(t)]^{\mathrm{T}}$ denotes the vector of inhibitory synaptic drives that are only coupled to excitatory neurons. In this case, the neuronal connectivity matrix *A* can be partitioned as

29 $$ A\triangleq \begin{bmatrix} A^{\mathrm{EE}} & A^{\mathrm{EI}}\\ A^{\mathrm{IE}} & A^{\mathrm{II}} \end{bmatrix} = \begin{bmatrix} A^{\mathrm{EE}} & A^{\mathrm{EI}}_{1} &A^{\mathrm{EI}}_{2}\\ A^{\mathrm{IE}}_{1} & A^{\mathrm{II}}_{1} & A^{\mathrm{II}}_{2}\\ A^{\mathrm{IE}}_{2} &0_{(n_{\mathrm{I}}-q)\times q} & 0_{(n_{\mathrm{I}}-q)\times (n_{\mathrm{I}}-q)} \end{bmatrix} , $$

where

$$\begin{aligned} A^{\mathrm{EI}}= \begin{bmatrix} A^{\mathrm{EI}}_{1}& A^{\mathrm{EI}}_{2}\end{bmatrix}, \qquad A^{\mathrm{IE}}= \begin{bmatrix} A^{\mathrm{IE}}_{1}\\ A^{\mathrm{IE}}_{2} \end{bmatrix} ,\qquad A^{\mathrm{II}}= \begin{bmatrix} A^{\mathrm{II}}_{1} & A^{\mathrm{II}}_{2}\\ 0_{(n_{\mathrm{I}}-q)\times q} & 0_{(n_{\mathrm{I}}-q)\times(n_{\mathrm{I}}-q)} \end{bmatrix} , \end{aligned}$$

$A^{\mathrm{EI}}_{1}\in\mathbb{R}^{n_{\mathrm{E}}\times q}$, $A^{\mathrm{EI}}_{2}\in\mathbb {R}^{n_{\mathrm{E}}\times(n_{\mathrm{I}}-q)}$, $A^{\mathrm{IE}}_{1}\in\mathbb {R}^{q\times n_{\mathrm{E}}}$, $A^{\mathrm{II}}_{1}\in\mathbb{R}^{q\times q}$, $A^{\mathrm{II}}_{2}\in\mathbb{R}^{q\times(n_{\mathrm{I}}-q)}$, and $A^{\mathrm{IE}}_{2}\in\mathbb{R}^{(n_{\mathrm{I}}-q)\times n_{\mathrm{E}}} $.

Now, (25) and (26) can be written as

30 $$\begin{aligned} \dot{S}^{\mathrm{E}}(t)={}&{-}L^{\mathrm{E}}S^{\mathrm{E}}(t)+f^{\mathrm{E}} \bigl(A^{\mathrm{EE}}S^{\mathrm{E}}(t)+A^{\mathrm{EI}}_{1} \tilde{S}_{1}^{\mathrm{I}}(t)+A^{\mathrm{EI}}_{2} \tilde{S}_{2}^{\mathrm{I}}(t)+\tilde{v}_{\mathrm{th}}^{\mathrm{E}}(t) \bigr), \\ &{}\quad\qquad\qquad\qquad\qquad\qquad\qquad\qquad\qquad S^{\mathrm{E}}(0)=S^{\mathrm{E}}_{0},\quad t\geq0, \end{aligned}$$

31 $$\begin{aligned} \dot{\tilde{S}}_{1}^{\mathrm{I}}(t)={}&{-}L_{1}^{\mathrm{I}} \tilde{S}_{1}^{\mathrm{I}}(t)+\tilde {f}^{\mathrm{I}}_{1} \bigl(A_{1}^{\mathrm{IE}}S^{\mathrm{E}}(t)+A_{1}^{\mathrm{II}} \tilde{S}_{1}^{\mathrm{I}}(t)+A_{2}^{\mathrm{II}} \tilde{S}_{2}^{\mathrm{I}}(t)+\tilde{v}^{\mathrm{I}}_{\mathrm{th1}}(t) \bigr), \\ & {}\qquad\qquad\qquad\qquad\qquad\qquad\qquad\qquad\qquad\qquad \tilde{S}_{1}^{\mathrm{I}}(0)=\tilde{S}_{10}^{\mathrm{I}}, \end{aligned}$$

32 $$\begin{aligned} \dot{\tilde{S}}_{2}^{\mathrm{I}}(t)={}&{-}L_{2}^{\mathrm{I}} \tilde{S}_{2}^{\mathrm{I}}(t)+\tilde {f}^{\mathrm{I}}_{2} \bigl(A_{2}^{\mathrm{IE}}S^{\mathrm{E}}(t)+ \tilde{v}^{\mathrm{I}}_{\mathrm{th2}}(t)\bigr),\quad \tilde{S}_{2}^{\mathrm{I}}(0)= \tilde{S}_{20}^{\mathrm{I}}, \end{aligned}$$

where

33 $$ L^{\mathrm{I}}\triangleq \begin{bmatrix} L_{1}^{\mathrm{I}} & 0_{q \times(n_{\mathrm{I}}-q)}\\ 0_{(n_{\mathrm{I}}-q)\times q} & L_{2}^{\mathrm{I}} \end{bmatrix} , $$

$L_{1}^{\mathrm{I}}\triangleq \operatorname {diag}[\frac{1}{\tau_{1}^{\mathrm{I}}},\ldots ,\frac{1}{\tau_{q}^{\mathrm{I}}} ]\in\mathbb{R}^{q\times q}$, $L_{2}^{\mathrm{I}}\triangleq \operatorname {diag}[\frac{1}{\tau_{q+1}^{\mathrm{I}}},\ldots,\frac {1}{\tau_{n_{\mathrm{I}}}^{\mathrm{I}}} ]\in\mathbb{R}^{(n_{\mathrm{I}}-q)\times (n_{\mathrm{I}}-q)}$, $\tilde{v}_{\mathrm{th}1} ^{\mathrm{I}}\triangleq[v_{\mathrm{th}1}^{\mathrm{I}}, \ldots, v_{\mathrm{th}q}^{\mathrm{I}}]^{\mathrm{T}}\in\mathbb {R}^{q}$, $\tilde{v}_{\mathrm{th}2}^{\mathrm{I}} \triangleq[v_{\mathrm{th}(q+1)}^{\mathrm{I}}, \ldots, v_{\mathrm{th}n_{\mathrm{I}}}^{\mathrm{I}}]^{\mathrm{T}}\in \mathbb{R}^{n_{\mathrm{I}}-q}$, and $f^{\mathrm{I}}= [(\tilde{f}_{1}^{\mathrm{I}})^{\mathrm{T}} \ (\tilde{f}_{2}^{\mathrm{I}})^{\mathrm{T}}]^{\mathrm{T}}$. Next, letting $S(t)\triangleq[(S^{\mathrm{E}}(t))^{\mathrm{T}}, (\tilde{S}_{1}^{\mathrm{I}}(t))^{\mathrm{T}}]^{\mathrm{T}}\triangleq[S_{1}(t),\ldots,S_{n_{\mathrm{E}}+q}(t)]^{\mathrm{T}}$, $\tilde{f}\triangleq[(f^{\mathrm{E}})^{\mathrm{T}}\ (\tilde{f}_{1}^{\mathrm{I}})^{\mathrm{T}}]^{\mathrm{T}}$, and $L\triangleq \operatorname {block-diag}[L^{\mathrm{E}}\ L_{1}^{\mathrm{I}}]$, (30), (31), and (32) can be written as

34 $$\begin{aligned} \dot{S}(t)&=-LS(t)+\tilde{f}\bigl(\tilde{A}S(t)+\tilde{B} \tilde{S}_{2}^{\mathrm{I}}(t)+\tilde{v}_{\mathrm{th}}(t)\bigr),\quad S(0)=S_{0},\quad t\geq0, \end{aligned}$$

35 $$\begin{aligned} \dot{\tilde{S}}_{2}^{\mathrm{I}}(t)&=-L_{2}^{\mathrm{I}} \tilde{S}_{2}^{\mathrm{I}}(t)+\tilde {f}^{\mathrm{I}}_{2} \bigl(A_{2}^{\mathrm{IE}}S^{\mathrm{E}}(t)+ \tilde{v}^{\mathrm{I}}_{\mathrm{th2}}(t)\bigr),\quad \tilde{S}_{2}^{\mathrm{I}}(0)= \tilde{S}_{20}^{\mathrm{I}}, \end{aligned}$$

where $\tilde{A}\triangleq \left[{\scriptsize\begin{matrix}{} A^{\mathrm{EE}} &A_{1}^{\mathrm{EI}}\cr A_{1}^{\mathrm{IE}} & A_{1}^{\mathrm{II}} \end{matrix}}\right] \in\mathbb{R}^{(n_{\mathrm{E}}+q)\times(n_{\mathrm{E}}+q)}$, $\tilde{B}\triangleq \left[{\scriptsize\begin{matrix}{} A_{2}^{\mathrm{EI}}\cr A_{2}^{\mathrm{II}} \end{matrix}}\right] \in\mathbb{R}^{(n_{\mathrm{E}}+q)\times(n_{\mathrm{I}}-q)}$, and $\tilde{v}_{\mathrm{th}}\triangleq \left[{\scriptsize\begin{matrix}{} \tilde{v}_{\mathrm{th}}^{\mathrm{E}}\cr \tilde{v}_{\mathrm{th1}}^{\mathrm{I}} \end{matrix}}\right] \in\mathbb{R}^{n_{\mathrm{E}}+q}$.

The following proposition and definitions are needed for the statement of the main results of this section.

### Proposition 4

[7]

*Consider the excitatory–inhibitory network given by* (25) *and* (26) *with the vector activation functions defined by* (27) *and* (28), *where*$f_{i}(x)$, $x\in \mathbb{R}$, $i=1,\ldots,n_{\mathrm{E}}$, *and*$f_{k}(x)$, $x\in\mathbb{R}$, $k=1,\ldots,n_{\mathrm{I}}$, *are defined as in* (9). *If*$S^{\mathrm{E}}_{0}\geq\geq0$*and*$S^{\mathrm{I}}_{0}\geq\geq0$, *then*$S^{\mathrm{E}}(t)\geq\geq 0$*and*$S^{\mathrm{I}}(t)\geq\geq0$*for all*$t\geq0$.

### Definition 1

The biological neural network given by (34) and (35) is said to be *globally exponentially stable with respect toSuniformly in*$\tilde{S}_{20}^{\mathrm{I}}$ if there exist scalars $\alpha, \beta>0$ such that $\Vert S(t)\Vert \leq\alpha \Vert S_{0}\Vert e^{-\beta t}$, $t\geq0$, for all $S_{0}\in\overline{\mathbb{R}}^{n_{\mathrm{E}}+q}_{+}$ and $\tilde{S}^{\mathrm{I}}_{20}\in\overline{\mathbb{R}}^{n_{\mathrm{I}}-q}_{+}$.

### Definition 2

The biological neural network given by (34) and (35) is said to be *globally asymptotically partially synchronized* if

$$ \lim_{t\to\infty}\bigl\vert S_{i}(t)-S_{j}(t) \bigr\vert = 0, $$

for all $S_{0}\in\overline{\mathbb{R}}^{n_{\mathrm{E}}+q}_{+}$, $\tilde{S}^{\mathrm{I}}_{20}\in\overline{\mathbb{R}}^{n_{\mathrm{I}}-q}_{+}$, and $i=1,\ldots, n_{\mathrm{E}}+q$, $i\neq j$.

### Definition 3

The biological neural network given by (34) and (35) is said to be *globally exponentially partially synchronized* if there exist constants $p>0$ and $\rho>0$ such that

$$ \bigl\vert S_{i}(t)-S_{j}(t)\bigr\vert \leq\rho e^{-pt}\vert S_{i0}-S_{j0}\vert ,\quad t\geq0, $$

for all $S_{0}\in\overline{\mathbb{R}}^{n_{\mathrm{E}}+q}_{+}$, $\tilde{S}^{\mathrm{I}}_{20}\in\overline{\mathbb{R}}^{n_{\mathrm{I}}-q}_{+}$, and $i=1,\ldots, n_{\mathrm{E}}+q$, $i\neq j$.

### Remark 1

It follows from Definitions 1 and 3 that if the biological neural network given by (34) and (35) is globally exponentially stable with respect to *S* uniformly in $\tilde{S}_{20}^{\mathrm{I}}$, then (34) and (35) is globally exponentially partially synchronized.

For the statement of the next theorem, $\mathcal{N}(X)$ denotes the nullspace of the matrix *X*.

### Theorem 2

*Consider the excitatory–inhibitory network given by* (34) *and* (35) *with the vector activation functions defined by* (27) *and* (28). *If*$\tilde{v}_{\mathrm{th}}(t)\leq\leq-\tilde{B}w$, $t\geq0$, *where*$w\triangleq[\min\{S^{\mathrm{I}}_{q+1}(0), \tau_{q+1}^{\mathrm{I}}{\eta_{q+1}}\},\ldots, \min\{S^{\mathrm{I}}_{n_{\mathrm{I}}}(0), \tau_{n_{\mathrm{I}}}^{\mathrm{I}}{\eta_{n_{\mathrm{I}}}}\} ]^{\mathrm{T}}\in\mathbb{R}^{n_{\mathrm{I}}-q}$*and*$\eta_{i}\triangleq\min_{t\geq 0}v_{\mathrm{th}i}^{\mathrm{I}}(t)$, $i=q+1,\ldots,n_{\mathrm{I}}$, *and there exist positive*-*definite matricesP*, $Q\in\mathbb{R}^{(n_{\mathrm{E}}+q)\times (n_{\mathrm{E}}+q)}$*and a diagonal positive*-*definite matrix*$R\in\mathbb {R}^{(n_{\mathrm{E}}+q)\times(n_{\mathrm{E}}+q)}$*such that*

36 $$ \begin{bmatrix} Q & -P\\ -P & R \end{bmatrix} \geq0, $$

*and*$\varOmega>0$, *where*

37 $$ \varOmega\triangleq PL+LP-Q-\tilde{A}^{\mathrm{T}}R\tilde{A}, $$

*then* (34) *and* (35) *is globally exponentially partially synchronized*. *Alternatively*, *if*$\varOmega\geq0$*and*$\mathcal{N}(\varOmega)=\operatorname {span}(\mathbf{e}_{n_{\mathrm{E}}+q})$, *then* (34) *and* (35) *is globally asymptotically partially synchronized*.

### Proof

Consider the partial Lyapunov function candidate $V:\overline{\mathbb {R}}_{+}^{n_{\mathrm{E}}+q}\to\mathbb{R}$ given by $V(S)=S^{\mathrm{T}}PS$ and note that $\lambda_{\min}(P)\Vert S\Vert _{2}^{2}\leq V(S)\leq\lambda_{\max}(P)\Vert S\Vert _{2}^{2}$, $S\in\overline{\mathbb{R}}_{+}^{n_{\mathrm{E}}+q}$, where $\lambda_{\max }(P)$ and $\lambda_{\min}(P)$ denote the maximum and minimum eigenvalue of *P*, respectively, and $\Vert \cdot \Vert _{2}$ denotes Euclidean norm. It follows that the derivative of $V(S)$ along the trajectories of (34) is given by

38 $$\begin{aligned} \dot{V}\bigl(S,\tilde{S}_{2}^{\mathrm{I}} \bigr)={}&S^{\mathrm{T}}P\dot{S}+\dot{S}^{\mathrm{T}}PS \\ ={}&S^{\mathrm{T}}P\bigl(-LS+\tilde{f}\bigl(\tilde{A}S+\tilde{B} \tilde{S}_{2}^{\mathrm{I}}+\tilde{v}_{\mathrm{th}}\bigr)\bigr) \\ &{}+\bigl(-LS+\tilde{f}\bigl(\tilde{A}S+\tilde{B}\tilde{S}_{2}^{\mathrm{I}}+ \tilde {v}_{\mathrm{th}}\bigr)\bigr)^{\mathrm{T}}PS \\ ={}&{-}S^{\mathrm{T}}(PL+LP)S+2S^{\mathrm{T}}P\tilde{f}\bigl(\tilde{A}S+ \tilde{B}\tilde {S}_{2}^{\mathrm{I}}+\tilde{v}_{\mathrm{th}} \bigr). \end{aligned}$$

Next, it follows from (36) that

39 $$ \begin{aligned}[b] &\bigl[S^{\mathrm{T}} \tilde{f}^{\mathrm{T}}\bigl(\tilde{A}S+\tilde{B} \tilde{S}_{2}^{\mathrm{I}}+\tilde{v}_{\mathrm{th}}\bigr)\bigr] \begin{bmatrix} Q & -P\\ -P & R \end{bmatrix} \begin{bmatrix} S\\ \tilde{f}(\tilde{A}S+\tilde{B}\tilde{S}_{2}^{\mathrm{I}}+\tilde{v}_{\mathrm{th}}) \end{bmatrix} \geq0, \\ &\quad\qquad\qquad\qquad\qquad\qquad\qquad\qquad \bigl(S,\tilde {S}_{2}^{\mathrm{I}}\bigr)\in\overline{ \mathbb{R}}_{+}^{n_{\mathrm{E}}+q}\times\overline {\mathbb{R}}_{+}^{n_{\mathrm{I}}-q}, \end{aligned} $$

or, equivalently,

40 $$\begin{aligned} &2S^{\mathrm{T}}P\tilde{f}\bigl(\tilde{A}S+\tilde{B}\tilde{S}_{2}^{\mathrm{I}}+ \tilde {v}_{\mathrm{th}}\bigr) \leq S^{\mathrm{T}}QS+\tilde{f}^{\mathrm{T}} \bigl(\tilde{A}S+\tilde {B}\tilde{S}_{2}^{\mathrm{I}}+ \tilde{v}_{\mathrm{th}}\bigr)R\tilde{f}\bigl(\tilde{A}S+\tilde {B} \tilde{S}_{2}^{\mathrm{I}}+\tilde{v}_{\mathrm{th}}\bigr), \\ &\qquad\qquad\qquad\qquad\qquad\qquad\qquad\qquad\qquad \bigl(S,\tilde{S}_{2}^{\mathrm{I}} \bigr)\in\overline {\mathbb{R}}_{+}^{n_{\mathrm{E}}+q}\times\overline{ \mathbb{R}}_{+}^{n_{\mathrm{I}}-q}. \end{aligned}$$

Now, using (40) it follows from (38) that

41 $$\begin{aligned} &\dot{V}\bigl(S,\tilde{S}_{2}^{\mathrm{I}}\bigr) \leq S^{\mathrm{T}}(-PL-LP+Q)S+\tilde{f}^{\mathrm{T}}\bigl(\tilde{A}S+ \tilde{B}\tilde{S}_{2}^{\mathrm{I}}+\tilde{v}_{\mathrm{th}}\bigr)R \tilde {f}\bigl(\tilde{A}S+\tilde{B}\tilde{S}_{2}^{\mathrm{I}}+\tilde{v}_{\mathrm{th}}\bigr), \\ &\qquad\qquad\qquad\qquad\qquad\qquad\qquad\qquad\qquad \bigl(S,\tilde{S}_{2}^{\mathrm{I}} \bigr)\in\overline {\mathbb{R}}_{+}^{n_{\mathrm{E}}+q}\times\overline{ \mathbb{R}}_{+}^{n_{\mathrm{I}}-q}. \end{aligned}$$

Next, since $A^{\mathrm{IE}}\geq\geq0$, $S^{\mathrm{E}}\geq\geq0$, and $f_{i}(x)$, $x\in\mathbb{R}$, $i=q+1,\ldots,n_{\mathrm{I}}$, are nondecreasing functions, it follows that $\tilde{f}_{2}^{\mathrm{I}}(A_{2}^{\mathrm{IE}}S^{\mathrm{E}}+\tilde{v}^{\mathrm{I}}_{\mathrm{th2}})\geq\geq\tilde{f}_{2}^{\mathrm{I}}(\tilde {v}^{\mathrm{I}}_{\mathrm{th2}})$, $S^{\mathrm{E}}\in\overline{\mathbb{R}}_{+}^{n_{\mathrm{E}}}$. Since $f_{i}(x)\geq x$, $x\in\mathbb{R}$, $i=q+1,\ldots,n_{\mathrm{I}}$, it follows that $\tilde{f}_{2}^{\mathrm{I}}(\tilde{v}^{\mathrm{I}}_{\mathrm{th2}})\geq \geq\tilde{v}^{\mathrm{I}}_{\mathrm{th2}}$, and hence, it follows from (35) that

42 $$ \dot{\tilde{S}}_{2}^{\mathrm{I}}(t)\geq\geq-L_{2}^{\mathrm{I}} \tilde{S}_{2}^{\mathrm{I}}(t)+\tilde{v}^{\mathrm{I}}_{\mathrm{th2}}(t),\quad \tilde{S}_{2}^{\mathrm{I}}(0)=\tilde {S}_{20}^{\mathrm{I}},\quad t\geq0, $$

or, equivalently,

43 $$\begin{aligned} \dot{S}^{\mathrm{I}}_{i}(t)&\geq-\frac{1}{\tau^{\mathrm{I}}_{i}}S^{\mathrm{I}}_{i}(t)+v^{\mathrm{I}}_{\mathrm{th}i}(t) \\ &\geq-\frac{1}{\tau^{\mathrm{I}}_{i}}S^{\mathrm{I}}_{i}(t)+\eta _{i},\quad S^{\mathrm{I}}_{i}(0)=S^{\mathrm{I}}_{i0}, \quad t\geq0,\quad i=q+1,\ldots, n_{\mathrm{I}}. \end{aligned}$$

Now, consider the dynamical system

44 $$ \dot{y}_{i}(t)=-\frac{1}{\tau^{\mathrm{I}}_{i}} y_{i}(t)+ \eta_{i},\quad y(0)=S^{\mathrm{I}}_{i0},\quad t\geq0,\quad i=q+1, \ldots,n_{\mathrm{I}}, $$

and note that

45 $$ y_{i}(t)=\bigl(S^{\mathrm{I}}_{i0}- \tau^{\mathrm{I}}_{i}\eta_{i}\bigr)e^{-\frac{1}{\tau^{\mathrm{I}}_{i}}t}+ \tau^{\mathrm{I}}_{i}\eta_{i},\quad i=q+1, \ldots,n_{\mathrm{I}}. $$

Using the comparison principle ([23], p. 126), it follows that $S^{\mathrm{I}}_{i}(t)\geq y_{i}(t)\geq\min\{S^{\mathrm{I}}_{i0},\tau ^{\mathrm{I}}_{i}\eta_{i}\}$, $t\geq0$, $i=q+1,\ldots,n_{\mathrm{I}}$, or, equivalently,

46 $$ \tilde{S}_{2}^{\mathrm{I}}(t)\geq\geq\bigl[\min\bigl\{ S^{\mathrm{I}}_{q+1}(0), \tau _{q+1}^{\mathrm{I}} {\eta_{q+1}}\bigr\} ,\ldots,\min\bigl\{ S^{\mathrm{I}}_{n_{\mathrm{I}}}(0), \tau _{n_{\mathrm{I}}}^{\mathrm{I}} {\eta_{n_{\mathrm{I}}}}\bigr\} \bigr]^{\mathrm{T}}, \quad t\geq0. $$

Since $\tilde{B}\leq\leq0$, it follows that $\tilde{B}\tilde{S}^{\mathrm{I}}_{2}(t) \leq\leq\tilde{B}w$, $t\geq0$, where $w=[\min\{S^{\mathrm{I}}_{q+1}(0), \tau_{q+1}^{\mathrm{I}}{\eta_{q+1}}\},\ldots, \min\{S^{\mathrm{I}}_{n_{\mathrm{I}}}(0), \tau_{n_{\mathrm{I}}}^{\mathrm{I}}{\eta_{n_{\mathrm{I}}}}\} ]^{\mathrm{T}}$, and hence, $\tilde{v}_{\mathrm{th}}(t)+\tilde{B}\tilde{S}^{\mathrm{I}}_{2}(t) \leq\leq\tilde{v}_{\mathrm{th}}(t)+ \tilde{B}w\leq\leq0$, $t\geq0$. Thus, $\tilde{f}(\tilde{A}S+\tilde{B}\tilde{S}_{2}^{\mathrm{I}}+\tilde{v}_{\mathrm{th}})\leq\leq\tilde{f}(\tilde{A}S)$, $(S,\tilde{S}_{2}^{\mathrm{I}})\in \overline{\mathbb{R}}_{+}^{n_{\mathrm{E}}+q}\times\overline{\mathbb {R}}_{+}^{n_{\mathrm{I}}-q}$, and since $R\in\mathbb{R}^{(n_{\mathrm{E}}+q) \times (n_{\mathrm{E}}+q)}$ is a diagonal positive-definite matrix, it follows that

$$\begin{aligned} &\tilde{f}^{\mathrm{T}}\bigl(\tilde{A}S+\tilde{B}\tilde{S}_{2}^{\mathrm{I}}+ \tilde {v}_{\mathrm{th}}\bigr)R\tilde{f}\bigl(\tilde{A}S+\tilde{B} \tilde{S}_{2}^{\mathrm{I}}+\tilde {v}_{\mathrm{th}}\bigr)\leq \tilde{f}^{\mathrm{T}}(\tilde{A}S)R\tilde{f}(\tilde{A}S), \\ &\qquad\qquad\qquad\qquad\qquad\qquad\qquad\qquad \bigl(S,\tilde{S}_{2}^{\mathrm{I}}\bigr)\in\overline{ \mathbb{R}}_{+}^{n_{\mathrm{E}}+q}\times\overline{\mathbb{R}}_{+}^{n_{\mathrm{I}}-q}. \end{aligned}$$

Next, it follows from (41) that

47 $$\begin{aligned} &\dot{V}\bigl(S,\tilde{S}_{2}^{\mathrm{I}}\bigr)\leq S^{\mathrm{T}}(-PL-LP+Q)S+\tilde{f}^{\mathrm{T}}(\tilde{A}S)R\tilde{f}( \tilde{A}S), \\ &\qquad\qquad\qquad\qquad\qquad\qquad\qquad\qquad\qquad \bigl(S,\tilde{S}_{2}^{\mathrm{I}}\bigr)\in \overline{ \mathbb{R}}_{+}^{n_{\mathrm{E}}+q}\times\overline{\mathbb {R}}_{+}^{n_{\mathrm{I}}-q}. \end{aligned}$$

Since $f_{i}^{2}(x)\leq x^{2}$, $x\in\mathbb{R}$, for $f_{i}(\cdot)$, $i=1,\ldots,n_{\mathrm{E}}+q$, given by (9), and $R\in\mathbb {R}^{(n_{\mathrm{E}}+q) \times(n_{\mathrm{E}}+q)}$ is a diagonal positive-definite matrix, it follows that

48 $$ f^{\mathrm{T}}(\tilde{A}S)Rf(\tilde{A}S)\leq S^{\mathrm{T}} \tilde{A}^{\mathrm{T}}R\tilde{A}S, \quad S\in\overline{\mathbb{R}}_{+}^{n_{\mathrm{E}}+q}. $$

Now, it follows from (37) and (47) that

49 $$ \dot{V}\bigl(S,\tilde{S}_{2}^{\mathrm{I}}\bigr) \leq-S^{\mathrm{T}}\varOmega S\leq-\lambda_{\min }(\varOmega)\Vert S \Vert _{2}^{2},\quad \bigl(S,\tilde{S}_{2}^{\mathrm{I}} \bigr)\in\overline{\mathbb {R}}_{+}^{n_{\mathrm{E}}+q}\times\overline{ \mathbb{R}}_{+}^{n_{\mathrm{I}}-q}. $$

Thus, it follows from Theorem 4.1 of [23] that if $\varOmega>0$, then (34) and (35) is globally exponentially stable with respect to *S* uniformly in $\tilde {S}_{20}^{\mathrm{I}}$, and hence, (34) and (35) is globally exponentially partially synchronized.

Alternatively, if $\varOmega\geq0$ and $\mathcal{N}(\varOmega)=\operatorname {span}(\mathbf{e}_{n_{\mathrm{E}}+q})$ holds, then $\dot{V}(S(t),\tilde {S}_{2}^{\mathrm{I}}(t))\leq0$, $t\geq0$, and hence, $V(S(t))\leq V(S_{0})$ for all $t\geq0$. Next, since *P* is positive definite and $\dot {V}(S(t),\tilde{S}_{2}^{\mathrm{I}}(t))$ is a nonincreasing function of time, it follows that $V(S(t))$ is bounded for all $t\geq0$, and hence, $S(t)$ is bounded for all $t\geq0$, which further implies that $S^{\mathrm{E}}(t)$ is bounded for all $t\geq0$. Thus, $\tilde{f}^{\mathrm{I}}_{2}(A_{2}^{\mathrm{IE}}S^{\mathrm{E}}(t)+\tilde{v}^{\mathrm{I}}_{\mathrm{th2}}(t))$ is bounded for all $t\geq0$, and hence, there exists $\tilde{f}^{\mathrm{I}}_{2\max}$ such that $\tilde{f}^{\mathrm{I}}_{2\max}\geq\geq \tilde {f}^{\mathrm{I}}_{2}(A_{2}^{\mathrm{IE}}S^{\mathrm{E}}(t)+\tilde{v}^{\mathrm{I}}_{\mathrm{th2}}(t))$, $t\geq0$. Now, it follows from (35) that

50 $$ \dot{\tilde{S}}_{2}^{\mathrm{I}}(t)\leq\leq-L_{2}^{\mathrm{I}} \tilde{S}_{2}^{\mathrm{I}}(t)+\tilde{f}^{\mathrm{I}}_{2\max},\quad \tilde{S}_{2}^{\mathrm{I}}(0)=\tilde {S}_{20}^{\mathrm{I}}, \quad t\geq0. $$

Next, consider the dynamical system

51 $$ \dot{x}(t)=-L_{2}^{\mathrm{I}}x(t)+\tilde{f}^{\mathrm{I}}_{2\max},\quad x(0)=\tilde {S}_{20}^{\mathrm{I}},\quad t\geq0, $$

and note that since $L_{2}^{\mathrm{I}}$ is a diagonal positive-definite matrix, it follows that $x(t)$ is bounded for all $t\geq0$. Using the vector comparison principle [23], it follows from (50) and (51) that $\tilde {S}_{2}^{\mathrm{I}}(t)\leq\leq x(t)$, $t\geq0$, and hence, $\tilde{S}_{2}^{\mathrm{I}}(t)$ is bounded for all $t\geq0$. Since $S(t)$ and $\tilde{S}_{2}^{\mathrm{I}}(t)$ are bounded for all $t\geq0$, it follows that $\ddot{V}(S(t), \tilde{S}_{2}^{\mathrm{I}}(t))$ is bounded for all $t\geq0$. Thus, $\dot {V}(S(t),\tilde{S}_{2}^{\mathrm{I}}(t))$ is uniformly continuous in *t*. Finally, it follows from Barbalat’s lemma ([23], p. 221) that $\dot{V}(S(t),\tilde{S}_{2}^{\mathrm{I}}(t))\to0$ as $t\to\infty$, which, since $\mathcal{N}(\varOmega)=\operatorname {span}(\mathbf{e}_{n_{\mathrm{E}}+q})$, implies that (34) and (35) is globally asymptotically partially synchronized. □

There is a body of experimental evidence that the mechanism of action of some anesthetic drugs is the prolongation of the inhibitory postsynaptic potential time constant [1, 2]. While Theorem 2 provides sufficient conditions for partial synchronization, it is not readily apparent how these conditions may be met as the inhibitory postsynaptic potential time constant increases. Next, we address this question for the specific activation function [7]

52 $$ f_{i}(x)= \textstyle\begin{cases} 0, & x< 0,\\ x, & 0\leq x\leq f_{\max},\\ f_{\max}, & x>f_{\max}, \end{cases} $$

where $f_{\max}$ denotes the maximum firing rate.

### Theorem 3

*Consider the excitatory–inhibitory network given by* (30), (31), *and* (32) *with the vector activation functions defined by* (27) *and* (28), *and*$f_{i}(\cdot)$*given by* (52). *If*$\tilde{v}_{\mathrm{th2}}^{\mathrm{I}}(t)\gg 0$, $t\geq0$, *and the inhibitory time constants are such that*$\tilde {S}_{20}^{\mathrm{I}}-(L_{2}^{\mathrm{I}})^{-1}\min\{f_{\max}\mathbf{e}, \eta\}\ll0$*and*

53 $$ A^{\mathrm{EE}}\max\bigl\{ S_{0}^{\mathrm{E}}, f_{\max}\bigl(L^{\mathrm{E}}\bigr)^{-1}\mathbf{e}\bigr\} +A_{2}^{\mathrm{EI}}\bigl[\bigl(L_{2}^{\mathrm{I}} \bigr)^{-1}\min\{f_{\max}\mathbf{e}, \eta\} -\varepsilon \mathbf{e}\bigr]+\beta\leq\leq0, $$

*where*$\eta\triangleq[\eta_{q+1},\ldots,\eta_{n_{\mathrm{I}}}]$, $\eta _{i}=\min_{t\geq0}v_{\mathrm{th}i}^{\mathrm{I}}(t)$, $i=q+1,\ldots,n_{\mathrm{I}}$, $\max\{x,y\}\triangleq[\max\{x_{1}, y_{1}\},\ldots, \max\{x_{n},y_{n}\} ]^{\mathrm{T}}$, $x,y\in\mathbb{R}^{n}$, $\min\{x,y\}\triangleq[\min\{x_{1}, y_{1}\},\ldots, \min\{x_{n},y_{n}\}]^{\mathrm{T}}$, $x,y\in\mathbb{R}^{n}$, $\beta \triangleq[\beta_{1},\ldots,\beta_{n_{\mathrm{E}}}]^{\mathrm{T}}$, $\beta _{i}\triangleq\max_{t\geq0}v_{\mathrm{th}i}^{\mathrm{E}}(t)$, $i=1,\ldots, n_{\mathrm{E}}$, *and*$\varepsilon>0$*is a small positive constant*, *then*$S^{\mathrm{E}}(t)\to0$*as*$t\to\infty$, *and hence*, (30), (31), *and* (32) *is globally asymptotically partially synchronized*.

### Proof

First, note that since $f_{i}(x)\leq f_{\max}$, $x\in\mathbb{R}$, $i=1,\ldots,n_{\mathrm{E}}$, it follows from (30) that

54 $$\begin{aligned} \dot{S}_{i}^{\mathrm{E}}(t)\leq-\frac{1}{\tau_{i}^{\mathrm{E}}}S_{i}^{\mathrm{E}}(t)+f_{\max },\quad S_{i}^{\mathrm{E}}(0)=S_{i0}^{\mathrm{E}},\quad t\geq0,\quad i=1,\ldots ,n_{\mathrm{E}}, \end{aligned}$$

and hence,

55 $$\begin{aligned} S_{i}^{\mathrm{E}}(t)\leq\bigl(S_{i0}^{\mathrm{E}}- \tau_{i}^{\mathrm{E}}f_{\max}\bigr)e^{-\frac {t}{\tau_{i}^{\mathrm{E}}}}+ \tau_{i}^{\mathrm{E}}f_{\max},\quad t\geq0, \quad i=1, \ldots,n_{\mathrm{E}}. \end{aligned}$$

Thus, $S_{i}^{\mathrm{E}}(t)\leq\max\{S_{i0}^{\mathrm{E}}, \tau_{i}^{\mathrm{E}}f_{\max}\} $, $t\geq0$, $i=1,\ldots,n_{\mathrm{E}}$, or, equivalently,

56 $$ S^{\mathrm{E}}(t)\leq\leq\max\bigl\{ S_{0}^{\mathrm{E}}, f_{\max}\bigl(L^{\mathrm{E}}\bigr)^{-1}\mathbf{e}\bigr\} ,\quad t\geq0. $$

Next, since $A_{2}^{\mathrm{IE}}\geq\geq0$, $S^{\mathrm{E}}(t)\ge\geq0$, $t\geq 0$, and $f_{i}(x)$, $x\in\mathbb{R}$, $i=q+1,\ldots,n_{\mathrm{I}}$, are nondecreasing functions, it follows that $\tilde{f}_{2}^{\mathrm{I}}(A_{2}^{\mathrm{IE}}S^{\mathrm{E}}+\tilde{v}^{\mathrm{I}}_{\mathrm{th2}})\geq\geq\tilde{f}_{2}^{\mathrm{I}}(\tilde{v}^{\mathrm{I}}_{\mathrm{th2}})$, $S^{\mathrm{E}}\in\overline{\mathbb {R}}_{+}^{n_{\mathrm{E}}}$. Since $f_{i}(x)\leq f_{\max}$, $x\in\mathbb{R}$, $i=q+1,\ldots,n_{\mathrm{I}}$, and $\tilde{v}^{\mathrm{I}}_{\mathrm{th2}}(t)\gg 0$, $t\geq0$, it follows that $\tilde{f}_{2}^{\mathrm{I}}(\tilde{v}^{\mathrm{I}}_{\mathrm{th2}}(t))=\min\{f_{\max}\mathbf{e}, \tilde{v}^{\mathrm{I}}_{\mathrm{th2}}(t)\}\geq \geq\min\{f_{\max}\mathbf{e}, \eta\}$, $t\geq0$. Now, it follows from (32) that

57 $$\begin{aligned} \dot{\tilde{S}}_{2}^{\mathrm{I}}(t)\geq\geq-L_{2}^{\mathrm{I}} \tilde{S}_{2}^{\mathrm{I}}(t)+\min\{f_{\max}\mathbf{e}, \eta\},\quad \tilde{S}_{2}^{\mathrm{I}}(0)=\tilde{S}_{20}^{\mathrm{I}},\quad t\geq0, \end{aligned}$$

and hence,

58 $$\begin{aligned} \tilde{S}_{2}^{\mathrm{I}}(t)\geq\geq{}&\bigl(\tilde{S}_{20}^{\mathrm{I}}- \bigl(L_{2}^{\mathrm{I}}\bigr)^{-1}\min\{f_{\max} \mathbf{e}, \eta\}\bigr)e^{-L_{2}^{\mathrm{I}}t} \\ &{}+\bigl(L_{2}^{\mathrm{I}} \bigr)^{-1}\min\{f_{\max}\mathbf{e}, \eta\},\quad t\geq0. \end{aligned}$$

Thus, it follows from (58) that $\tilde{S}_{2}^{\mathrm{I}}(t)\geq \geq(L_{2}^{\mathrm{I}})^{-1}\min\{f_{\max}\mathbf{e}, \eta\}$ as $t\to\infty $, and since $\tilde{S}_{20}^{\mathrm{I}}-(L_{2}^{\mathrm{I}})^{-1}\min\{f_{\max }\mathbf{e}, \eta\}\ll0$, there exists $t_{1}>0$ such that

59 $$ \tilde{S}_{2}^{\mathrm{I}}(t)\geq\geq\bigl(L_{2}^{\mathrm{I}} \bigr)^{-1}\min\{f_{\max}\mathbf {e}, \eta\}-\varepsilon \mathbf{e},\quad t\geq t_{1}. $$

Next, since $A_{1}^{\mathrm{EI}}\leq\leq0$ and $\tilde{S}_{1}^{\mathrm{I}}(t)\geq \geq0$, $t\geq0$, and $f_{i}(x)$, $x\in\mathbb{R}$, $i=1,\ldots,n_{\mathrm{E}}$, are nondecreasing functions, it follows from (30) that

60 $$\begin{aligned} &\dot{S}^{\mathrm{E}}(t)\leq\leq-L^{\mathrm{E}}S^{\mathrm{E}}(t)+f^{\mathrm{E}} \bigl(A^{\mathrm{EE}}S^{\mathrm{E}}(t)+A_{2}^{\mathrm{EI}} \tilde{S}_{2}^{\mathrm{I}}(t)+\tilde{v}_{\mathrm{th}}^{\mathrm{E}}(t) \bigr), \\ &\qquad\qquad\qquad\qquad\qquad\qquad\qquad\qquad\qquad S^{\mathrm{E}}(0)=S^{\mathrm{E}}_{0},\quad t\geq0. \end{aligned}$$

Now, since $A^{\mathrm{EE}}\geq\geq0$ and $A_{2}^{\mathrm{EI}}\leq\leq0$, it follows from (56) and (59) that

61 $$\begin{aligned} A^{\mathrm{EE}}S^{\mathrm{E}}(t)+A_{2}^{\mathrm{EI}} \tilde{S}_{2}^{\mathrm{I}}(t)+\tilde {v}_{\mathrm{th}}^{\mathrm{E}}(t) \leq\leq{}& A^{\mathrm{EE}}\max\bigl\{ S_{0}^{\mathrm{E}}, f_{\max }\bigl(L^{\mathrm{E}}\bigr)^{-1}\mathbf{e}\bigr\} \\ &{}+A_{2}^{\mathrm{EI}}\bigl[\bigl(L_{2}^{\mathrm{I}} \bigr)^{-1}\min\{f_{\max}\mathbf{e}, \eta\} -\varepsilon \mathbf{e}\bigr]+\beta \\ \leq\leq{}&0,\quad t\geq t_{1}. \end{aligned}$$

Finally, it follows from (60) and (61) that

62 $$\begin{aligned} \dot{S}^{\mathrm{E}}(t)\leq\leq-L^{\mathrm{E}}S^{\mathrm{E}}(t),\quad S^{\mathrm{E}}(0)=S^{\mathrm{E}}_{0},\quad t\geq t_{1}, \end{aligned}$$

which implies that $S^{\mathrm{E}}(t)\to0$ as $t\to\infty$, and hence, (30), (31), and (32) is globally asymptotically partially synchronized. □

Condition (53) is satisfied if $A^{\mathrm{EI}}_{2}$ does not contain any row of zeros and $L_{2}^{\mathrm{I}}$ is small enough or, equivalently, the time constants of the inhibitory neurons that themselves do not receive any inhibitory inputs are large enough. This condition captures an important aspect of the effect of the anesthetic cascade. In particular, as the anesthetic drug concentration increases, resulting in an increase in the inhibitory time constants, the excitatory synaptic drives that receive inhibitory inputs from inhibitory neurons, which themselves do not receive inhibitory inputs, converge to zero, reflecting a state of unconsciousness. Alternatively, if only a subset $\mathcal{E}_{1}$ of excitatory neurons receive inhibitory inputs from the inhibitory neurons in $\mathcal{I}_{1}$, then it can be shown that only the synaptic drives of the excitatory neurons in $\mathcal{E}_{1}$ will converge to zero as the time constants of the inhibitory neurons in $\mathcal{I}_{1}$ increase.

## Generalized Neural Population Models, Nonmonotonic Postsynaptic Potentials, and Drug Biphasic Response

The administration of increasing anesthetic doses can lead to a paradoxical state of excitement in the patient prior to decreases in the level of consciousness. This paradoxical boost in brain activity prior to hypnotic induction is known as drug biphasic response [21]. There is also a second biphasic surge in the EEG power as the patient emerges from unconsciousness. The model proposed in Sect. 2 does not capture such phenomena. Models that predict the aforementioned characteristics are of great clinical importance in providing the phenomenological trends of the anesthetic cascade. In this section, we incorporate a more complex model for the postsynaptic potential to account for the delay in peak amplitude of the postsynaptic potential that occurs after a neuron discharges ([11], Fig. 8.5).

To capture an initial overshoot in the synaptic drive dynamics in an attempt to see biphasic responses in our neural field model, we assume the functional form of the postsynaptic potential is given by

63 $$ \alpha_{i}^{(\mathrm{E},\mathrm{I})}(t)=B_{i}^{(\mathrm{E},\mathrm{I})} te^{-\frac{t}{\lambda_{i}^{(\mathrm{E},\mathrm{I})}}}. $$

In this case, it follows from (4) and (63) that

64 $$\begin{aligned} \frac{\mathrm{d}S_{i}^{(\mathrm{E},\mathrm{I})}(t)}{\mathrm{d}t}={}&\int_{-\infty}^{t} B_{i}^{(\mathrm{E},\mathrm{I})}e^{-\frac{t-\tau}{\lambda_{i}^{(\mathrm{E},\mathrm{I})}}}f_{i} \bigl(v_{i}^{(\mathrm{E},\mathrm{I})}(\tau )\bigr)\,\mathrm{d}\tau \\ &{}-\int _{-\infty}^{t} \frac{t-\tau}{\lambda_{i}^{(\mathrm{E},\mathrm{I})}}B_{i}^{(\mathrm{E},\mathrm{I})}e^{-\frac{t-\tau}{\lambda_{i}^{(\mathrm{E},\mathrm{I})}}} f_{i}\bigl(v_{i}^{(\mathrm{E},\mathrm{I})}(\tau)\bigr) \,\mathrm{d}\tau \\ ={}&\int_{-\infty}^{t} B_{i}^{(\mathrm{E},\mathrm{I})}e^{-\frac{t-\tau}{\lambda_{i}^{(\mathrm{E},\mathrm{I})}}}f_{i} \bigl(v_{i}^{(\mathrm{E},\mathrm{I})}(\tau)\bigr)\,\mathrm{d}\tau- \frac{S_{i}^{(\mathrm{E},\mathrm{I})}(t)}{\lambda_{i}^{(\mathrm{E},\mathrm{I})}}. \end{aligned}$$

Now, defining

65 $$\begin{aligned} \overline{S_{i}}^{(\mathrm{E},\mathrm{I})}(t)&\triangleq \int_{-\infty}^{t} B_{i}^{(\mathrm{E},\mathrm{I})}e^{-\frac{t-\tau}{\lambda_{i}^{(\mathrm{E},\mathrm{I})}}}f_{i} \bigl(v_{i}^{(\mathrm{E},\mathrm{I})}(\tau )\bigr)\,\mathrm{d}\tau, \end{aligned}$$

it follows that

66 $$\begin{aligned} \frac{\mathrm{d}\overline{S_{i}}^{(\mathrm{E},\mathrm{I})}(t)}{\mathrm{d}t}&=-\frac {1}{\lambda_{i}^{(\mathrm{E},\mathrm{I})}}\overline{S_{i}}^{(\mathrm{E},\mathrm{I})}(t)+B_{i}^{(\mathrm{E},\mathrm{I})}f_{i} \bigl(v_{i}^{(\mathrm{E},\mathrm{I})}(t)\bigr). \end{aligned}$$

Next, it follows from (64) and (65) that

67 $$\begin{aligned} \frac{\mathrm{d}S_{i}^{(\mathrm{E},\mathrm{I})}(t)}{\mathrm{d}t}&=\overline{S_{i}}^{(\mathrm{E},\mathrm{I})}(t)- \frac{S_{i}^{(\mathrm{E},\mathrm{I})}(t)}{\lambda_{i}^{(\mathrm{E},\mathrm{I})}}. \end{aligned}$$

Differentiating (67) with respect to time, we obtain

68 $$\begin{aligned} \frac{\mathrm{d}^{2}S_{i}^{(\mathrm{E},\mathrm{I})}(t)}{\mathrm{d}t^{2}}={}&\frac{\mathrm{d}\overline {S_{i}}^{(\mathrm{E},\mathrm{I})}(t)}{\mathrm{d}t}-\frac{1}{\lambda_{i}^{(\mathrm{E},\mathrm{I})}}\frac {\mathrm{d}S_{i}^{(\mathrm{E},\mathrm{I})}(t)}{\mathrm{d}t} \\ ={}&{-}\frac{1}{\lambda_{i}^{(\mathrm{E},\mathrm{I})}}\overline{S_{i}}^{(\mathrm{E},\mathrm{I})}(t)+B_{i}^{(\mathrm{E},\mathrm{I})}f_{i} \bigl(v_{i}^{(\mathrm{E},\mathrm{I})}(t)\bigr)-\frac{1}{\lambda_{i}^{(\mathrm{E},\mathrm{I})}} \frac{\mathrm{d}S_{i}^{(\mathrm{E},\mathrm{I})}(t)}{\mathrm{d}t} \\ ={}&{-}\frac{1}{\lambda_{i}^{(\mathrm{E},\mathrm{I})}} \biggl(\frac{\mathrm{d}S_{i}^{(\mathrm{E},\mathrm{I})}(t)}{\mathrm{d}t}+\frac{S_{i}^{(\mathrm{E},\mathrm{I})}(t)}{\lambda_{i}^{(\mathrm{E},\mathrm{I})}} \biggr)+B_{i}^{(\mathrm{E},\mathrm{I})}f_{i} \bigl(v_{i}^{(\mathrm{E},\mathrm{I})}(t)\bigr) \\ &{}-\frac{1}{\lambda_{i}^{(\mathrm{E},\mathrm{I})}}\frac{\mathrm{d}S_{i}^{(\mathrm{E},\mathrm{I})}(t)}{\mathrm{d}t} \\ ={}&{-}\frac{2}{\lambda_{i}^{(\mathrm{E},\mathrm{I})}}\frac{\mathrm{d}S_{i}^{(\mathrm{E},\mathrm{I})}(t)}{\mathrm{d}t}- \biggl(\frac{1}{\lambda_{i}^{(\mathrm{E},\mathrm{I})}} \biggr)^{2}S_{i}^{(\mathrm{E},\mathrm{I})}(t)+B_{i}^{(\mathrm{E},\mathrm{I})}f_{i} \bigl(v_{i}^{(\mathrm{E},\mathrm{I})}(t)\bigr). \end{aligned}$$

Now, using the expressions for the excitatory and inhibitory voltage given by (2) and (3), respectively, it follows that

69 $$\begin{aligned} \frac{\mathrm{d}^{2}S_{i}^{\mathrm{E}}(t)}{\mathrm{d}t^{2}}={}&{-}\frac{2}{\lambda_{i}^{\mathrm{E}}}\frac{\mathrm{d}S_{i}^{\mathrm{E}}(t)}{\mathrm{d}t}- \biggl(\frac{1}{\lambda_{i}^{\mathrm{E}}} \biggr)^{2}S_{i}^{\mathrm{E}}(t)+B_{i}^{\mathrm{E}}f_{i} \Biggl(\sum_{j=1,j\neq i}^{n_{\mathrm{E}}}A_{ij}^{\mathrm{EE}}S_{j}^{\mathrm{E}}(t) \\ &{}+\sum_{j'=1}^{n_{\mathrm{I}}}A_{ij'}^{\mathrm{EI}}S_{j'}^{\mathrm{I}}(t)+v_{\mathrm{th}i}^{\mathrm{E}}(t) \Biggr),\quad i=1,\ldots,n_{\mathrm{E}}, \end{aligned}$$

70 $$\begin{aligned} \frac{\mathrm{d}^{2}S_{i}^{\mathrm{I}}(t)}{\mathrm{d}t^{2}}={}&{-}\frac{2}{\lambda_{i}^{\mathrm{I}}}\frac{\mathrm{d}S_{i}^{\mathrm{I}}(t)}{\mathrm{d}t}- \biggl(\frac{1}{\lambda_{i}^{\mathrm{I}}} \biggr)^{2}S_{i}^{\mathrm{I}}(t)+B_{i}^{\mathrm{I}}f_{i} \Biggl(\sum_{j=1}^{n_{\mathrm{E}}}A_{ij}^{\mathrm{IE}}S_{j}^{\mathrm{E}}(t) \\ &{}+\sum_{j'=1,j'\neq i}^{n_{\mathrm{I}}}A_{ij'}^{\mathrm{II}}S_{j'}^{\mathrm{I}}(t)+v_{\mathrm{th}i}^{\mathrm{I}}(t) \Biggr),\quad i=1,\ldots ,n_{\mathrm{I}}. \end{aligned}$$

The above analysis reveals that an alternative form to (8) for capturing the neuroelectric behavior of biological excitatory and inhibitory neuronal networks can be given by the second-order differential equations

71 $$\begin{aligned} &\frac{\mathrm{d^{2}}S_{i}(t)}{\mathrm{d}t^{2}}=-\frac{2}{\tau_{i}}\frac{\mathrm{d}S_{i}(t)}{\mathrm{d}t}- \frac{1}{\tau_{i}^{2}}S_{i}(t)+B_{i}f_{i} \Biggl( \sum_{j=1}^{n}A_{ij}S_{j}(t)+v_{\mathrm{th}i}(t) \Biggr), \\ &\qquad\qquad\qquad\qquad S_{i}(0)=S_{i0},\quad\dot{S}_{i}(0)= \dot{S}_{i0},\quad t\geq 0,\quad i=1,\ldots,n. \end{aligned}$$

To address the problem of partial synchronization for the model given by (69) and (70), the vector–matrix form of (69) and (70) can be written as

72 $$\begin{aligned} \ddot{S}^{\mathrm{E}}(t)={}&{-}2L^{\mathrm{E}} \dot{S}^{\mathrm{E}}(t)-\bigl(L^{\mathrm{E}}\bigr)^{2} S^{\mathrm{E}}(t)+f^{\mathrm{E}}\bigl(A^{\mathrm{EE}}S^{\mathrm{E}}(t)+A^{\mathrm{EI}}S^{\mathrm{I}}(t)+ \tilde{v}_{\mathrm{th}}^{\mathrm{E}}(t)\bigr), \\ &{}\qquad\qquad\qquad\qquad\qquad S^{\mathrm{E}}(0)=S^{\mathrm{E}}_{0},\quad \dot{S}^{\mathrm{E}}(0)=\dot{S}^{\mathrm{E}}_{0},\quad t\geq0, \end{aligned}$$

73 $$\begin{aligned} \ddot{S}^{\mathrm{I}}(t)={}&{-}2L^{\mathrm{I}} \dot{S}^{\mathrm{I}}(t)-\bigl(L^{\mathrm{I}}\bigr)^{2} S^{\mathrm{I}}(t)+f^{\mathrm{I}}\bigl(A^{\mathrm{IE}}S^{\mathrm{E}}(t)+A^{\mathrm{II}}S^{\mathrm{I}}(t)+ \tilde{v}_{\mathrm{th}}^{\mathrm{I}}(t)\bigr), \\ &{}\quad\qquad\qquad\qquad\qquad\qquad\qquad S^{\mathrm{I}}(0)=S^{\mathrm{I}}_{0},\quad \dot{S}^{\mathrm{I}}(0)=\dot{S}^{\mathrm{I}}_{0}, \end{aligned}$$

where all vector–matrix notation are defined as in Sect. 4. Once again, we partition the state vector $S^{\mathrm{I}}(t)$, $t\geq0$, as in Sect. 4 so that the neuronal connectivity matrix is partitioned as in (29), and hence, (72) and (73) can be written as

74 $$\begin{aligned} \ddot{S}^{\mathrm{E}}(t)={}&{-}2L^{\mathrm{E}} \dot{S}^{\mathrm{E}}(t)-\bigl(L^{\mathrm{E}}\bigr)^{2} S^{\mathrm{E}}(t)+f^{\mathrm{E}}\bigl(A^{\mathrm{EE}}S^{\mathrm{E}}(t)+A^{\mathrm{EI}}_{1} \tilde {S}_{1}^{\mathrm{I}}(t)+A^{\mathrm{EI}}_{2} \tilde{S}_{2}^{\mathrm{I}}(t) \\ &{}+\tilde{v}_{\mathrm{th}}^{\mathrm{E}}(t)\bigr),\quad S^{\mathrm{E}}(0)=S^{\mathrm{E}}_{0},\quad \dot{S}^{\mathrm{E}}(0)=\dot{S}^{\mathrm{E}}_{0},\quad t\geq0, \end{aligned}$$

75 $$\begin{aligned} \ddot{\tilde{S}}_{1}^{\mathrm{I}}(t)={}&{-}2L_{1}^{\mathrm{I}} \dot{\tilde{S}}_{1}^{\mathrm{I}}(t)-\bigl(L_{1}^{\mathrm{I}} \bigr)^{2} \tilde{S}_{1}^{\mathrm{I}}(t)+ \tilde{f}^{\mathrm{I}}_{1}\bigl(A_{1}^{\mathrm{IE}}S^{\mathrm{E}}(t)+A_{1}^{\mathrm{II}} \tilde{S}_{1}^{\mathrm{I}}(t)+A_{2}^{\mathrm{II}} \tilde{S}_{2}^{\mathrm{I}}(t) \\ &{}+\tilde{v}^{\mathrm{I}}_{\mathrm{th1}}(t)\bigr),\quad \tilde{S}_{1}^{\mathrm{I}}(0)= \tilde{S}^{\mathrm{I}}_{10},\quad\dot{\tilde{S}}_{1}^{\mathrm{I}}(0)= \dot {\tilde{S}}^{\mathrm{I}}_{10}, \end{aligned}$$

76 $$\begin{aligned} \ddot{\tilde{S}}_{2}^{\mathrm{I}}(t)={}&{-}2L_{2}^{\mathrm{I}} \dot{\tilde{S}}_{2}^{\mathrm{I}}(t)-\bigl(L_{2}^{\mathrm{I}} \bigr)^{2} \tilde{S}_{2}^{\mathrm{I}}(t)+ \tilde{f}^{\mathrm{I}}_{2}\bigl(A_{2}^{\mathrm{IE}}S^{\mathrm{E}}(t)+ \tilde{v}^{\mathrm{I}}_{\mathrm{th2}}(t)\bigr), \\ &{} \quad\qquad\qquad\qquad\qquad\qquad\qquad\tilde{S}_{2}^{\mathrm{I}}(0)= \tilde{S}_{20}^{\mathrm{I}},\quad \dot{\tilde{S}}_{2}^{\mathrm{I}}(0)=\dot{\tilde{S}}_{20}^{\mathrm{I}}, \end{aligned}$$

or, equivalently,

77 $$\begin{aligned} \ddot{S}(t)={}&{-}2L\dot{S}(t)-L^{2} S(t)+\tilde{f}\bigl( \tilde{A}S(t)+\tilde {B}\tilde{S}_{2}^{\mathrm{I}}(t)+ \tilde{v}_{\mathrm{th}}(t)\bigr), \\ &{}\quad \qquad\qquad\qquad\qquad\qquad S(0)=S_{0},\quad \dot {S}(0)=\dot{S}_{0},\quad t\geq0, \end{aligned}$$

78 $$\begin{aligned} \ddot{\tilde{S}}_{2}^{\mathrm{I}}(t)={}&{-}2L_{2}^{\mathrm{I}} \dot{\tilde{S}}_{2}^{\mathrm{I}}(t)-\bigl(L_{2}^{\mathrm{I}} \bigr)^{2} \tilde{S}_{2}^{\mathrm{I}}(t)+ \tilde{f}^{\mathrm{I}}_{2}\bigl(A_{2}^{\mathrm{IE}}S^{\mathrm{E}}(t)+ \tilde{v}^{\mathrm{I}}_{\mathrm{th2}}(t)\bigr), \\ &{} \quad\qquad\qquad\qquad\qquad\qquad\qquad\tilde{S}_{2}^{\mathrm{I}}(0)= \tilde{S}_{20}^{\mathrm{I}},\quad \dot{\tilde{S}}_{2}^{\mathrm{I}}(0)=\dot{\tilde{S}}_{20}^{\mathrm{I}}. \end{aligned}$$

Finally, defining $\hat{S}(t)\triangleq[S^{\mathrm{T}}(t)\ \dot{S}^{\mathrm{T}}(t)]^{\mathrm{T}}$, $\hat{S}^{\mathrm{I}}_{2}(t) \triangleq[(\tilde{S}^{\mathrm{I}}_{2}(t))^{\mathrm{T}}\ (\dot{\tilde{S}}^{\mathrm{I}}_{2}(t))^{\mathrm{T}}]^{\mathrm{T}}$, and $\hat{S}^{\mathrm{E}}(t)\triangleq[(\tilde{S}^{\mathrm{E}}(t))^{\mathrm{T}}\ (\dot{\tilde{S}}^{\mathrm{E}}(t))^{\mathrm{T}}]^{\mathrm{T}}$, (77) and (78) can be written as

79 $$\begin{aligned} \dot{\hat{S}}(t)&=-\varGamma\hat{S}(t)+\hat{f}\bigl(\hat{A} \hat{S}(t)+\hat {B}\hat{S}_{2}^{\mathrm{I}}(t)+ \hat{v}_{\mathrm{th}}(t)\bigr),\quad \hat{S}(0)=S_{0},\quad t\geq0, \end{aligned}$$

80 $$\begin{aligned} \dot{\hat{S}}_{2}^{\mathrm{I}}(t)&=- \varGamma^{\mathrm{I}}\hat{S}_{2}^{\mathrm{I}}(t)+\hat {f}^{\mathrm{I}}_{2}\bigl(\hat{A}_{2}^{\mathrm{IE}} \hat{S}^{\mathrm{E}}(t)+\hat{v}^{\mathrm{I}}_{\mathrm{th2}}(t)\bigr),\quad \hat{S}_{2}^{\mathrm{I}}(0)=\hat{S}_{20}^{\mathrm{I}}, \end{aligned}$$

where

$$\begin{aligned} \varGamma&\triangleq \begin{bmatrix} 0_{(n_{\mathrm{E}}+q)\times(n_{\mathrm{E}}+q)} &-I_{(n_{\mathrm{E}}+q)\times(n_{\mathrm{E}}+q)}\\ (L^{\mathrm{E}})^{2} & 2L^{\mathrm{E}} \end{bmatrix} , \qquad \hat{f}\triangleq \begin{bmatrix} 0_{(n_{\mathrm{E}}+q)}\\ \tilde{f} \end{bmatrix} , \\ \hat{A}&\triangleq \begin{bmatrix} 0_{(n_{\mathrm{E}}+q)\times(n_{\mathrm{E}}+q)} & 0_{(n_{\mathrm{E}}+q)\times(n_{\mathrm{E}}+q)}\\ \tilde{A}& 0_{(n_{\mathrm{E}}+q)\times(n_{\mathrm{E}}+q)} \end{bmatrix} , \qquad \hat{v}_{\mathrm{th}} \triangleq \begin{bmatrix} 0_{(n_{\mathrm{E}}+q)}\\ \tilde{v}_{\mathrm{th}} \end{bmatrix} , \\ \hat{B}&\triangleq \begin{bmatrix} 0_{(n_{\mathrm{E}}+q)\times(n_{\mathrm{I}}-q)} & 0_{(n_{\mathrm{E}}+q)\times(n_{\mathrm{I}}-q)} \\ \tilde{B}& 0_{(n_{\mathrm{E}}+q)\times(n_{\mathrm{I}}-q)} \end{bmatrix} , \qquad \hat{A}_{2}^{\mathrm{IE}} \triangleq \begin{bmatrix} 0_{(n_{\mathrm{I}}-q)\times n_{\mathrm{E}}} & 0_{(n_{\mathrm{I}}-q)\times n_{\mathrm{E}}}\\ A_{2}^{\mathrm{IE}}& 0_{(n_{\mathrm{I}}-q)\times n_{\mathrm{E}}} \end{bmatrix} , \\ \varGamma^{\mathrm{I}}&\triangleq \begin{bmatrix} 0_{(n_{\mathrm{I}}-q)\times(n_{\mathrm{I}}-q)} &-I_{(n_{\mathrm{I}}-q)\times(n_{\mathrm{I}}-q)}\\ (L_{2}^{\mathrm{I}})^{2} & 2L_{2}^{\mathrm{I}} \end{bmatrix} ,\qquad \hat{v}_{\mathrm{th2}}^{\mathrm{I}}\triangleq \begin{bmatrix} 0_{(n_{\mathrm{I}}-q)}\\ \tilde{v}_{\mathrm{th2}}^{\mathrm{I}} \end{bmatrix} ,\\ \hat{f}_{2}^{\mathrm{I}}&\triangleq \begin{bmatrix} 0_{(n_{\mathrm{I}}-q)}\\ \tilde{f}_{2}^{\mathrm{I}} \end{bmatrix} . \end{aligned}$$

The following proposition and definitions are needed for the main results of this section.

### Proposition 5

*Consider the excitatory–inhibitory network given by* (72) *and* (73) *with the vector activation functions defined by* (27) *and* (28), *and the synaptic drive defined by* (4) *with postsynaptic potential given by* (63). *Then*$S^{\mathrm{E}}(t)\geq\geq0$*and*$S^{\mathrm{I}}(t)\geq\geq0$*for all*$t\geq0$.

### Proof

Since $B_{i}^{\mathrm{E}}=B_{i}^{\mathrm{I}}=1$, it follows from (4) and (63) that

81 $$\begin{aligned} S_{i}^{\mathrm{E}}(0)=S_{i0}^{\mathrm{E}}&= \int _{-\infty}^{0}(-\tau) e^{\frac{\tau }{\lambda_{i}^{\mathrm{E}}}}f_{i} \bigl(v_{i}^{\mathrm{E}}(\tau)\bigr)\,\mathrm{d}\tau,\quad i=1,\ldots , n_{\mathrm{E}}. \end{aligned}$$

Now, letting $s\triangleq-\tau$, it follows from (81) that

82 $$\begin{aligned} S_{i0}^{\mathrm{E}}&= \int_{\infty}^{0}s e^{\frac{-s}{\lambda_{i}^{\mathrm{E}}}}f_{i}\bigl(v_{i}^{\mathrm{E}}(-s) \bigr) (-\mathrm{d}s) \\ &=\int_{0}^{\infty}s e^{\frac {-s}{\lambda_{i}^{\mathrm{E}}}}f_{i}\bigl(v_{i}^{\mathrm{E}}(-s) \bigr)\,\mathrm{d}s,\quad i=1,\ldots ,n_{\mathrm{E}}. \end{aligned}$$

Since $s e^{\frac{-s}{\lambda_{i}^{\mathrm{E}}}}f_{i}(v_{i}^{\mathrm{E}}(-s))\geq0$ for $s\in[0,\infty)$ and $i=1,\ldots, n_{\mathrm{E}}$, it follows from (82) that $S_{i0}^{\mathrm{E}}\geq0$ for $i=1,\ldots, n_{\mathrm{E}}$. Next, it follows from (64) that

83 $$\begin{aligned} \dot{S}_{i0}^{\mathrm{E}}+\frac{1}{\lambda_{i}^{\mathrm{E}}}S_{i0}^{\mathrm{E}}&= \int_{-\infty}^{0} e^{\frac{\tau}{\lambda_{i}^{\mathrm{E}}}}f_{i} \bigl(v_{i}^{\mathrm{E}}(\tau )\bigr)\,\mathrm{d}\tau\geq0,\quad i=1, \ldots, n_{\mathrm{E}}. \end{aligned}$$

Next, since $f_{i}(x)\geq0$, $x\in\mathbb{R}$, $i=1,\ldots,n_{\mathrm{E}}$, it follows from (72) that

84 $$\begin{aligned} &\ddot{S}^{\mathrm{E}}_{i}(t)+\frac{2}{\lambda^{\mathrm{E}}_{i}} \dot{S}_{i}^{\mathrm{E}}(t)+\frac{1}{(\lambda^{\mathrm{E}}_{i})^{2}}S^{\mathrm{E}}_{i}(t) \geq0, \\ &\quad\quad \quad \quad S_{i}^{\mathrm{E}}(0)=S_{i0}^{\mathrm{E}},\quad \dot{S}_{i}^{\mathrm{E}}(0)=\dot {S}_{i0}^{\mathrm{E}},\quad t\geq0,\quad i=1,\ldots,n_{\mathrm{E}}. \end{aligned}$$

Now, letting

85 $$\begin{aligned} u_{i}^{\mathrm{E}}(t)\triangleq\dot{S}_{i}^{\mathrm{E}}(t)+ \frac{1}{\lambda_{i}^{\mathrm{E}}}S_{i}^{\mathrm{E}}(t), \quad t\geq0,\quad i=1,\ldots, n_{\mathrm{E}}, \end{aligned}$$

it follows from (84) and (85) that

86 $$\begin{aligned} \dot{u}_{i}^{\mathrm{E}}(t)+\frac{1}{\lambda_{i}^{\mathrm{E}}}u_{i}^{\mathrm{E}}(t) \geq0, \quad u_{i}^{\mathrm{E}}(0)=\dot{S}_{i0}^{\mathrm{E}}+ \frac{1}{\lambda_{i}^{\mathrm{E}}}S_{i0}^{\mathrm{E}},\quad t\geq0,\quad i=1,\ldots, n_{\mathrm{E}}, \end{aligned}$$

and since $u_{i}^{\mathrm{E}}(0)=\dot{S}_{i0}^{\mathrm{E}}+\frac{1}{\lambda_{i}^{\mathrm{E}}}S_{i0}^{\mathrm{E}}\geq0$, $i=1,\ldots, n_{\mathrm{E}}$, it follows from (86) that $u_{i}^{\mathrm{E}}(t)\geq0$, $t\geq0$, $i=1,\ldots,n_{\mathrm{E}}$. Thus, it follows from (85) that

87 $$\begin{aligned} \dot{S}_{i}^{\mathrm{E}}(t)+\frac{1}{\lambda_{i}^{\mathrm{E}}}S_{i}^{\mathrm{E}}(t) \geq0,\quad S_{i}^{\mathrm{E}}(0)=S_{i0}^{\mathrm{E}},\quad t \geq0,\quad i=1,\ldots ,n_{\mathrm{E}}. \end{aligned}$$

Finally, since $S_{i0}^{\mathrm{E}}\geq0$, $i=1,\ldots,n_{\mathrm{E}}$, it follows from (87) that $S_{i}^{\mathrm{E}}(t)\geq0$, $t\geq0$, $i=1,\ldots,n_{\mathrm{E}}$. Similarly, it can be shown that $S_{i}^{\mathrm{I}}(t)\geq0$, $t\geq0$, $i=1,\ldots,n_{\mathrm{I}}$, and hence, $S^{\mathrm{E}}(t)\geq\geq0$ and $S^{\mathrm{I}}(t)\geq\geq0$ for all $t\geq0$. □

### Definition 4

The biological neural network given by (79) and (80) is said to be *globally asymptotically partially synchronized* if

$$\begin{aligned} \lim_{t\to\infty}\bigl\vert S_{i}(t)-S_{j}(t) \bigr\vert & =0, \\ \lim_{t\to\infty}\bigl\vert \dot{S}_{i}(t)- \dot{S}_{j}(t)\bigr\vert &= 0, \end{aligned}$$

for all $\hat{S}_{0}\in\overline{\mathbb{R}}^{2(n_{\mathrm{E}}+q)}_{+}$, $\hat {S}^{\mathrm{I}}_{20}\in\overline{\mathbb{R}}^{2(n_{\mathrm{I}}-q)}_{+}$, and $i=1,\ldots, n_{\mathrm{E}}+q$, $i\neq j$.

### Definition 5

The biological neural network given by (79) and (80) is said to be *globally exponentially partially synchronized* if there exist constants $p>0$ and $\rho>0$ such that

$$\begin{aligned} \bigl\vert S_{i}(t)-S_{j}(t)\bigr\vert & \leq\rho e^{-pt}\vert S_{i0}-S_{j0}\vert ,\quad t\geq0, \\ \bigl\vert \dot{S}_{i}(t)-\dot{S}_{j}(t)\bigr\vert &\leq\rho e^{-pt}\vert \dot{S}_{i0}-\dot {S}_{j0} \vert ,\quad t\geq0, \end{aligned}$$

for all $\hat{S}_{0}\in\overline{\mathbb{R}}^{2(n_{\mathrm{E}}+q)}_{+}$, $\hat {S}^{\mathrm{I}}_{20}\in\overline{\mathbb{R}}^{2(n_{\mathrm{I}}-q)}_{+}$, and $i=1,\ldots, n_{\mathrm{E}}+q$, $i\neq j$.

### Theorem 4

*Consider the excitatory–inhibitory network given by* (79) *and* (80) *with the vector activation functions defined by* (27) *and* (28). *If*$\tilde{v}_{\mathrm{th}}(t)\leq\leq-\tilde{B}\hat{w}$, $t\geq0$, *where*$\hat{w}\triangleq[\min\{S^{\mathrm{I}}_{q+1}(0), \tau _{q+1}^{\mathrm{I}}{\alpha_{q+1}}\},\ldots, \min\{S^{\mathrm{I}}_{n_{\mathrm{I}}}(0), \tau_{n_{\mathrm{I}}}^{\mathrm{I}}{\alpha_{n_{\mathrm{I}}}}\} ]^{\mathrm{T}}\in \mathbb{R}^{n_{\mathrm{I}}-q}$, $\alpha_{i} \triangleq\min\{\dot{S}^{\mathrm{I}}_{i0}+\frac{1}{\tau_{i}^{\mathrm{I}}} S_{i0}^{\mathrm{I}},\tau^{\mathrm{I}}_{i}\eta_{i}\} $, $i=q+1,\ldots,n_{\mathrm{I}}$, $\eta_{i}=\min_{t\geq0}v_{\mathrm{th}i}^{\mathrm{I}}(t)$, $i=q+1,\ldots,n_{\mathrm{I}}$, *and there exist positive*-*definite matricesP̂*, $\hat{Q}\in\mathbb{R}^{2(n_{\mathrm{E}}+q)\times 2(n_{\mathrm{E}}+q)}$*and a diagonal positive*-*definite matrix*$\hat{R}\in \mathbb{R}^{2(n_{\mathrm{E}}+q)\times2(n_{\mathrm{E}}+q)}$*such that*

88 $$ \begin{bmatrix} \hat{Q} & -\hat{P}\\ -\hat{P} & \hat{R} \end{bmatrix} \geq0, $$

*and*$\hat{\varOmega}>0$, *where*

89 $$ \hat{\varOmega}\triangleq\hat{P}\hat{L}+\hat{L}\hat{P}-\hat{Q}-\hat {A}^{\mathrm{T}}\hat{R}\hat{A}, $$

*then* (79) *and* (80) *is globally exponentially partially synchronized*. *Alternatively*, *if*$\hat{\varOmega}\geq0$*and*$\mathcal {N}(\hat{\varOmega})=\operatorname {span}([\mathbf{e}_{(n_{\mathrm{E}}+q)}^{\mathrm{T}}\ 0_{n_{\mathrm{E}}+q}^{\mathrm{T}}]^{\mathrm{T}}, [0_{n_{\mathrm{E}}+q}^{\mathrm{T}}\ \mathbf {e}_{(n_{\mathrm{E}}+q)}^{\mathrm{T}}]^{\mathrm{T}})$, *then* (79) *and* (80) *is globally asymptotically partially synchronized*.

### Proof

The proof follows as in the proof of Theorem 2 using the partial Lyapunov function candidate $\hat{V}:\overline{\mathbb {R}}_{+}^{2(n_{\mathrm{E}}+q)}\to\mathbb{R}$ given by $\hat{V}(\hat{S})=\hat {S}^{\mathrm{T}}\hat{P}\hat{S}$. Specifically, since $A^{\mathrm{IE}}\geq\geq0$, $S^{\mathrm{E}}\geq\geq0$, and $f_{i}(x)$, $x\in\mathbb{R}$, $i=q+1,\ldots ,n_{\mathrm{I}}$, are nondecreasing functions, it follows that $\tilde {f}_{2}^{\mathrm{I}}(A_{2}^{\mathrm{IE}}S^{\mathrm{E}}+\tilde{v}^{\mathrm{I}}_{\mathrm{th2}})\geq \geq\tilde{f}_{2}^{\mathrm{I}}(\tilde{v}^{\mathrm{I}}_{\mathrm{th2}})$, $S^{\mathrm{E}}\in \overline{\mathbb{R}}_{+}^{n_{\mathrm{E}}}$. Now, since $f_{i}(x)\geq x$, $x\in \mathbb{R}$, $i=q+1,\ldots,n_{\mathrm{I}}$, it follows that $\tilde {f}_{2}^{\mathrm{I}}(\tilde{v}^{\mathrm{I}}_{\mathrm{th2}})\geq\geq\tilde{v}^{\mathrm{I}}_{\mathrm{th2}}$, and hence, it follows from (78) that

90 $$\begin{aligned} &\ddot{\tilde{S}}_{2}^{\mathrm{I}}(t)\geq\geq-2L_{2}^{\mathrm{I}} \dot{\tilde {S}}_{2}^{\mathrm{I}}(t)-\bigl(L_{2}^{\mathrm{I}} \bigr)^{2} \tilde{S}_{2}^{\mathrm{I}}(t)+ \tilde{v}^{\mathrm{I}}_{\mathrm{th2}}(t), \\ &\quad \quad \quad \quad \quad \quad \quad \quad \quad \tilde{S}_{2}^{\mathrm{I}}(0)= \tilde{S}_{20}^{\mathrm{I}},\quad \dot{\tilde{S}}_{2}^{\mathrm{I}}(0)= \dot{\tilde{S}}_{20}^{\mathrm{I}},\quad t\geq0, \end{aligned}$$

or, equivalently,

91 $$\begin{aligned} \ddot{S}^{\mathrm{I}}_{i}(t)\geq{}&{-} \frac{2}{\tau^{\mathrm{I}}_{i}}\dot{S}_{i}^{\mathrm{I}}(t)-\frac{1}{(\tau_{i}^{\mathrm{I}})^{2}} S_{i}^{\mathrm{I}}(t)+v^{\mathrm{I}}_{\mathrm{th}i}(t) \\ \geq{}&{-}\frac{2}{\tau^{\mathrm{I}}_{i}}\dot{S}_{i}^{\mathrm{I}}(t)- \frac{1}{(\tau _{i}^{\mathrm{I}})^{2}} S_{i}^{\mathrm{I}}(t)+\eta_{i}, \\ &{}S_{i}^{\mathrm{I}}(0)=S_{i0}^{\mathrm{I}},\quad \dot{S}_{i}^{\mathrm{I}}(0)=\dot {S}_{i0}^{\mathrm{I}},\quad t\geq0,\quad i=q+1,\ldots,n_{\mathrm{I}}. \end{aligned}$$

Letting

92 $$ u_{i}^{\mathrm{I}}(t)\triangleq\dot{S}^{\mathrm{I}}_{i}(t)+ \frac{1}{\tau_{i}^{\mathrm{I}}} S_{i}^{\mathrm{I}}(t),\quad t\geq0, \quad i=1,\ldots, n_{\mathrm{I}}, $$

it follows from (91) that

93 $$ \dot{u}_{i}^{\mathrm{I}}(t)+\frac{1}{\tau_{i}^{\mathrm{I}}}u_{i}^{\mathrm{I}}(t) \geq\eta_{i},\quad u_{i}^{\mathrm{I}}(0)= \dot{S}^{\mathrm{I}}_{i0}(t)+\frac{1}{\tau_{i}^{\mathrm{I}}} S_{i0}^{\mathrm{I}},\quad t\geq0,\quad i=q+1,\ldots,n_{\mathrm{I}}, $$

and hence,

94 $$\begin{aligned} u_{i}^{\mathrm{I}}(t)&\geq\bigl(u_{i}^{\mathrm{I}}(0)- \tau^{\mathrm{I}}_{i}\eta_{i}\bigr)e^{-\frac {1}{\tau^{\mathrm{I}}_{i}}t}+ \tau^{\mathrm{I}}_{i}\eta_{i} \\ &\geq\min\biggl\{ \dot{S}^{\mathrm{I}}_{i0}+\frac{1}{\tau_{i}^{\mathrm{I}}} S_{i0}^{\mathrm{I}},\tau^{\mathrm{I}}_{i} \eta_{i}\biggr\} =\alpha_{i},\quad t\geq0,\quad i=q+1,\ldots ,n_{\mathrm{I}}. \end{aligned}$$

Next, it follows from (92) and (94) that

95 $$ \dot{S}^{\mathrm{I}}_{i}(t)+\frac{1}{\tau_{i}^{\mathrm{I}}} S_{i}^{\mathrm{I}}(t) \geq\alpha _{i},\quad S^{\mathrm{I}}_{i}(0)=S^{\mathrm{I}}_{i0},\quad t\geq0,\quad i=q+1,\ldots, n_{\mathrm{I}}, $$

and hence,

96 $$\begin{aligned} S^{\mathrm{I}}_{i}(t)&\geq\bigl(S^{\mathrm{I}}_{i0}- \tau^{\mathrm{I}}_{i}\alpha_{i}\bigr)e^{-\frac {1}{\tau^{\mathrm{I}}_{i}}t}+ \tau^{\mathrm{I}}_{i}\alpha_{i} \\ &\geq\min\bigl\{ S^{\mathrm{I}}_{i0},\tau^{\mathrm{I}}_{i} \alpha_{i}\bigr\} ,\quad t\geq0,\quad i=q+1,\ldots ,n_{\mathrm{I}}. \end{aligned}$$

Thus,

97 $$ \tilde{S}_{2}^{\mathrm{I}}(t)\geq\geq\bigl[\min\bigl\{ S^{\mathrm{I}}_{q+1}(0), \tau _{q+1}^{\mathrm{I}} {\alpha_{q+1}}\bigr\} ,\ldots, \min\bigl\{ S^{\mathrm{I}}_{n_{\mathrm{I}}}(0), \tau_{n_{\mathrm{I}}}^{\mathrm{I}} {\alpha_{n_{\mathrm{I}}}}\bigr\} \bigr]^{\mathrm{T}}, \quad t\geq0, $$

where $\alpha_{i}=\min\{\dot{S}^{\mathrm{I}}_{i0}(t)+\frac{1}{\tau_{i}^{\mathrm{I}}} S_{i0}^{\mathrm{I}},\tau^{\mathrm{I}}_{i}\eta_{i}\}$ and $\eta_{i}=\min_{t\geq 0}v_{\mathrm{th}i}^{\mathrm{I}}(t)$, $i=q+1,\ldots,n_{\mathrm{I}}$, $t\geq0$. Since $\tilde{B}\leq\leq0$, it follows that $\tilde{B}\tilde{S}^{\mathrm{I}}_{2}(t) \leq\leq\tilde{B}w$, $t\geq0$, where $w=[\min\{S^{\mathrm{I}}_{q+1}(0), \tau_{q+1}^{\mathrm{I}}{\alpha_{q+1}}\},\ldots, \min\{S^{\mathrm{I}}_{n_{\mathrm{I}}}(0), \tau_{n_{\mathrm{I}}}^{\mathrm{I}}{\alpha_{n_{\mathrm{I}}}}\} ]^{\mathrm{T}}$, and hence, $\tilde{v}_{\mathrm{th}}(t)+\tilde{B}\tilde{S}^{\mathrm{I}}_{2}(t)\leq\leq\tilde{v}_{\mathrm{th}}(t)+ \tilde{B}w\leq\leq0$, $t\geq0$. Thus, $\hat{B}\hat{S}_{2}^{\mathrm{I}}(t)+\hat{v}_{\mathrm{th}}(t)\leq\leq0$, and hence, $\hat{f}(\hat{A}\hat{S}(t)+\hat{B}\hat{S}_{2}^{\mathrm{I}}(t)+\hat {v}_{\mathrm{th}}(t))\leq\leq\hat{f}(\hat{A}\hat{S})$, $(\hat{S},\hat {S}_{2}^{\mathrm{I}})\in\overline{\mathbb{R}}_{+}^{2(n_{\mathrm{E}}+q)}\times\overline {\mathbb{R}}_{+}^{2(n_{\mathrm{I}}-q)}$. The remainder of the proof now follows as in the proof of Theorem 2. □

Finally, we analyze the partial synchronization of the excitatory–inhibitory network given by (74), (75), and (76) with the activation function given by (52) in the face of increasing inhibitory time constants.

### Theorem 5

*Consider the excitatory–inhibitory network given by* (74), (75), *and* (76) *with the vector activation functions defined by* (27) *and* (28), *and*$f_{i}(\cdot)$*given by* (52). *If*$\tilde{v}_{\mathrm{th2}}^{\mathrm{I}}(t)\gg 0$, $t\geq0$, *and the time constants of inhibitory neurons are such that*$\dot{\tilde{S}}_{20}^{\mathrm{I}}+L_{2}^{\mathrm{I}}\tilde{S}_{20}^{\mathrm{I}}-(L_{2}^{\mathrm{I}})^{-1}\min\{f_{\max}\mathbf{e}, \eta\}\ll0$, $\tilde {S}_{20}^{\mathrm{I}}-(L_{2}^{\mathrm{I}})^{-1}((L_{2}^{\mathrm{I}})^{-1}\min\{f_{\max }\mathbf{e}, \eta\}-\varepsilon\mathbf{e})\ll0$, *and*

98 $$\begin{aligned} &A^{\mathrm{EE}}\max\bigl\{ S^{\mathrm{E}}_{0}, \bigl(L^{\mathrm{E}}\bigr)^{-1} \max\bigl\{ \dot {S}_{0}^{\mathrm{E}}+L^{\mathrm{E}}S_{0}^{\mathrm{E}}, f_{\max}\bigl(L^{\mathrm{E}}\bigr)^{-1}\mathbf {e}\bigr\} \bigr\} \\ &\quad {}+A_{2}^{\mathrm{EI}}\bigl[\bigl(L_{2}^{\mathrm{I}} \bigr)^{-1}\bigl[\bigl(L_{2}^{\mathrm{I}} \bigr)^{-1}\min\{ f_{\max}\mathbf{e}, \eta\}-\varepsilon \mathbf{e}\bigr]-\varepsilon\mathbf {e}\bigr]+\beta\leq\leq0, \end{aligned}$$

*where*$\eta= [\eta_{q+1},\ldots,\eta_{n_{\mathrm{I}}}]$, $\eta_{i}=\min_{t\geq 0}v_{\mathrm{th}i}^{\mathrm{I}}(t)$, $i=q+1,\ldots,n_{\mathrm{I}}$, $\beta= [\beta _{1},\ldots,\beta_{n_{\mathrm{E}}}]^{\mathrm{T}}$, $\beta_{i}=\max_{t\geq0}v_{\mathrm{th}i}^{\mathrm{E}}(t)$, $i=1,\ldots, n_{\mathrm{E}}$, *and*$\varepsilon>0$*is a small positive constant*, *then*$S^{\mathrm{E}}(t)\to0$*as*$t\to\infty$, *and hence*, (74), (75), *and* (76) *are globally asymptotically partially synchronized*.

### Proof

First, note that since $f_{i}(x)\leq f_{\max}$, $x\in\mathbb{R}$, $i=1,\ldots,n_{\mathrm{E}}$, it follows from (74) that

99 $$\begin{aligned} &\ddot{S}^{\mathrm{E}}_{i}(t)+\frac{2}{\tau^{\mathrm{E}}_{i}} \dot{S}_{i}^{\mathrm{E}}(t)+\frac {1}{(\tau^{\mathrm{E}}_{i})^{2}}S^{\mathrm{E}}_{i}(t) \leq f_{\max}, \\ &\quad\quad \quad S_{i}^{\mathrm{E}}(0)=S_{i0}^{\mathrm{E}},\quad \dot{S}_{i}^{\mathrm{E}}(0)=\dot{S}_{i0}^{\mathrm{E}},\quad t\geq0,\quad i=1,\ldots,n_{\mathrm{E}}. \end{aligned}$$

Now, defining $u_{i}^{\mathrm{E}}(t)\triangleq\dot{S}^{\mathrm{E}}_{i}(t)+\frac {1}{\tau^{\mathrm{E}}_{i}}S_{i}^{\mathrm{E}}(t)$, $t\geq0$, $i=1,\ldots, n_{\mathrm{E}}$, it can be shown that

100 $$\begin{aligned} S_{i}^{\mathrm{E}}(t)&\leq\max \biggl\{ S^{\mathrm{E}}_{i0}, \tau^{\mathrm{E}}_{i} \max \biggl\{ \dot{S}_{i0}^{\mathrm{E}}+ \frac{1}{\tau_{i}^{\mathrm{E}}}S_{i0}^{\mathrm{E}}, \tau _{i}^{\mathrm{E}}f_{\max} \biggr\} \biggr\} ,\quad t\geq0, i=1,\ldots,n_{\mathrm{E}}, \end{aligned}$$

or, equivalently,

101 $$ S^{\mathrm{E}}(t)\leq\max\bigl\{ S^{\mathrm{E}}_{0}, \bigl(L^{\mathrm{E}}\bigr)^{-1} \max\bigl\{ \dot {S}_{0}^{\mathrm{E}}+L^{\mathrm{E}}S_{0}^{\mathrm{E}}, f_{\max}\bigl(L^{\mathrm{E}}\bigr)^{-1}\mathbf {e}\bigr\} \bigr\} ,\quad t\geq0. $$

Next, since $A_{2}^{\mathrm{IE}}\geq\geq0$, $S^{\mathrm{E}}(t)\ge\geq0$, $t\geq 0$, and $f_{i}(x)$, $x\in\mathbb{R}$, $i=q+1,\ldots,n_{\mathrm{I}}$, are nondecreasing functions, it follows that $\tilde{f}_{2}^{\mathrm{I}}(A_{2}^{\mathrm{IE}}S^{\mathrm{E}}+\tilde{v}^{\mathrm{I}}_{\mathrm{th2}})\geq\geq\tilde{f}_{2}^{\mathrm{I}}(\tilde{v}^{\mathrm{I}}_{\mathrm{th2}})$, $S^{\mathrm{E}}\in\overline{\mathbb {R}}_{+}^{n_{\mathrm{E}}}$. Since $f_{i}(x)\leq f_{\max}$, $x\in\mathbb{R}$, $i=q+1,\ldots,n_{\mathrm{I}}$, and $\tilde{v}^{\mathrm{I}}_{\mathrm{th2}}(t)\gg 0$, $t\geq0$, it follows that $\tilde{f}_{2}^{\mathrm{I}}(\tilde{v}^{\mathrm{I}}_{\mathrm{th2}}(t))=\min\{f_{\max}\mathbf{e}, \tilde{v}^{\mathrm{I}}_{\mathrm{th2}}(t)\}\geq \geq\min\{f_{\max}\mathbf{e}, \eta\}$, $t\geq0$. Now, it follows from (76) that

102 $$\begin{aligned} \ddot{\tilde{S}}_{2}^{\mathrm{I}}(t)\geq\geq{}&{-}2L_{2}^{\mathrm{I}} \dot{\tilde {S}}_{2}^{\mathrm{I}}(t)-\bigl(L_{2}^{\mathrm{I}} \bigr)^{2}\tilde{S}_{2}^{\mathrm{I}}(t) \\ &{}+\min \{f_{\max }\mathbf{e}, \eta\},\quad \tilde{S}_{2}^{\mathrm{I}}(0)= \tilde{S}_{20}^{\mathrm{I}},\quad \dot{\tilde {S}}_{2}^{\mathrm{I}}(0)=\dot{\tilde{S}}_{20}^{\mathrm{I}},\quad t\geq0. \end{aligned}$$

Next, defining

103 $$ u^{\mathrm{I}}(t)\triangleq\dot{\tilde{S}}_{2}^{\mathrm{I}}(t)+L_{2}^{\mathrm{I}} \tilde {S}_{2}^{\mathrm{I}}(t), \quad t\geq0, $$

it follows from (102) that

104 $$\begin{aligned} \dot{u}^{\mathrm{I}}(t)+L_{2}^{\mathrm{I}} u^{\mathrm{I}}(t) \geq\geq\min\{f_{\max }\mathbf{e}, \eta\},\qquad u^{\mathrm{I}}(0)=\dot{\tilde{S}}_{20}^{\mathrm{I}}+L_{2}^{\mathrm{I}} \tilde{S}_{20}^{\mathrm{I}},\quad t\geq0, \end{aligned}$$

and hence,

105 $$\begin{aligned} u^{\mathrm{I}}(t)\geq\geq{}&\bigl(\dot{\tilde{S}}_{20}^{\mathrm{I}}+L_{2}^{\mathrm{I}} \tilde {S}_{20}^{\mathrm{I}}-\bigl(L_{2}^{\mathrm{I}} \bigr)^{-1}\min\{f_{\max}\mathbf{e}, \eta\} \bigr)e^{-L_{2}^{\mathrm{I}}t} \\ &{}+\bigl(L_{2}^{\mathrm{I}}\bigr)^{-1}\min \{f_{\max}\mathbf{e}, \eta\},\quad t\geq0. \end{aligned}$$

Thus, it follows from (105) that $u^{\mathrm{I}}(t)\geq\geq (L_{2}^{\mathrm{I}})^{-1}\min\{f_{\max}\mathbf{e}, \eta\}$ as $t\to\infty$, and since $\dot{\tilde{S}}_{20}^{\mathrm{I}}+L_{2}^{\mathrm{I}}\tilde{S}_{20}^{\mathrm{I}}-(L_{2}^{\mathrm{I}})^{-1}\min\{f_{\max}\mathbf{e}, \eta\}\ll0$, there exists $t_{2}>0$ such that

106 $$ u^{\mathrm{I}}(t)\geq\geq\bigl(L_{2}^{\mathrm{I}} \bigr)^{-1}\min\{f_{\max}\mathbf{e}, \eta\} -\varepsilon \mathbf{e},\quad t\geq t_{2}. $$

Now, it follows from (103) and (106) that

107 $$\begin{aligned} \dot{\tilde{S}}_{2}^{\mathrm{I}}(t)+L_{2}^{\mathrm{I}} \tilde{S}_{2}^{\mathrm{I}}(t)\geq\geq \bigl(L_{2}^{\mathrm{I}} \bigr)^{-1}\min\{f_{\max}\mathbf{e}, \eta\}-\varepsilon\mathbf {e},\quad t\geq t_{2}, \end{aligned}$$

and hence,

108 $$\begin{aligned} \tilde{S}_{2}^{\mathrm{I}}(t)\geq\geq{}&\bigl( \tilde{S}_{20}^{\mathrm{I}}-\bigl(L_{2}^{\mathrm{I}} \bigr)^{-1}\bigl(\bigl(L_{2}^{\mathrm{I}} \bigr)^{-1}\min\{f_{\max}\mathbf{e}, \eta\}-\varepsilon \mathbf{e}\bigr)\bigr)e^{-L_{2}^{\mathrm{I}}t} \\ &{}+\bigl(L_{2}^{\mathrm{I}}\bigr)^{-1}\bigl( \bigl(L_{2}^{\mathrm{I}}\bigr)^{-1}\min\{f_{\max} \mathbf{e}, \eta\}-\varepsilon\mathbf{e}\bigr),\quad t\geq t_{2}. \end{aligned}$$

Thus, it follows from (108) that $\tilde{S}_{2}^{\mathrm{I}}(t)\geq\geq(L_{2}^{\mathrm{I}})^{-1}((L_{2}^{\mathrm{I}})^{-1}\min\{f_{\max}\mathbf {e}, \eta\}-\varepsilon\mathbf{e})$ as $t\to\infty$, and since $\tilde {S}_{20}^{\mathrm{I}}-(L_{2}^{\mathrm{I}})^{-1}((L_{2}^{\mathrm{I}})^{-1}\min\{f_{\max }\mathbf{e}, \eta\}-\varepsilon\mathbf{e})\ll0$, there exist $t_{3}>t_{2}>0$ such that

109 $$ \tilde{S}_{2}^{\mathrm{I}}(t)\geq\geq\bigl(L_{2}^{\mathrm{I}} \bigr)^{-1}\bigl[\bigl(L_{2}^{\mathrm{I}} \bigr)^{-1}\min\{f_{\max}\mathbf{e}, \eta\}-\varepsilon\mathbf {e}\bigr]-\varepsilon\mathbf{e},\quad t\geq t_{3}. $$

Next, since $A_{1}^{\mathrm{EI}}\leq\leq0$ and $\tilde{S}_{1}^{\mathrm{I}}(t)\geq \geq0$, $t\geq0$, and $f_{i}(x)$, $x\in\mathbb{R}$, $i=1,\ldots,n_{\mathrm{E}}$, are nondecreasing functions, it follows from (74) that

110 $$\begin{aligned} &\ddot{S}^{\mathrm{E}}(t)\leq\leq-2L^{\mathrm{E}}\dot{S}^{\mathrm{E}}(t)- \bigl(L^{\mathrm{E}}\bigr)^{2}S^{\mathrm{E}}(t)+f^{\mathrm{E}} \bigl(A^{\mathrm{EE}}S^{\mathrm{E}}(t)+A_{2}^{\mathrm{EI}} \tilde {S}_{2}^{\mathrm{I}}+\tilde{v}_{\mathrm{th}}^{\mathrm{E}}(t) \bigr), \\ &\quad \quad \quad \quad \quad \quad \quad \quad \quad \quad \quad \quad \quad S^{\mathrm{E}}(0)=S^{\mathrm{E}}_{0}, \quad \dot {S}^{\mathrm{E}}(0)=\dot{S}^{\mathrm{E}}_{0}, \quad t\geq0. \end{aligned}$$

Now, since $A^{\mathrm{EE}}\geq\geq0$ and $A_{2}^{\mathrm{EI}}\leq\leq0$, it follows from (101) and (109) that

111 $$\begin{aligned} &A^{\mathrm{EE}}S^{\mathrm{E}}(t)+A_{2}^{\mathrm{EI}} \tilde{S}_{2}^{\mathrm{I}}(t)+\tilde {v}_{\mathrm{th}}^{\mathrm{E}}(t) \\ &\quad \leq\leq A^{\mathrm{EE}}\max\bigl\{ S^{\mathrm{E}}_{0}, \bigl(L^{\mathrm{E}}\bigr)^{-1} \max\bigl\{ \dot{S}_{0}^{\mathrm{E}}+L^{\mathrm{E}}S_{0}^{\mathrm{E}}, f_{\max}\bigl(L^{\mathrm{E}}\bigr)^{-1}\mathbf{e}\bigr\} \bigr\} \\ &\qquad\quad {}+A_{2}^{\mathrm{EI}}\bigl[\bigl(L_{2}^{\mathrm{I}} \bigr)^{-1}\bigl[\bigl(L_{2}^{\mathrm{I}} \bigr)^{-1}\min\{f_{\max}\mathbf{e}, \eta\}-\varepsilon\mathbf {e}\bigr]- \varepsilon\mathbf{e}\bigr]+\beta \\ &\quad \leq\leq 0,\quad t\geq t_{3}. \end{aligned}$$

Finally, it follows from (110) and (111) that

112 $$\begin{aligned} &\ddot{S}^{\mathrm{E}}(t)\leq\leq-2L^{\mathrm{E}}\dot{S}^{\mathrm{E}}(t)- \bigl(L^{\mathrm{E}}\bigr)^{2}S^{\mathrm{E}}(t), \\ &\quad \quad \quad \quad\quad \quad \quad S^{\mathrm{E}}(0)=S^{\mathrm{E}}_{0}, \quad \dot{S}^{\mathrm{E}}(0)= \dot{S}^{\mathrm{E}}_{0},\quad t\geq t_{3}, \end{aligned}$$

which implies that $S^{\mathrm{E}}(t)\to0$ as $t\to\infty$, and hence, (74), (75), and (76) is globally asymptotically partially synchronized. □

Condition (98) implies that all the excitatory neurons receive inhibitory inputs from the inhibitory neurons that themselves do not receive inhibitory inputs. As in the case of the simpler neural population model discussed in Sect. 4, if only a subset of excitatory neurons receive the inhibitory inputs from the inhibitory neurons that themselves do not receive inhibitory inputs, then only the synaptic drives of this subset of excitatory neurons will converge to zero as the inhibitory time constants increase.

## A Second-Order Mean Excitatory and Inhibitory Synaptic Drive Model

In this section, we reduce the model given in (69) and (70) to a mean excitatory and mean inhibitory model. Consider (69) and (70) with continuously differentiable $f_{i}(\cdot)=f(\cdot)$, $B_{i}^{\mathrm{E}}=B_{i}^{\mathrm{I}}=1$, $\lambda_{i}^{\mathrm{E}}=\lambda^{\mathrm{E}}$, and $\lambda _{i}^{\mathrm{I}}=\lambda^{\mathrm{I}}$. In this case, (69) and (70) become

113 $$\begin{aligned} \frac{\mathrm{d}^{2}S_{i}^{\mathrm{E}}(t)}{\mathrm{d}t^{2}}={}&{-}\frac{2}{\lambda^{\mathrm{E}}}\frac{\mathrm{d}S_{i}^{\mathrm{E}}(t)}{\mathrm{d}t}- \biggl(\frac{1}{\lambda^{\mathrm{E}}} \biggr)^{2}S_{i}^{\mathrm{E}}(t) \\ &{}+f \Biggl(\sum_{j=1}^{n_{\mathrm{E}}}A_{ij}^{\mathrm{EE}}S_{j}^{\mathrm{E}}(t)+ \sum_{k=1}^{n_{\mathrm{I}}}A_{ik}^{\mathrm{EI}}S_{k}^{\mathrm{I}}(t) +v_{\mathrm{th}i}^{\mathrm{E}}(t) \Biggr),\quad i=1,\ldots,n_{\mathrm{E}}, \end{aligned}$$

114 $$\begin{aligned} \frac{\mathrm{d}^{2}S_{i}^{\mathrm{I}}(t)}{\mathrm{d}t^{2}}={}&{-}\frac{2}{\lambda^{\mathrm{I}}} \frac{\mathrm{d}S_{i}^{\mathrm{I}}(t)}{\mathrm{d}t}- \biggl(\frac{1}{\lambda^{\mathrm{I}}} \biggr)^{2}S_{i}^{\mathrm{I}}(t) \\ &{}+f \Biggl(\sum_{j=1}^{n_{\mathrm{E}}}A_{ij}^{\mathrm{IE}}S_{j}^{\mathrm{E}}(t)+ \sum_{k=1}^{n_{\mathrm{I}}}A_{ik}^{\mathrm{II}}S_{k}^{\mathrm{I}}(t) +v_{\mathrm{th}i}^{\mathrm{I}}(t) \Biggr),\quad i=1,\ldots,n_{\mathrm{I}}, \end{aligned}$$

where $A_{ii}^{\mathrm{EE}}=A_{ii}^{\mathrm{II}}=0$. Next, let $A_{ij}^{\mathrm{EE}}=\overline{A}^{\mathrm{EE}}+\Delta_{ij}^{\mathrm{EE}}$, $A_{ij}^{\mathrm{EI}}=\overline{A}^{\mathrm{EI}}+\Delta_{ij}^{\mathrm{EI}}$, $A_{ij}^{\mathrm{IE}}=\overline{A}^{\mathrm{IE}}+\Delta_{ij}^{\mathrm{IE}}$, and $A_{ij}^{\mathrm{II}}=\overline{A}^{\mathrm{II}}+\Delta_{ij}^{\mathrm{II}}$, where $\overline {A}^{\mathrm{XY}}\triangleq\frac{1}{n_{\mathrm{X}}n_{\mathrm{Y}}}\sum_{i=1}^{n_{\mathrm{X}}}\sum_{j=1}^{n_{\mathrm{Y}}}A_{ij}^{\mathrm{XY}}$, X, $\mathrm{Y}\in\{\mathrm{E}, \mathrm{I}\}$, denote mean and $\Delta_{ij}^{\mathrm{XY}}$, X, $\mathrm{Y}\in\{\mathrm{E}, \mathrm{I}\}$, are deviations from the mean. In this case, it follows that

115 $$ \sum_{i=1}^{n_{\mathrm{E}}}\sum _{j=1}^{n_{\mathrm{E}}}\Delta_{ij}^{\mathrm{EE}}= \sum_{i=1}^{n_{\mathrm{E}}}\sum _{j=1}^{n_{\mathrm{I}}}\Delta_{ij}^{\mathrm{EI}}= \sum_{i=1}^{n_{\mathrm{I}}}\sum _{j=1}^{n_{\mathrm{E}}}\Delta_{ij}^{\mathrm{IE}}= \sum_{i=1}^{n_{\mathrm{I}}}\sum _{j=1}^{n_{\mathrm{I}}}\Delta_{ij}^{\mathrm{II}}=0. $$

Using the average and perturbed expression for $A_{ij}^{\mathrm{XY}}$, X, $\mathrm{Y}\in\{\mathrm{E},\mathrm{I}\}$, (113) and (114) can be written as

116 $$\begin{aligned} \frac{\mathrm{d}^{2}S_{i}^{\mathrm{E}}(t)}{\mathrm{d}t^{2}}={}&f \Biggl(n_{\mathrm{E}}\overline {A}^{\mathrm{EE}}\overline{S}^{\mathrm{E}}(t)+\sum _{j=1}^{n_{\mathrm{E}}}\Delta _{ij}^{\mathrm{EE}}S_{j}^{\mathrm{E}}(t)+n_{\mathrm{I}} \overline{A}^{\mathrm{EI}}\overline {S}^{\mathrm{I}}(t)+\sum _{k=1}^{n_{\mathrm{I}}}\Delta_{ik}^{\mathrm{EI}}S_{k}^{\mathrm{I}}(t) \\ &{}+v_{\mathrm{th}i}^{\mathrm{E}}(t) \Biggr)-\frac{2}{\lambda^{\mathrm{E}}} \frac {\mathrm{d}S_{i}^{\mathrm{E}}(t)}{\mathrm{d}t}- \biggl(\frac{1}{\lambda^{\mathrm{E}}} \biggr)^{2}S_{i}^{\mathrm{E}}(t),\quad i=1,\ldots,n_{\mathrm{E}}, \end{aligned}$$

117 $$\begin{aligned} \frac{\mathrm{d}^{2}S_{i}^{\mathrm{I}}(t)}{\mathrm{d}t^{2}}={}&f \Biggl(n_{\mathrm{E}}\overline {A}^{\mathrm{IE}}\overline{S}^{\mathrm{E}}(t)+\sum _{j=1}^{n_{\mathrm{E}}}\Delta _{ij}^{\mathrm{IE}}S_{j}^{\mathrm{E}}(t)+n_{\mathrm{I}} \overline{A}^{\mathrm{II}}\overline {S}^{\mathrm{I}}(t)+\sum _{k=1}^{n_{\mathrm{I}}}\Delta_{ik}^{\mathrm{II}}S_{k}^{\mathrm{I}}(t) \\ &{}+v_{\mathrm{th}i}^{\mathrm{I}}(t) \Biggr)-\frac{2}{\lambda^{\mathrm{I}}} \frac {\mathrm{d}S_{i}^{\mathrm{I}}(t)}{\mathrm{d}t}- \biggl(\frac{1}{\lambda^{\mathrm{I}}} \biggr)^{2}S_{i}^{\mathrm{I}}(t),\quad i=1,\ldots,n_{\mathrm{I}}, \end{aligned}$$

where $\overline{S}^{\mathrm{E}}(t)\triangleq\frac{1}{n_{\mathrm{E}}}\sum_{j=1}^{n_{\mathrm{E}}}S_{j}^{\mathrm{E}}(t)$ and $\overline{S}^{\mathrm{I}}(t)\triangleq\frac{1}{n_{\mathrm{I}}}\sum_{j=1}^{n_{\mathrm{I}}}S_{j}^{\mathrm{I}}(t)$ denote the mean excitatory synaptic drive and mean inhibitory synaptic drive in dimensionless units, respectively. Now, defining $\delta_{i}^{\mathrm{E}}(t)\triangleq S_{i}^{\mathrm{E}}(t)-\overline{S}^{\mathrm{E}}(t)$ and $\delta_{i}^{\mathrm{I}}(t)\triangleq S_{i}^{\mathrm{I}}(t)-\overline{S}^{\mathrm{I}}(t)$, where $\delta_{i}^{\mathrm{E}}(t)$ and $\delta_{i}^{\mathrm{I}}(t)$ are deviations from the mean, (116) and (117) become

118 $$\begin{aligned} \frac{\mathrm{d}^{2}S_{i}^{\mathrm{E}}(t)}{\mathrm{d}t^{2}}={}&f \Biggl(n_{\mathrm{E}}\overline {A}^{\mathrm{EE}}\overline{S}^{\mathrm{E}}(t)+\overline{S}^{\mathrm{E}}(t) \sum_{j=1}^{n_{\mathrm{E}}}\Delta_{ij}^{\mathrm{EE}}+ \sum_{j=1}^{n_{\mathrm{E}}}\Delta _{ij}^{\mathrm{EE}} \delta_{j}^{\mathrm{E}}(t) +n_{\mathrm{I}}\overline{A}^{\mathrm{EI}} \overline{S}^{\mathrm{I}}(t) \\ &{}+\overline{S}^{\mathrm{I}}(t)\sum_{k=1}^{n_{\mathrm{I}}} \Delta_{ik}^{\mathrm{EI}}+\sum_{k=1}^{n_{\mathrm{I}}} \Delta_{ik}^{\mathrm{EI}}\delta_{k}^{\mathrm{I}}(t)+v_{\mathrm{th}i}^{\mathrm{E}}(t) \Biggr)-\frac{2}{\lambda^{\mathrm{E}}}\frac {\mathrm{d}S_{i}^{\mathrm{E}}(t)}{\mathrm{d}t} \\ &{}- \biggl(\frac{1}{\lambda^{\mathrm{E}}} \biggr)^{2}S_{i}^{\mathrm{E}}(t),\quad i=1,\ldots,n_{\mathrm{E}}, \end{aligned}$$

119 $$\begin{aligned} \frac{\mathrm{d}^{2}S_{i}^{\mathrm{I}}(t)}{\mathrm{d}t^{2}}={}&f \Biggl(n_{\mathrm{E}}\overline {A}^{\mathrm{IE}}\overline{S}^{\mathrm{E}}(t) +\overline{S}^{\mathrm{E}}(t) \sum_{j=1}^{n_{\mathrm{E}}}\Delta_{ij}^{\mathrm{IE}}+ \sum_{j=1}^{n_{\mathrm{E}}}\Delta_{ij}^{\mathrm{IE}} \delta_{j}^{\mathrm{E}}(t)+n_{\mathrm{I}}\overline{A}^{\mathrm{II}} \overline{S}^{\mathrm{I}}(t) \\ &{}+\overline{S}^{\mathrm{I}}(t)\sum_{k=1}^{n_{\mathrm{I}}} \Delta_{ik}^{\mathrm{II}}+\sum_{k=1}^{n_{\mathrm{I}}} \Delta_{ik}^{\mathrm{II}}\delta_{k}^{\mathrm{I}}(t)+v_{\mathrm{th}i}^{\mathrm{I}}(t) \Biggr)-\frac{2}{\lambda^{\mathrm{I}}}\frac {\mathrm{d}S_{i}^{\mathrm{I}}(t)}{\mathrm{d}t} \\ &{}- \biggl(\frac{1}{\lambda^{\mathrm{I}}} \biggr)^{2}S_{i}^{\mathrm{I}}(t),\quad i=1,\ldots,n_{\mathrm{I}}. \end{aligned}$$

Next, assume that all terms with a factor $\Delta_{ij}^{\mathrm{XY}}$, X, $\mathrm{Y}\in\{\mathrm{E},\mathrm{I}\}$, $i=1,\ldots,n_{\mathrm{X}}$ and $j=1,\ldots,n_{\mathrm{Y}}$, in (118) and (119) are small relative to the remaining terms in $f(\cdot)$. Then a first-order expansion of (118) and (119) gives

120 $$\begin{aligned} \frac{\mathrm{d}^{2}S_{i}^{\mathrm{E}}(t)}{\mathrm{d}t^{2}}={}&f\bigl(n_{\mathrm{E}}\overline {A}^{\mathrm{EE}}\overline{S}^{\mathrm{E}}(t)+n_{\mathrm{I}} \overline{A}^{\mathrm{EI}}\overline{S}^{\mathrm{I}}(t)+v_{\mathrm{th}i}^{\mathrm{E}}(t) \bigr)+f'\bigl(n_{\mathrm{E}}\overline{A}^{\mathrm{EE}} \overline{S}^{\mathrm{E}}(t) \\ &{}+n_{\mathrm{I}}\overline{A}^{\mathrm{EI}}\overline{S}^{\mathrm{I}}(t)+v_{\mathrm{th}i}^{\mathrm{E}}(t) \bigr) \Biggl[\overline{S}^{\mathrm{E}}(t)\sum_{j=1}^{n_{\mathrm{E}}} \Delta_{ij}^{\mathrm{EE}}+\sum_{j=1}^{n_{\mathrm{E}}} \Delta_{ij}^{\mathrm{EE}}\delta _{j}^{\mathrm{E}}(t) \\ &{}+\overline{S}^{\mathrm{I}}(t)\sum_{k=1}^{n_{\mathrm{I}}} \Delta_{ik}^{\mathrm{EI}}+\sum_{k=1}^{n_{\mathrm{I}}} \Delta_{ik}^{\mathrm{EI}}\delta_{k}^{\mathrm{I}}(t) \Biggr]-\frac{2}{\lambda^{\mathrm{E}}}\frac{\mathrm{d}S_{i}^{\mathrm{E}}(t)}{\mathrm{d}t}- \biggl(\frac{1}{\lambda^{\mathrm{E}}} \biggr)^{2}S_{i}^{\mathrm{E}}(t), \\ &{}\quad \quad \quad \quad \quad \quad \quad \quad \quad \quad \quad \quad \quad \quad \quad \quad \quad i=1,\ldots ,n_{\mathrm{E}}, \end{aligned}$$

121 $$\begin{aligned} \frac{\mathrm{d}^{2}S_{i}^{\mathrm{I}}(t)}{\mathrm{d}t^{2}}={}&f\bigl(n_{\mathrm{E}}\overline {A}^{\mathrm{IE}}\overline{S}^{\mathrm{E}}(t) +n_{\mathrm{I}} \overline{A}^{\mathrm{II}}\overline{S}^{\mathrm{I}}(t)+v_{\mathrm{th}i}^{\mathrm{I}}(t) \bigr)+f'\bigl(n_{\mathrm{E}}\overline{A}^{\mathrm{IE}} \overline{S}^{\mathrm{E}}(t) \\ &{}+n_{\mathrm{I}}\overline{A}^{\mathrm{II}}\overline{S}^{\mathrm{I}}(t)+v_{\mathrm{th}i}^{\mathrm{I}}(t) \bigr) \Biggl[ \overline{S}^{\mathrm{E}}(t)\sum_{j=1}^{n_{\mathrm{E}}} \Delta_{ij}^{\mathrm{IE}}+\sum_{j=1}^{n_{\mathrm{E}}} \Delta_{ij}^{\mathrm{IE}}\delta _{j}^{\mathrm{E}}(t) \\ &{}+\overline{S}^{\mathrm{I}}(t)\sum_{k=1}^{n_{\mathrm{I}}} \Delta_{ik}^{\mathrm{II}}+\sum_{k=1}^{n_{\mathrm{I}}} \Delta_{ik}^{\mathrm{II}}\delta_{k}^{\mathrm{I}}(t) \Biggr]-\frac{2}{\lambda^{\mathrm{I}}}\frac{\mathrm{d}S_{i}^{\mathrm{I}}(t)}{\mathrm{d}t}- \biggl(\frac{1}{\lambda^{\mathrm{I}}} \biggr)^{2}S_{i}^{\mathrm{I}}(t), \\ &{}\quad \quad \quad \quad \quad \quad \quad \quad \quad \quad \quad \quad \quad \quad \quad \quad \quad i=1,\ldots ,n_{\mathrm{I}}. \end{aligned}$$

Now, assuming that the higher-order terms can be ignored, (120) and (121) become

122 $$\begin{aligned} \frac{\mathrm{d}^{2}S_{i}^{\mathrm{E}}(t)}{\mathrm{d}t^{2}}={}&f\bigl(n_{\mathrm{E}}\overline {A}^{\mathrm{EE}}\overline{S}^{\mathrm{E}}(t)+n_{\mathrm{I}} \overline{A}^{\mathrm{EI}}\overline{S}^{\mathrm{I}}(t)+v_{\mathrm{th}i}^{\mathrm{E}}(t) \bigr)+f'\bigl(n_{\mathrm{E}}\overline{A}^{\mathrm{EE}} \overline{S}^{\mathrm{E}}(t) \\ &{}+n_{\mathrm{I}}\overline{A}^{\mathrm{EI}}\overline{S}^{\mathrm{I}}(t)+v_{\mathrm{th}i}^{\mathrm{E}}(t) \bigr) \Biggl[\overline{S}^{\mathrm{E}}(t)\sum_{j=1}^{n_{\mathrm{E}}} \Delta_{ij}^{\mathrm{EE}}+\overline{S}^{\mathrm{I}}(t)\sum _{k=1}^{n_{\mathrm{I}}}\Delta_{ik}^{\mathrm{EI}} \Biggr] \\ &{}-\frac{2}{\lambda^{\mathrm{E}}}\frac{\mathrm{d}S_{i}^{\mathrm{E}}(t)}{\mathrm{d}t}- \biggl(\frac{1}{\lambda^{\mathrm{E}}} \biggr)^{2}S_{i}^{\mathrm{E}}(t), \quad i=1, \ldots,n_{\mathrm{E}}, \end{aligned}$$

123 $$\begin{aligned} \frac{\mathrm{d}^{2}S_{i}^{\mathrm{I}}(t)}{\mathrm{d}t^{2}}={}&f\bigl(n_{\mathrm{E}}\overline {A}^{\mathrm{IE}}\overline{S}^{\mathrm{E}}(t) +n_{\mathrm{I}} \overline{A}^{\mathrm{II}}\overline{S}^{\mathrm{I}}(t)+v_{\mathrm{th}i}^{\mathrm{I}}(t) \bigr)+f'\bigl(n_{\mathrm{E}}\overline{A}^{\mathrm{IE}} \overline{S}^{\mathrm{E}}(t) \\ &{}+n_{\mathrm{I}}\overline{A}^{\mathrm{II}}\overline{S}^{\mathrm{I}}(t)+v_{\mathrm{th}i}^{\mathrm{I}}(t) \bigr) \Biggl[ \overline{S}^{\mathrm{E}}(t)\sum_{j=1}^{n_{\mathrm{E}}} \Delta_{ij}^{\mathrm{IE}}+\overline{S}^{\mathrm{I}}(t)\sum _{k=1}^{n_{\mathrm{I}}}\Delta_{ik}^{\mathrm{II}} \Biggr] \\ &{}-\frac{2}{\lambda^{\mathrm{I}}}\frac{\mathrm{d}S_{i}^{\mathrm{I}}(t)}{\mathrm{d}t}- \biggl(\frac{1}{\lambda^{\mathrm{I}}} \biggr)^{2}S_{i}^{\mathrm{I}}(t), \quad i=1, \ldots,n_{\mathrm{I}}. \end{aligned}$$

Finally, summing (122) and (123) over $i=1,\ldots,n_{\mathrm{E}}$ and $i=1,\ldots ,n_{\mathrm{I}}$, dividing by $n_{\mathrm{E}}$, and $n_{\mathrm{I}}$, respectively, using (115), and assuming $v_{\mathrm{th}i}^{\mathrm{E}}(t)=v_{\mathrm{th}}^{\mathrm{E}}$ and $v_{\mathrm{th}i}^{\mathrm{I}}(t)=v_{\mathrm{th}}^{\mathrm{I}}$, $t\geq 0$, it follows that the average excitatory synaptic drive and the average inhibitory synaptic drive are given by

124 $$\begin{aligned} \frac{\mathrm{d}^{2}\overline{S}^{\mathrm{E}}(t)}{\mathrm{d}t^{2}}={}&f\bigl(n_{\mathrm{E}} \overline{A}^{\mathrm{EE}}\overline{S}^{\mathrm{E}}(t)+n_{\mathrm{I}} \overline {A}^{\mathrm{EI}}\overline{S}^{\mathrm{I}}(t)+v_{\mathrm{th}}^{\mathrm{E}}(t) \bigr)-\frac {2}{\lambda^{\mathrm{E}}}\frac{\mathrm{d}\overline{S}^{\mathrm{E}}(t)}{\mathrm{d}t} \\ &{}- \biggl(\frac{1}{\lambda^{\mathrm{E}}} \biggr)^{2}\overline{S}^{\mathrm{E}}(t),\quad \overline{S}^{\mathrm{E}}(0)=\overline{S}^{\mathrm{E}}_{0},\quad \dot {\overline{S}}^{\mathrm{E}}(0)=\dot{\overline{S}}^{\mathrm{E}}_{0},\quad t\geq0, \end{aligned}$$

125 $$\begin{aligned} \frac{\mathrm{d}^{2}\overline{S}^{\mathrm{I}}(t)}{\mathrm{d}t^{2}}={}&f\bigl(n_{\mathrm{E}} \overline{A}^{\mathrm{IE}}\overline{S}^{\mathrm{E}}(t) +n_{\mathrm{I}} \overline{A}^{\mathrm{II}}\overline{S}^{\mathrm{I}}(t)+v_{\mathrm{th}}^{\mathrm{I}}(t) \bigr)-\frac{2}{\lambda^{\mathrm{I}}}\frac{\mathrm{d}\overline {S}^{\mathrm{I}}(t)}{\mathrm{d}t} \\ &{}- \biggl(\frac{1}{\lambda^{\mathrm{I}}} \biggr)^{2}\overline{S}^{\mathrm{I}}(t),\quad \overline{S}^{\mathrm{I}}(0)=\overline{S}^{\mathrm{I}}_{0},\quad \dot {\overline{S}}^{\mathrm{I}}(0)=\dot{\overline{S}}^{\mathrm{I}}_{0}. \end{aligned}$$

A similar analysis to the analysis provided in Sect. 3 can be carried out for the coupled second-order differential equations (124) and (125). This can allow us to qualitatively study the abrupt transition from consciousness to unconsciousness as well as the biphasic response during induction of anesthesia. Initial quantitative numerical experiments of this model are presented in Sect. 7.

## Illustrative Numerical Simulations of the Neural Field Model

In this section, we use the different analysis results developed in the paper to present several numerical experiments that illustrate the qualitative behavior of the proposed firing-rate models.

### Two-Class Mean Excitatory and Mean Inhibitory Synaptic Drive Model

**Fig. 3:**
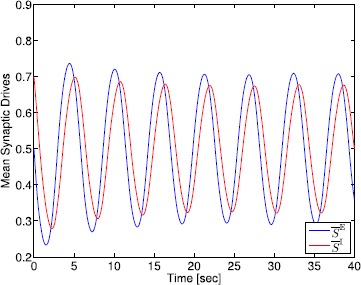
$\overline{S}^{\mathrm{E}}(t)$ and $\overline{S}^{\mathrm{I}}(t)$ versus time for $a=10$, $b=9$, $c=6$, $d=1$, $v_{\mathrm{th}}^{\mathrm{E}}=-0.5$, $v_{\mathrm{th}}^{\mathrm{I}}=-2.5$, $\lambda^{\mathrm{E}}=1$, $\lambda^{\mathrm{I}}=1$, $f_{\max}=1$, and $\gamma=1$ with $\overline{S}^{\mathrm{E}}(0)=0.5$ and $\overline{S}^{\mathrm{I}}(0)=0.7$

**Fig. 4:**
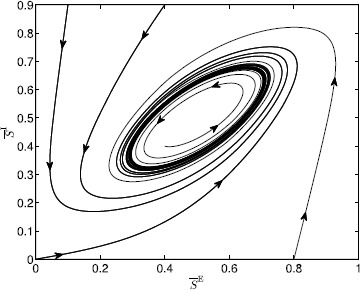
Phase portrait of (11) and (12) for $a=10$, $b=9$, $c=6$, $d=1$, $v_{\mathrm{th}}^{\mathrm{E}}=-0.5$, $v_{\mathrm{th}}^{\mathrm{I}}=-2.5$, $\lambda^{\mathrm{E}}=1$, $\lambda^{\mathrm{I}}=1$, $f_{\max}=1$, and $\gamma=1$

First, we consider the two-class mean field model (11) and (12) with a single equilibrium point. Figures 3 and 4 show the case when the equilibrium point is unstable, and hence, the system possesses a stable limit cycle.

**Fig. 5:**
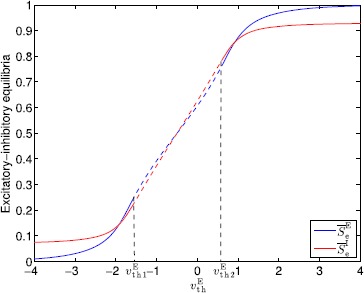
Bifurcation diagram of (11) and (12) for $a=10$, $b=9$, $c=6$, $d=1$, $v_{\mathrm{th}}^{\mathrm{I}}=-2.5$, $\lambda^{\mathrm{E}}=1$, $\lambda^{\mathrm{I}}=1$, $f_{\max}=1$, and $\gamma=1$ with $v_{\mathrm{th}}^{\mathrm{E}}$ as a bifurcation parameter. The *solid line* represents asymptotically stable equilibrium point, whereas the *dashed line* represents unstable equilibrium point. A supercritical Hopf bifurcation occurs at $v^{\mathrm{E}}_{\mathrm{th}}=-1.6$ where the single asymptotically stable equilibrium point becomes unstable and gives rise to a stable limit cycle. At $v^{\mathrm{E}}_{\mathrm{th}}=0.6$, the unstable equilibrium point reverts to an asymptotically stable equilibrium point and the stable limit cycle disappears

**Fig. 6:**
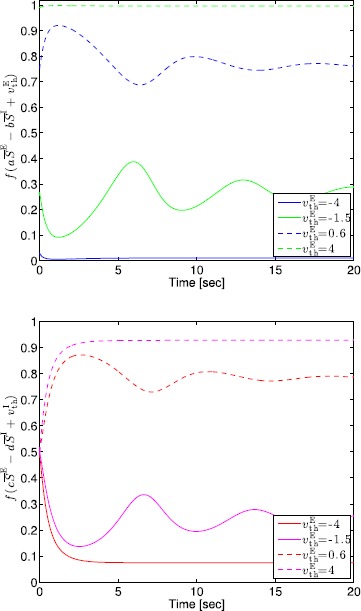
Mean firing rates for $a=10$, $b=9$, $c=6$, $d=1$, $v_{\mathrm{th}}^{\mathrm{I}}=-2.5$, $\lambda^{\mathrm{E}}=1$, $\lambda^{\mathrm{I}}=1$, $f_{\max }=1$, and $\gamma=1$ with $\overline{S}^{\mathrm{E}}(0)=0.5$ and $\overline {S}^{\mathrm{I}}(0)=0.5$ and with different values of $v^{\mathrm{E}}_{\mathrm{th}}$

**Fig. 7:**
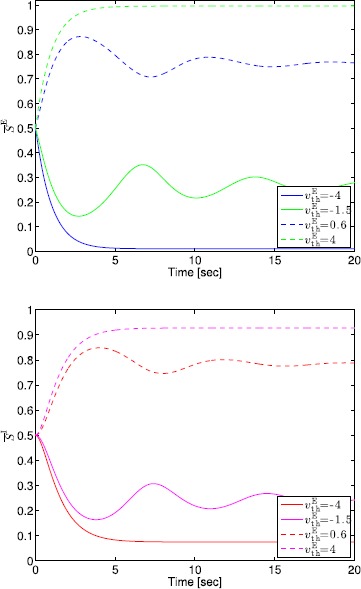
Mean synaptic drives for $a=10$, $b=9$, $c=6$, $d=1$, $v_{\mathrm{th}}^{\mathrm{I}}=-2.5$, $\lambda^{\mathrm{E}}=1$, $\lambda^{\mathrm{I}}=1$, $f_{\max }=1$, and $\gamma=1$ with $\overline{S}^{\mathrm{E}}(0)=0.5$ and $\overline {S}^{\mathrm{I}}(0)=0.5$ and with different values of $v^{\mathrm{E}}_{\mathrm{th}}$

Next, we illustrate the effect of the excitatory sensory input $v^{\mathrm{E}}_{\mathrm{th}}$ on the mean excitatory and mean inhibitory synaptic drives. Here, we fix the parameters to be as in the simulation shown in Fig. 3 and vary $v^{\mathrm{E}}_{\mathrm{th}}$. It can be seen from Fig. 5 that, for the value of $v_{\mathrm{th}}^{\mathrm{E}}$ below $v_{\mathrm{th1}}^{\mathrm{E}}=-1.6$, (11) and (12) have exactly one asymptotically stable equilibrium point. As $v^{\mathrm{E}}_{\mathrm{th}}$ increases to $v^{\mathrm{E}}_{\mathrm{th2}}=-1.6$, the equilibrium point becomes unstable. At this point, a supercritical Hopf bifurcation occurs, giving rise to a stable limit cycle. As $v^{\mathrm{E}}_{\mathrm{th}}$ increases beyond $v^{\mathrm{E}}_{\mathrm{th2}}$, the unstable equilibrium point reverts to an asymptotically stable equilibrium point and the stable limit cycle disappears. The value of $\overline{S}^{\mathrm{E}}_{\mathrm{e}}$ and $\overline{S}^{\mathrm{I}}_{\mathrm{e}}$ increases as $v^{\mathrm{E}}_{\mathrm{th}}$ increases; see Fig. 5. The effect of $v^{\mathrm{E}}_{\mathrm{th}}$ on the mean firing rates and mean synaptic drives of the excitatory and the inhibitory neurons is shown in Figs. 6 and 7, respectively. As $v^{\mathrm{E}}_{\mathrm{th}}$ increases, the mean firing rates and mean synaptic drives for the excitatory and the inhibitory neurons increase for the chosen parameter values.

**Fig. 8:**
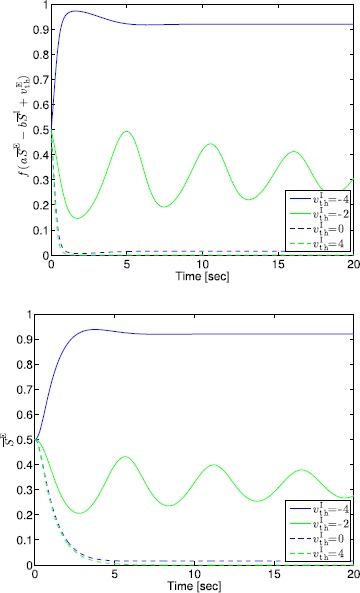
Mean excitatory firing rates and mean excitatory synaptic drives for $a=10$, $b=9$, $c=6$, $d=1$, $v_{\mathrm{th}}^{\mathrm{E}}=-0.5$, $\lambda^{\mathrm{E}}=1$, $\lambda^{\mathrm{I}}=1$, $f_{\max}=1$, and $\gamma=1$ with $\overline{S}^{\mathrm{E}}(0)=0.5$ and $\overline{S}^{\mathrm{I}}(0)=0.5$ and with different values of $v^{\mathrm{I}}_{\mathrm{th}}$

The input voltage $v^{\mathrm{I}}_{\mathrm{th}}$ has a negative effect on the mean excitatory firing rate and synaptic drive. As can be seen from Fig. 8, when $v^{\mathrm{I}}_{\mathrm{th}}$ increases the mean excitatory firing rate and synaptic drive decrease for the chosen parameter values.

**Fig. 9:**
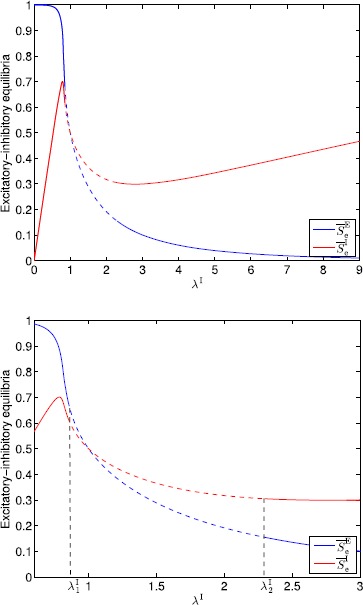
Bifurcation diagram of (11) and (12) for $a=10$, $b=9$, $c=6$, $d=1$, $v_{\mathrm{th}}^{\mathrm{E}}=-0.5$, $v_{\mathrm{th}}^{\mathrm{I}}=-2.5$, $\lambda^{\mathrm{E}}=1$, $f_{\max}=1$, and $\gamma=1$ with $\lambda^{\mathrm{I}}$ as a bifurcation parameter. The *solid line* represents asymptotically stable equilibrium point, whereas the *dashed line* represents unstable equilibrium point. A supercritical Hopf bifurcation occurs at $\lambda ^{\mathrm{I}}_{1}=0.85$ where the single asymptotically stable equilibrium point becomes unstable and gives rise to a stable limit cycle. At $\lambda^{\mathrm{I}}_{2}=2.3$, the unstable equilibrium point reverts to an asymptotically stable equilibrium point and the stable limit cycle disappears

Finally, we relate the effect of an anesthetic drug, corresponding to an increase in the inhibitory time constant $\lambda^{\mathrm{I}}$, to the mean excitatory and mean inhibitory synaptic drives. Here, we fix the parameters to be as in the simulation shown in Fig. 3 and vary $\lambda^{\mathrm{I}}$, which corresponds to concentration variations in the anesthetic drug. As can be seen in Fig. 9, for the value of $\lambda ^{\mathrm{I}}$ below $\lambda^{\mathrm{I}}_{1}=0.85$, (11) and (12) have exactly one asymptotically stable equilibrium point. As $\lambda^{\mathrm{I}}$ increases to $\lambda^{\mathrm{I}}_{1}$, the equilibrium point becomes unstable. At this point, a supercritical Hopf bifurcation occurs, giving rise to a stable limit cycle. As $\lambda^{\mathrm{I}}$ increases beyond $\lambda^{\mathrm{I}}_{2}=2.3$, the unstable equilibrium point reverts to an asymptotically stable equilibrium point and the stable limit cycle disappears. The value of $\overline{S}^{\mathrm{E}}_{\mathrm{e}}$ decreases as $\lambda^{\mathrm{I}}$ increases and approaches zero as $\lambda^{\mathrm{I}}$ becomes large; see Fig. 9.

**Fig. 10:**
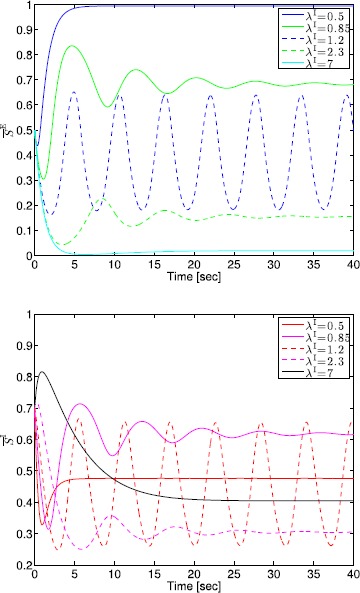
Mean synaptic drives for $a=10$, $b=9$, $c=6$, $d=1$, $v_{\mathrm{th}}^{\mathrm{E}}=-0.5$, $v_{\mathrm{th}}^{\mathrm{I}}=-2.5$, $\lambda^{\mathrm{E}}=1$, $f_{\max}=1$, and $\gamma=1$ with $\overline{S}^{\mathrm{E}}(0)=0.5$ and $\overline{S}^{\mathrm{I}}(0)=0.7$ and with different values of $\lambda ^{\mathrm{I}}$

The mean excitatory synaptic drive $\overline{S}^{\mathrm{E}}(t)$, $t\geq 0$, also decreases as $\lambda^{\mathrm{I}}$ increases and approaches zero with increasing $\lambda^{\mathrm{I}}$; see Fig. 10. The effect of anesthetic drug can be explained by observing that if $\lambda^{\mathrm{I}}$ is below $\lambda^{\mathrm{I}}_{1}$, corresponding to no drug effect, then the mean excitatory synaptic drive is at a high level, which corresponds to an awake state. The range of drug concentrations where we cannot predict with certainty whether the patient will respond to noxious stimuli or not could certainly be explained by stochastic variation in $v^{\mathrm{E}}_{\mathrm{th}}$ and $v_{\mathrm{th}}^{\mathrm{I}}$; the postulated inputs from pain, sensorimotor, and proprioceptive sensors, or the reticular activating system.

However, an alternative explanation is that during the induction of anesthesia, the patient may or may not respond to a certain range of anesthetic drug concentration. This is captured in our model by the existence of limit cycle for a range of $\lambda^{\mathrm{I}}$ between $\lambda^{\mathrm{I}}_{1}$ and $\lambda^{\mathrm{I}}_{2}$. In this case, the mean excitatory synaptic drive has a maximum and a minimum value as can be seen in Fig. 10. The probability of observing the response of the patient depends on the time in the cycle at which the observation is made. The patient will respond if the observation occurs at the time when the mean excitatory synaptic drive is at its peak and will not respond if the mean excitatory synaptic drive is at its nadir. If the concentration of the anesthetic drug is further increased, corresponding to an increase in $\lambda^{\mathrm{I}}$, then the mean excitatory synaptic drive approaches zero as shown in Fig. 10. At this point, the patient is deeply sedated and will not respond.

### Partial Synchronization of Synaptic Drive Dynamics

**Fig. 11:**
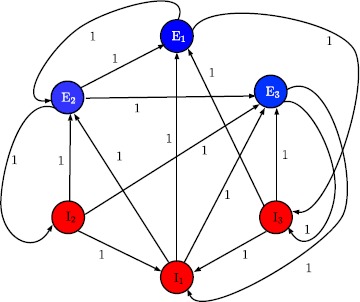
A population of three excitatory neurons $\mathrm{E}_{1}$–$\mathrm{E}_{3}$ and three inhibitory neurons $\mathrm{I}_{1}$–$\mathrm{I}_{3}$. The synaptic weights shown on the *arcs* represent the coupling strength of the neural interconnection

For our first example, we demonstrate partial synchronization for the model given by (34) and (35) with three excitatory neurons $\mathrm{E}_{1}$–$\mathrm{E}_{3}$ and three inhibitory neurons $\mathrm{I_{1}}$–$\mathrm{I_{3}}$ as shown in Fig. 11. The neural connectivity matrix *A* is given by

$$\begin{aligned} A&= \small{\begin{bmatrix} 0 &1 &0 &-1 &0 &-1 \\ 1 &0 &0 &-1 &-1 &0\\ 0 &1 &0 &-1 &-1 &-1 \\ 0 &0 &1 &0 &-1 &-1\\ 0 &1 &0 &0 &0 &0 \\ 1 &0 &1 &0 &0 &0 \end{bmatrix}} , \end{aligned}$$

which implies that the two inhibitory neurons $\mathrm{I}_{2}$ and $\mathrm{I}_{3}$ do not receive inhibitory inputs. Here, we assume that all the excitatory neurons have the same time constant $\lambda^{\mathrm{E}}=0.05\ \mathrm{s}$ and all the inhibitory neurons have the same prolonged time constant $\lambda^{\mathrm{I}}=0.5\ \mathrm{s}$. Furthermore, we assume that $\tilde{v}^{\mathrm{E}}_{\mathrm{th}}=[0.02, 0.02, 0.02]^{\mathrm{T}}$, $\tilde {v}^{\mathrm{I}}_{\mathrm{th}}=[0.02, 0.3, 0.5]^{\mathrm{T}}$, and the activation functions $f_{i}(\cdot)$, $i=1,2,\ldots,6$, are given by (9).

We use the MATLAB^®^ toolbox YALMIP for solving the linear matrix inequalities (LMIs) given by (36) and (37). For the chosen values of *A*, $\lambda^{\mathrm{E}}$, and $\lambda^{\mathrm{I}}$, (36) and (37) are satisfied with

$$\begin{aligned} P&= \small{\begin{bmatrix} 5.74&0&2.24&-1.13&-1.14&-0.05\\ 0&6.08&0&-1.86&-0.77&-1.85\\ 2.24&0&6.35&-0.05&-2.27&-2.26\\ -1.13&-1.86&-0.05&40.02&10.25&10.23\\ -1.14&-0.77&-2.27&10.25&48.72&16.42\\ -0.05&-1.85&-2.26&10.23&16.42&48.75 \end{bmatrix}} , \\ Q&= \small{\begin{bmatrix} 55.53&0.05&-0.08&-0.73&-0.8&-0.81\\ 0.05&55.36&0.08&-1.33&-1.13&-1.12\\ -0.08&0.08&55.74&-0.88&-1.38&-1.27\\ -0.73&-1.33&-0.88&63.73&8.4&8.4\\ -0.8&-1.13&-1.38&8.4&65.58&10.26\\ -0.81&-1.12&-1.27&8.4&10.26&65.47 \end{bmatrix}} , \\ R&=\operatorname {diag} \begin{bmatrix} 24.39 &24.55 &15.85 &49.42& 89.76& 91.31 \end{bmatrix}. \end{aligned}$$

**Fig. 12:**
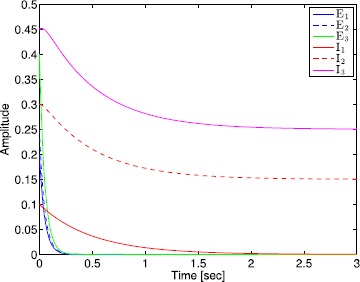
Solutions to (25) and (26) with initial conditions $S^{\mathrm{E}}(0)=[0.2, 0.25, 0.4]^{\mathrm{T}}$, $S^{\mathrm{I}}(0)=[ 0.1, 0.3, 0.45]^{\mathrm{T}}$ for $\lambda^{\mathrm{E}}=0.05\ \mathrm{s}$ and $\lambda^{\mathrm{I}}=0.5\ \mathrm{s}$. The synaptic drive of the excitatory neurons $\mathrm{E}_{1}$ to $\mathrm{E}_{3}$ and the inhibitory neuron $\mathrm{I}_{1}$ converges to zero, whereas the synaptic drive of the inhibitory neurons $\mathrm{I}_{2}$ and $\mathrm{I}_{3}$ that themselves do not receive inhibitory inputs do not converge to zero

Hence, the biological neural network given in (34) and (35) is globally exponentially partially synchronized. As can be seen in Fig. 12, the synaptic drive of the excitatory neurons $\mathrm{E}_{1}$ to $\mathrm{E}_{3}$ and the inhibitory neuron $\mathrm{I}_{1}$ converges to zero, whereas the synaptic drive of the inhibitory neurons $\mathrm{I}_{2}$ and $\mathrm{I}_{3}$ that themselves do not receive inhibitory inputs do not converge to zero.

Next, we increase the number of neurons in the network to twelve with six excitatory neurons $\mathrm{E}_{1}$–$\mathrm{E}_{6}$ and six inhibitory neurons $\mathrm{I}_{1}$–$\mathrm{I}_{6}$. The connections between neurons are also increased as shown in the neural connectivity matrix *A* given by

126 $$\begin{aligned} A= \left[ \small{\textstyle\begin{array}{@{}c@{\quad}c@{\quad}c@{\quad}c@{\quad}c@{\quad}c@{\quad}c@{\quad}c@{\quad}c@{\quad}c@{\quad}c@{\quad}c@{}} 0 & 1 & 1 & 1 & 1 & 1 & -1 & 0 & -1 & -1 & -1 & 0 \\ 1 & 0 & 1 & 1 & 1 & 1 & 0 & -1 & -1 & -1 & 0 & -1 \\ 1 & 1 & 0 & 1 & 1 & 1 & -1 & 0 & -1 & 0 & -1 & -1 \\ 1 & 1 & 1 & 0 & 1 & 1 & 0 & -1 & 0 & -1 & 0 & -1 \\ 1 & 1 & 1 & 1 & 0 & 1 & -1 & 0 & -1 & 0 & -1 & -1 \\ 1 & 1 & 1 & 1 & 1 & 0 & 0 & -1 & 0 & -1 & 0 & -1 \\ 1 & 0 & 1 & 0 & 1 & 0 & 0 & -1 & -1 & 0 & -1 & 0 \\ 0 & 1 & 0 & 1 & 0 & 1 & -1 & 0 & -1 & 0 & -1 & 0 \\ 0 & 0 & 1 & 1 & 1 & 0 & -1 & -1 & 0 & -1 & -1 & 0 \\ 0 & 1 & 0 & 0 & 1 & 1 & 0 & 0 & 0 & 0 & 0 & 0 \\ 1 & 0 & 1 & 1 & 0 & 0 & 0 & 0 & 0 & 0 & 0 & 0 \\ 1 & 1 & 0 & 0 & 0 & 1 & 0 & 0 & 0 & 0 & 0 & 0 \end{array}\displaystyle } \right], \end{aligned}$$

which implies that the three inhibitory neurons $\mathrm{I}_{4}$, $\mathrm{I}_{5}$, and $\mathrm{I}_{6}$ do not receive inhibitory inputs. Here, we assume that all the excitatory neurons have the same time constant $\lambda^{\mathrm{E}}=0.05\ \mathrm{s}$ and all the inhibitory neurons have the same prolonged time constant $\lambda^{\mathrm{I}}=0.35\ \mathrm{s}$. Furthermore, we assume that $\tilde{v}^{\mathrm{E}}_{\mathrm{th}}=[0.02, 0.02, 0.02, 0.02, 0.02, 0.02]^{\mathrm{T}}$, $\tilde{v}^{\mathrm{I}}_{\mathrm{th}}=[0.02, 0.02, 0.02, 0.1, 0.3, 0.5]^{\mathrm{T}}$, and the activation functions $f_{i}(\cdot)$, $i=1,2, \ldots, 12$, are given by (9).

The LMIs given by (36) and (37) are satisfied with

$$\begin{aligned} P&= \left[ \small{\textstyle\begin{array}{@{}c@{\quad}c@{\quad}c@{\quad}c@{\quad}c@{\quad}c@{\quad}c@{\quad}c@{\quad}c@{}} 0.24 & 0.08 & 0.11 & 0.11 & 0.15 & 0.08 & -0.07 & -0.24 & -0.06 \\ 0.08 & 0.2 & 0.07 & 0.11 & 0.08 & 0.11 & -0.16 & -0.08 & -0.12 \\ 0.11 & 0.07 & 0.19 & 0.1 & 0.11 & 0.07 & -0.06 & -0.17 & -0.09 \\ 0.11 & 0.11 & 0.1 & 0.23 & 0.11 & 0.11 & -0.16 & -0.13 & -0.15 \\ 0.15 & 0.08 & 0.11 & 0.11 & 0.24 & 0.08 & -0.07 & -0.24 & -0.06 \\ 0.08 & 0.11 & 0.07 & 0.11 & 0.08 & 0.2 & -0.16 & -0.07 & -0.15 \\ -0.07 & -0.16 & -0.06 & -0.16 & -0.07 & -0.16 & 1.13 & 0.03 & 0.49 \\ -0.24 & -0.08 & -0.17 & -0.13 & -0.24 & -0.07 & 0.03 & 1.42 & 0.13 \\ -0.06 & -0.12 & -0.09 & -0.15 & -0.06 & -0.15 & 0.49 & 0.13 & 1.07 \end{array}\displaystyle }\right] , \\ Q&= \left[ \small{\textstyle\begin{array}{@{}c@{\quad}c@{\quad}c@{\quad}c@{\quad}c@{\quad}c@{\quad}c@{\quad}c@{\quad}c@{}} 1.45&0.08&0.09&0.1&0.11&0.07&-0.13&-0.31&-0.14\\ 0.08&1.41&0.06&0.09&0.08&0.07&-0.18&-0.16&-0.18\\ 0.09&0.06&1.41&0.08&0.09&0.06&-0.12&-0.23&-0.13\\ 0.1&0.09&0.08&1.44&0.1&0.08&-0.2&-0.22&-0.2\\ 0.11&0.08&0.09&0.1&1.45&0.07&-0.13&-0.31&-0.14\\ 0.07&0.07&0.06&0.08&0.07&1.41&-0.19&-0.15&-0.19\\ -0.13&-0.18&-0.12&-0.2&-0.13&-0.19&1.9&0.15&0.47\\ -0.31&-0.16&-0.23&-0.22&-0.31&-0.15&0.15&2.22&0.21\\ -0.14&-0.18&-0.13&-0.2&-0.14&-0.19&0.47&0.21&1.86 \end{array}\displaystyle }\right] , \\ R&=\operatorname {diag} \begin{bmatrix} 0.63 &0.63 &93& 0.84& 0.63& 0.80& 1.51& 1.36& 1.39 \end{bmatrix}. \end{aligned}$$

**Fig. 13:**
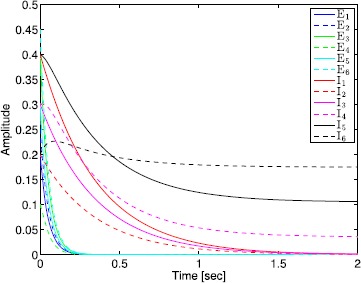
Solutions to (25) and (26) with initial conditions $S^{\mathrm{E}}(0)=[0.2, 0.25, 0.4, 0.1, 0.3, 0.45]^{\mathrm{T}}$, $S^{\mathrm{I}}(0)=[0.4, 0.2, 0.3, 0.3, 0.4, 0.2]^{\mathrm{T}}$ for $\lambda^{\mathrm{E}}=0.05\ \mathrm{s}$ and $\lambda^{\mathrm{I}}=0.35\ \mathrm{s}$. The synaptic drive of the excitatory neurons $\mathrm{E}_{1}$ to $\mathrm{E}_{6}$ and three of the inhibitory neurons $\mathrm{I}_{1}$, $\mathrm{I}_{2}$, and $\mathrm{I}_{3}$ converges to zero, whereas the synaptic drive of the inhibitory neurons $\mathrm{I}_{4}$, $\mathrm{I}_{5}$, and $\mathrm{I}_{6}$ that themselves do not receive inhibitory inputs do not converge to zero

Hence, the biological neural network given in (34) and (35) is globally exponentially partially synchronized. As can be seen in Fig. 13, the synaptic drive of the excitatory neurons $\mathrm{E}_{1}$ to $\mathrm{E}_{6}$ and three of the inhibitory neurons $\mathrm{I}_{1}$, $\mathrm{I}_{2}$, and $\mathrm{I}_{3}$ converges to zero, whereas the synaptic drive of the inhibitory neurons $\mathrm{I}_{4}$, $\mathrm{I}_{5}$, and $\mathrm{I}_{6}$ that themselves do not receive inhibitory inputs do not converge to zero.

**Fig. 14:**
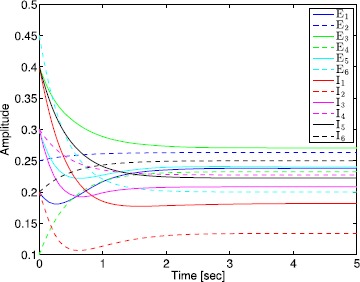
Solutions to (25) and (26) with initial conditions $S^{\mathrm{E}}(0)=[0.2, 0.25, 0.4, 0.1, 0.3, 0.45]^{\mathrm{T}}$, $S^{\mathrm{I}}(0)=[ 0.4, 0.2, 0.3, 0.3, 0.4, 0.2]^{\mathrm{T}}$ for $L^{\mathrm{E}}= \operatorname {diag}[2.1 , 1.9 , 1.85, 2.15, 2.05 , 2.5]$, $L^{\mathrm{I}}=\operatorname {diag}[1.5 , 1.75 , 2.4 , 2.2 , 2.25 , 2]$, and $f_{\max }=0.5$. The synaptic drive of the excitatory neurons $\mathrm{E}_{1}$ to $\mathrm{E}_{6}$ do not converge to zero

**Fig. 15:**
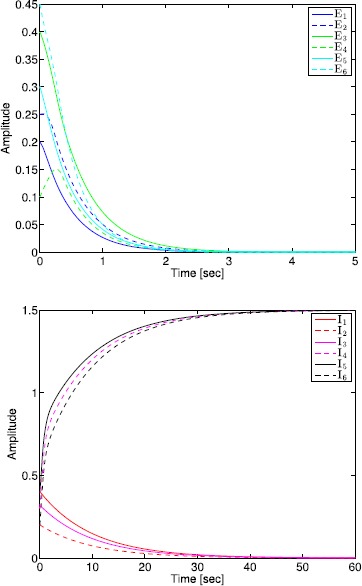
Solutions to (25) and (26) with initial conditions $S^{\mathrm{E}}(0)=[0.2, 0.25, 0.4, 0.1, 0.3, 0.45]^{\mathrm{T}}$, $S^{\mathrm{I}}(0)=[ 0.4, 0.2, 0.3, 0.3, 0.4, 0.2]^{\mathrm{T}}$ for $L^{\mathrm{E}}= \operatorname {diag}[2.1 , 1.9 , 1.85 , 2.15 , 2.05 , 2.5]$, $L^{\mathrm{I}}=\operatorname {diag}[0.1 , 0.1 , 0.1 , 0.1 ,0.1 , 0.1]$, and $f_{\max }=0.5$. The synaptic drive of the excitatory neurons $\mathrm{E}_{1}$ to $\mathrm{E}_{6}$ converge to zero

Next, we apply Theorem 3 to the connectivity matrix given by (126). Here, we assume that $\tilde{v}^{\mathrm{E}}_{\mathrm{th}}=[0.15, 0.15, 0.15, 0.15, 0.15, 0.15]^{\mathrm{T}}$, $\tilde {v}^{\mathrm{I}}_{\mathrm{th}}=[0.15, 0.15, 0.15, 0.15, 0.15, 0.15]^{\mathrm{T}}$, $L^{\mathrm{E}}=\operatorname {diag}[2.1, 1.9, 1.85, 2.15, 2.05, 2.5]$, $L^{\mathrm{I}}=\operatorname {diag}[1.5, 1.75, 2.4, 2.2, 2.25, 2]$, and the activation functions $f_{i}(\cdot)$, $i=1,2,\ldots,12$, are given by (52) with $f_{\max}=0.5$. As can be seen from Fig. 14, the synaptic drives of the excitatory neurons $\mathrm{E}_{1}$ to $\mathrm{E}_{6}$ do not converge to zero. However, when the time constants of the inhibitory neurons are increased to $L^{\mathrm{I}}=\operatorname {diag}[0.1 , 0.1, 0.1 ,0.1 ,0.1,0.1]$, the synaptic drives of the excitatory neurons $\mathrm{E}_{1}$ to $\mathrm{E}_{6}$ converge to zero; see Fig. 15.

Next, we present partial synchronization of the model presented in (79) and (80) with four excitatory neurons $\mathrm{E}_{1}$–$\mathrm{E}_{4}$ and four inhibitory neurons $\mathrm{I_{1}}$–$\mathrm{I_{4}}$. The neural connectivity matrix *A* is given by

127 $$\begin{aligned} A&= \small{\begin{bmatrix} 0 &1 &1 &1 &-1 &0 &-1 &-1 \\ 1 &0 &1 &1 &-1 &-1 &0 &-1\\ 1 &1 &0 &1 &0 &0 &-1 &-1\\ 1 &1 &1 &0 &0 &-1 &-1&-1\\ 1 &1 &1 &1 &0 &-1 &0 &0\\ 1 &1 &1 &1 &-1 &0 &0 &0\\ 1 &0 &1 &0 &0 &0 &0 &0\\ 0 &1 &0 &1 &0 &0 &0 &0 \end{bmatrix} }, \end{aligned}$$

which implies that the inhibitory neurons $\mathrm{I}_{3}$ and $\mathrm{I}_{4}$ do not receive inhibitory inputs. Here, we assume that all the excitatory neurons have the time constant $\lambda^{\mathrm{E}}=0.05\ \mathrm{s}$ and all the inhibitory neurons have the same prolonged time constant $\lambda ^{\mathrm{I}}=0.3\ \mathrm{s}$. Furthermore, we assume that $\tilde{v}^{\mathrm{E}}_{\mathrm{th}}=[0.02, 0.02, 0.02, 0.02]^{\mathrm{T}}$, $\tilde{v}^{\mathrm{I}}_{\mathrm{th}}=[0.02, 0.02, 0.4, 0.5]^{\mathrm{T}}$, and the activation functions $f_{i}(\cdot)$, $i=1,2,\ldots,8$, are given by (9).

The LMIs given by (88) and (89) are satisfied with

$$\begin{aligned} \hat{P}&= \left[ \textstyle\begin{array}{@{}c@{\ \ }c@{\ \ }c@{\ \ }c@{\ \ }c@{\ \ }c@{\ \ }c@{\ \ }c@{\ \ }c@{\ \ }c@{\ \ }c@{\ \ }c@{}} 0.2&0.04&0.04&0.04&0.03&0.03&0.11&0.06&0.06&0.06&0.03&0.03\\ 0.04&0.2&0.04&0.04&0.03&0.03&0.07&0.11&0.07&0.07&0.03&0.03\\ 0.04&0.04&0.2&0.04&0.03&0.03&0.06&0.06&0.11&0.06&0.03&0.03\\ 0.04&0.04&0.04&0.2&0.03&0.03&0.06&0.06&0.06&0.11&0.03&0.03\\ 0.03&0.03&0.03&0.03&4.8&0.73&0.04&0.04&0.04&0.04&2.97&1.62\\ 0.03&0.03&0.03&0.03&0.73&4.8&0.04&0.04&0.04&0.04&1.62&2.97\\ 0.11&0.07&0.06&0.06&0.04&0.04&0.19&0.05&0.05&0.05&0.04&0.04\\ 0.06&0.11&0.06&0.06&0.04&0.04&0.05&0.19&0.05&0.05&0.04&0.04\\ 0.06&0.07&0.11&0.06&0.04&0.04&0.05&0.05&0.19&0.05&0.04&0.04\\ 0.06&0.07&0.06&0.11&0.04&0.04&0.05&0.05&0.05&0.19&0.04&0.04\\ 0.03&0.03&0.03&0.03&2.97&1.62&0.04&0.04&0.04&0.04&3.29&1.35\\ 0.03&0.03&0.03&0.03&1.62&2.97&0.04&0.04&0.04&0.04&1.35&3.29 \end{array}\displaystyle \right], \\ \hat{Q}&= \left[ \textstyle\begin{array}{@{}c@{\ }c@{\ }c@{\ }c@{\ }c@{\ }c@{\ }c@{\ }c@{\ }c@{\ }c@{\ }c@{\ }c@{}} 16.9&1.1&1&1&0.5&1.9&-2.8&-4.3&-4.2&-4.2&-6.4&-6.3\\ 1.1&16.8&1&1.1&0.7&0.7&-4.1&-2.7&-4.1&-4.1&-6.3&-6.3\\ 1&1&16.8&1&1.8&1.8&-4.4&-4.4&-2.9&-4.4&-6.4&-6.4\\ 1&1.1&1&16.9&1.9&0.5&-4.2&-4.3&-4.2&-2.8&-6.3&-6.4\\ 0.5&0.7&1.8&1.9&14.3&3.3&-6.1&-6.1&-6.1&-6.1&4.5&1.9\\ 1.9&0.7&1.8&0.5&3.3&14.3&-6.1&-6.1&-6.1&-6.1&1.9&4.5\\ -2.8&-4.1&-4.4&-4.2&-6.1&-6.1&34.5&11.7&11.8&11.6&-7.1&-7.2\\ -4.3&-2.7&-4.4&-4.3&-6.1&-6.1&11.7&34.6&11.8&11.7&-7.1&-7.1\\ -4.2&-4.1&-2.9&-4.2&-6.1&-6.1&11.8&11.8&34.7&11.8&-7.2&-7.2\\ -4.2&-4.1&-4.4&-2.8&-6.1&-6.1&11.6&11.7&11.8&34.5&-7.2&-7.1\\ -6.4&-6.3&-6.4&-6.3&4.5&1.9&-7.1&-7.1&-7.2&-7.2&46.5&18.6\\ -6.3&-6.3&-6.4&-6.4&1.9&4.5&-7.2&-7.1&-7.2&-7.1&18.6&46.5 \end{array}\displaystyle \right], \\ \hat{R}&=\operatorname {diag}\left[ \textstyle\begin{array}{@{}c@{\ }c@{\ }c@{\ }c@{\ }c@{\ }c@{\ }c@{\ }c@{\ }c@{\ }c@{\ }c@{\ }c@{}} 19.8& 19.8& 19.8& 19.8& 22.73& 22.73& 3.55& 3.24& 3.34& 3.55& 3.87& 3.87\end{array}\displaystyle \right]. \end{aligned}$$

**Fig. 16:**
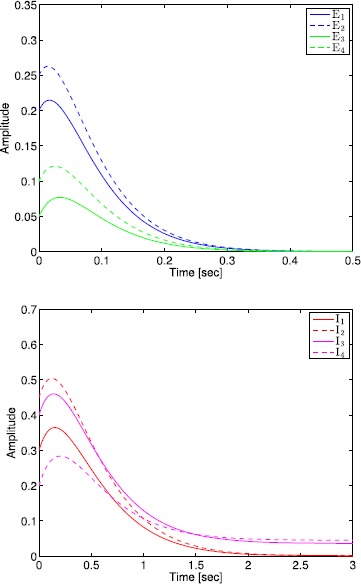
Solutions to (72) and (73) with initial conditions $S^{\mathrm{I}}(0)=[0.2, 0.25, 0.05, 0.1]^{\mathrm{T}}$, $S^{\mathrm{I}}(0)=[ 0.3, 0.45, 0.4, 0.2]^{\mathrm{T}}$, $\dot{S}^{\mathrm{E}}(0)=[2, 2, 2, 2]^{\mathrm{T}}$, $\dot{S}^{\mathrm{I}}(0)=[1, 1, 1, 1]^{\mathrm{T}}$ for $\lambda^{\mathrm{E}}=0.05\ \mathrm{s}$ and $\lambda^{\mathrm{I}}=0.3\ \mathrm{s}$. The synaptic drive of the excitatory neurons $\mathrm{E}_{1}$ to $\mathrm{E}_{4}$ and two of the inhibitory neurons $\mathrm{I}_{1}$ and $\mathrm{I}_{2}$ converges to zero, whereas the synaptic drive of the inhibitory neurons $\mathrm{I}_{3}$ and $\mathrm{I}_{4}$ that themselves do not receive inhibitory inputs do not converge to zero

Hence, the biological neural network given in (79) and (80) is globally exponentially partially synchronized. As can be seen in Fig. 16, the synaptic drive of the excitatory neurons $\mathrm{E}_{1}$ to $\mathrm{E}_{4}$ and two of the inhibitory neurons $\mathrm{I}_{1}$ and $\mathrm{I}_{2}$ converges to zero, whereas the synaptic drive of the inhibitory neurons $\mathrm{I}_{3}$ and $\mathrm{I}_{4}$ do not converge to zero.

**Fig. 17:**
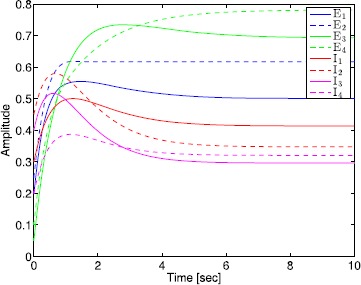
Solutions to (72) and (73) with initial conditions $S^{\mathrm{I}}(0)=[0.2, 0.25, 0.05, 0.1]^{\mathrm{T}}$, $S^{\mathrm{I}}(0)=[ 0.3, 0.45, 0.4, 0.2]^{\mathrm{T}}$, $\dot{S}^{\mathrm{E}}(0)=[0.5, 0.5, 0.5, 0.5]^{\mathrm{T}}$, $\dot{S}^{\mathrm{I}}(0)=[0.5, 0.5, 0.5, 0.5]^{\mathrm{T}}$ for $L^{\mathrm{E}}=\operatorname {diag}[1 ,0.9 , 0.85 , 0.8]$, $L^{\mathrm{I}}=\operatorname {diag}[1.1 , 1.2 , 1.3 , 1.25]$, and $f_{\max}=0.5$. The synaptic drive of the excitatory neurons $\mathrm{E}_{1}$ to $\mathrm{E}_{4}$ do not converge to zero

**Fig. 18:**
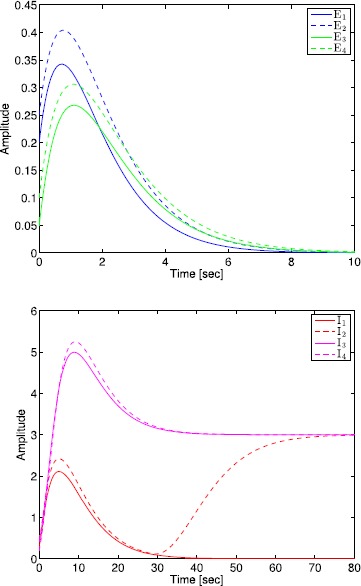
Solutions to (72) and (73) with initial conditions $S^{\mathrm{I}}(0)=[0.2, 0.25, 0.05, 0.1]^{\mathrm{T}}$, $S^{\mathrm{I}}(0)=[ 0.3, 0.45, 0.4, 0.2]^{\mathrm{T}}$, $\dot{S}^{\mathrm{E}}(0)=[0.5, 0.5, 0.5, 0.5]^{\mathrm{T}}$, $\dot{S}^{\mathrm{I}}(0)=[0.5, 0.5, 0.5, 0.5]^{\mathrm{T}}$ for $L^{\mathrm{E}}=\operatorname {diag}[1 ,0.9 , 0.85 , 0.8]$, $L^{\mathrm{I}}=\operatorname {diag}[0.2 , 0.2 , 0.2 , 0.2]$, and $f_{\max}=0.5$. The synaptic drive of the excitatory neurons $\mathrm{E}_{1}$ to $\mathrm{E}_{4}$ converge to zero

Next, we apply Theorem 5 to the connectivity matrix given by (127). Here, we assume that $\tilde{v}^{\mathrm{E}}_{\mathrm{th}}=[0.12, 0.12, 0.12, 0.12]^{\mathrm{T}}$, $\tilde{v}^{\mathrm{I}}_{\mathrm{th}}=[0.12, 0.12, 0.12, 0.12]^{\mathrm{T}}$, $L^{\mathrm{E}}= \operatorname {diag}[1, 0.9, 0.85, 0.8]$, $L^{\mathrm{I}}= \operatorname {diag}[1.1, 1.2, 1.3, 1.25]$, and the activation functions $f_{i}(\cdot)$, $i=1,2,\ldots,8$, are given by (52) with $f_{\max}=0.5$. As can be seen from Fig. 17, the synaptic drives of the excitatory neurons $\mathrm{E}_{1}$ to $\mathrm{E}_{4}$ do not converge to zero. Once again, however, as we increase the time constants of the inhibitory neurons to $L^{\mathrm{I}}=\operatorname {diag}[0.2 , 0.2, 0.2 , 0.2]$, the synaptic drives of the excitatory neurons $\mathrm{E}_{1}$ to $\mathrm{E}_{4}$ converge to zero; see Fig. 18.

It can also be seen from Figs. 16 and 18 that the synaptic drive of the excitatory neurons $\mathrm{E}_{1}$ to $\mathrm{E}_{4}$ increase initially before converging to zero. This potentially captures the drug biphasic response, where there is a boost in brain activity prior to hypnotic induction.

**Fig. 19:**
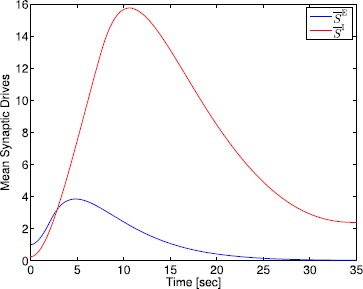
Solutions to (124) and (125) for $n_{\mathrm{E}}\overline{A}^{\mathrm{EE}}=10$, $n_{\mathrm{I}}\overline{A}^{\mathrm{II}}=12$, $n_{\mathrm{E}}\overline{A}^{\mathrm{IE}}=4$, $n_{\mathrm{I}}\overline{A}^{\mathrm{II}}=1$, $v_{\mathrm{th}}^{\mathrm{E}}=0$, $v_{\mathrm{th}}^{\mathrm{I}}=0$, $\lambda^{\mathrm{E}}=4\ \mathrm{s}$, $\lambda^{\mathrm{I}}=6\ \mathrm{s}$, $f_{\max}=1$, and $\gamma=1$ with initial conditions $\overline{S}^{\mathrm{E}}(0)=1$, $\overline{S}^{\mathrm{I}}(0)=0.5$, and $\dot {\overline{S}}^{\mathrm{E}}(0)=\dot{\overline{S}}^{\mathrm{E}}(0)=0$

Finally, we further explore the drug biphasic phenomena using the second-order mean excitatory and mean inhibitory synaptic drive model given by (124) and (125) with the activation function $f(\cdot)$ given by (10). Once again, we observe that there is an initial boost in the mean excitatory synaptic drive just before convergence to zero; see Fig. 19.

## Conclusion and Discussion

Over the past decade there have been significant advances in our understanding of how anesthetic agents affect the properties of neurons [1, 2]. This has led, in turn, to efforts to understand how molecular mechanisms translate into the induction of general anesthesia at the macroscopic level [3–7, 12–17]. In particular, since the pioneering work of Steyn-Ross et al. [12] there has been particular focus on how the molecular properties of anesthetic agents lead to the observed changes in the electroencephalogram that are associated with the induction of anesthesia [12–17]. These models, of necessity, have focused on the electrochemical potential of the neuron and the analyses consider both the temporal and the spatial domain, specifically invoking a continuous spatial domain to account for interneuron connectivity. The complexity of the brain requires specific assumptions as regards this connectivity.

In this paper, we used a synaptic drive formulation of neuronal activity to derive a mechanistic model that can be employed to understand the effects of anesthetic agents on neuronal firing rates. Our focus is on demonstrating a decrease in the firing rate of excitatory neurons as a consequence of the known effects of anesthetic agents on single neurons. Thus, there is the implicit assumption that anesthesia is a result of decreased neuronal activity. We do note that this may be overly simplistic since the induction of anesthesia is associated with an initial increase in neuronal activity. However, we seek to understand how decreased activity might develop because (i) anesthetic agents at sufficiently high concentrations will cause a decrease in neuronal activity and (ii) one theory of anesthesia is that it represents the interruption of flow of “information” through the brain and this could certainly result from decreased neuronal activity in specific regions.

Even this seemingly simple effect cannot be demonstrated using the full synaptic drive model. Our model begins with a spatially discrete description of neuronal connectivity. However, given the immense dimensionality of the brain, in the first part of the paper we simplify our model using a mean field assumption. Specifically, we assume that within the populations of excitatory and inhibitory neurons, second-order terms that are the product of variances of synaptic connection strengths and variances of synaptic drives (from the mean) can be ignored [5].

We show that even this simplified two-state mean field theory exhibits interesting behavior. In particular, for certain values of model parameters, the mean excitatory synaptic drive has an asymptotically stable equilibrium point. Then as the time constant of the inhibitory postsynaptic potential increases, an effect of certain anesthetic agents on the single neuron, this equilibrium point becomes unstable, giving rise to a stable limit cycle. With further increases in the inhibitory time constant the limit cycle disappears and we again find an asymptotically stable equilibrium point corresponding to a decreasing mean excitatory synaptic drive.

This offers a new interpretation of the stochastic characteristic of the induction of anesthesia. There have been animal studies, and also a small number of human studies, in which the response of a single subject to varying doses of an anesthetic agent are observed. In these studies, it has been observed that the concentration-response curve is very sharp [30]. In particular, as the dose or concentration of the anesthetic agent increases there exists a point at which there is an abrupt transition from consciousness to unconsciousness. However, in these studies the concentration-response curve has not been found to be a strict step function. Rather, there is a narrow range of concentrations within which the therapeutic effect (response or lack of a response to a noxious stimulus) has been described as a probability of response.

A common empirical model describing this effect is a variation on the Hill equation [31] in which the probability *P* of an anesthetic effect, that is, of no response to a noxious stimulus, is given by

128 $$ P=\frac{C^{\gamma}}{[C_{50}^{\gamma}+C^{\gamma}]}, $$

where *C* is the concentration of anesthetic agent, $C_{50}$ is the concentration of anesthetic agent associated with a $50\ \%$ probability of an anesthetic effect, and *γ* is a parameter that determines the steepness of the concentration–probability anesthetic curve. Typically for individual subjects *γ* ranges from 6 to 20 [32, 33].

The range of drug concentrations where we cannot predict with certainty whether the patient will respond to noxious stimuli or not (requiring a probabilistic model) could certainly be explained by stochastic variation in the postulated inputs from pain, sensorimotor, and proprioceptive sensors, or the reticular activating system. However, an alternative explanation is that during the induction of anesthesia the probability of observing the response of the patient depends on the time in the cycle at which the observation is made. The patient will respond if the observation occurs at the time when the mean excitatory synaptic drive is at its peak and will not respond if the mean excitatory synaptic drive is at its nadir. If the concentration of the anesthetic drug is further increased, corresponding to a further increase in the inhibitory postsynaptic potential time constant, the limit cycle disappears and the mean excitatory synaptic drive has an asymptotically stable equilibrium that approaches zero (Fig. 10) and at this point, the patient is deeply anesthetized (i.e., sedated) and will not respond.

The most formidable challenge to understanding the transition to anesthesia is the immense dimensionality of the brain. Typically, this dimensionality is simplified with either specific structural assumptions or by using mean field (or more sophisticated statistical) frameworks. The extent to which these assumptions influence even qualitative conclusions is unclear. In the case of the mean synaptic drive model that we use in this paper, we have simplified the analysis by ignoring second-order terms in an expansion around mean values. Without this assumption, we are unable to reach any analytical conclusions regarding excitatory neuronal activity with an increasing inhibitory postsynaptic potential time constant. It is not clear how the mean field approximation impacts our conclusions.

In the second part of the paper we have accordingly extended our analysis without mean field assumptions by postulating the existence of a subset of inhibitory neurons that themselves do not receive inhibitory inputs. Using this assumption we derived sufficient conditions for convergence and state equipartition of the activity of excitatory neurons receiving input from these inhibitory neurons to zero. We further extended this analysis to consider a nonmonotonic postsynaptic potential, as an alternative and possibly more realistic model for postsynaptic potentials. An assumption that is essential to this finding of our paper is the existence of a subset of inhibitory neurons that themselves do not receive inhibitory input. We view this postulate as a more transparent assumption for the synaptic drive model than the mean field assumption. We are unaware of any experimental verification of such a subset. In the absence of such data, we view this assumption as defining a sufficient condition for partial state equipartitioning of a subset of excitatory neurons to zero activity. The convergence of activity of subset(s) of excitatory neurons would certainly be a plausible explanation for the interruption of information flow in the brain that is one suggested mechanism for unconsciousness [20].

The proposed dynamical system framework can potentially foster the development of new frameworks that can allow us to interpret experimental and clinical results, connect biophysical findings to psychophysical phenomena, explore a new hypothesis based on the cognitive neuroscience of consciousness and develop new assertions, and improve the reliability of general anesthesia. Dynamical systems theory is ideally suited for rigorously describing the behavior of large-scale networks of neurons and can potentially establish a scientific basis for new metrics of anesthetic depth by making the assessment of consciousness a mechanistically grounded tool.
